# Expanding the Toolbox for Inducible Protein Expression With Automation‐enabled Generation of Glycomimetics

**DOI:** 10.1002/chem.202502010

**Published:** 2026-01-07

**Authors:** Ashley E. DeYong, Keevan C. Marion, Murat Ozturk, Sanjeeva Kumar Murali, Fatima Enam, Thomas J. Mansell, Nicola L. B. Pohl

**Affiliations:** ^1^ Chemistry Indiana University Bloomington Indiana USA; ^2^ Chemical and Biological Engineering Iowa State University Ames Iowa USA

**Keywords:** automation, carbohydrates, continuous flow, protein expression, synthetic biology

## Abstract

Inducible protein expression is a cornerstone of many aspects of industrial and molecular biotechnological processes. However, limited availability of inducible transcription factors can reduce our ability to control expression at a population level. The design and synthesis of a powerful inducer containing a fucose is demonstrated to induce protein expression through the *lac* operon only in cells with the ability to selectively de‐fucosylate them. Batch and automated continuous‐flow processes are reported for the syntheses of both 2′‐fucosyl isopropyl‐β‐D‐thiogalactopyranose (2′F‐IPTG) and isobutyl‐C‐galactoside (2′F‐IBCG) mimics. Fucosylation of the inducer allowed for fucosidase‐dependent expression of a reporter protein, providing an additional layer of control over inducible gene expression.

## Graphical abstract

Inducible protein expression is a cornerstone of many aspects of industrial and molecular biotechnological processes. 2′‐fucosyl isopropyl‐β‐D‐thiogalactopyranose (2′F‐IPTG) and 2′‐fucosyl isobutyl‐C‐galactoside (2′F‐IBCG) mimics were explored for protein expression studies. Herein, we report the automated continuous flow syntheses of the mimics and their utility in protein expression.

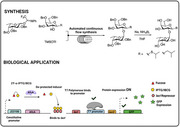

## 1 Introduction

Control of recombinant protein expression is critical for optimal production of protein therapeutics and other high‐value molecules [1]. Cells must balance biomass formation with production of the proteins or pathways of interest, which requires precise spatiotemporal control of induction [2]. In addition, protein overexpression causes aggregation, misfolding, and stability issues [3]. To overcome these challenges, inducible gene expression systems aid in tight control of protein production, such as the control of the industry standard inducible T7 gene expression system via the *lac* operon, widely used for the production of recombinant proteins in *Escherichia coli* and other prokaryotic hosts [4].

Many inducible expression systems are activated by carbohydrates, e.g., lactose, rhamnose, and arabinose [5]. Such systems often suffer from catabolism of the inducer and cross‐talk with other metabolic pathways. In addition, the co‐expression of metabolic pathways in microbial communities for division of labor is increasingly recognized as a promising strategy for mitigating the metabolic burden of complex biomolecules [6]. Thus, there is a need for inducible expression systems that are strain‐specific and protected from degradation. A prominent example of strain‐specific utilization of carbohydrate substrates is found in the human milk oligosaccharides (HMOs). They consist of three to five important monosaccharides (glucose, galactose, glucosamine, sialic acid, and fucose) that link together to form more than two hundred complex structures [7, 8, 9]. The specificity of these carbon sources for certain species comes from that species’ ability to hydrolyze the carbohydrate linkage into fermentable sugars (i.e., lactose), thus it follows that certain HMOs favor the growth of specific members of the gut microbial community. While studies have shown the key biological function of specific HMOs, understanding the structure to function relationship is currently ongoing research [10, 11, 12, 13, 14, 15, 16, 17, 18, 19, 20, 21, 22, 23, 24, 25, 26]. However, this work is currently hindered by limitations in separation, analytical identification, and gram scale production techniques [27, 28, 29, 30, 31, 32, 33, 34, 35, 36, 37, 38, 39, 40, 41, 42, 43, 44, 45, 46, 47, 48].

**FIGURE 1:**
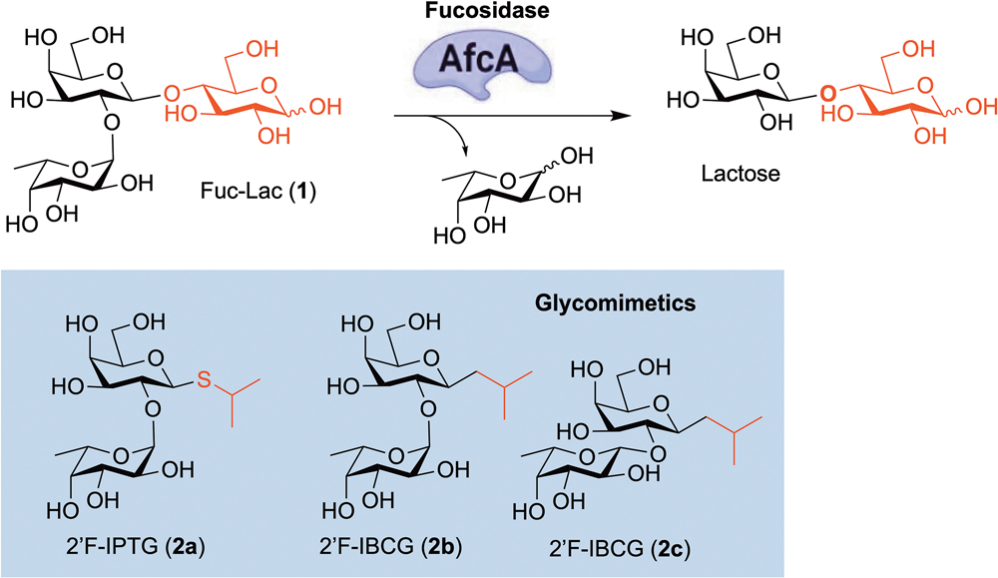
Use of synthesized glycomimetics as stable inducers of protein expression.

2′‐Fucosyllactose is the most abundant oligosaccharide in human milk, and such fucosylated HMOs display properties of anti‐adhesive agents [49, 50, 51, 52]. In addition to these important biological properties, 2′‐fucosyllactose has also shown promise for its use in protein expression work dependent on a cell's ability to enzymatically remove the fucose [53]. However, the presence of β‐galactosidase (which is required for autoinduction using lactose) can also result in cleavage of the glycosidic bond between galactose and glucose, reducing induction potential. This problem suggests the need to explore potential mimics that allow for protein expression but are independent of the presence of β‐galactosidase. Herein, we report the design, synthesis, and evaluation in a protein induction system of two fucosylated mimics, 2′‐fucosyl isopropyl‐β‐D‐thiogalactopyranose (2′F‐IPTG, **2a**) and 2′‐fucosyl isobutyl‐*C*‐galactoside (2′F‐IBCG, **2b, 2c**), where the glucose is replaced with an *S*‐ or *C*‐linked moiety (Figure 1).

IPTG has previously been used in the production of recombinant proteins via plasmid‐based gene expression by binding the lac repressor of *Escherichia coli* [54, 55] and therefore served as the starting point for our designs. Known limitations of IPTG in gene expression work include the degradation of the molecule under culture conditions, resulting in the enzymatic oxidation and cleavage of the anomeric carbon‐sulfur bond. More stable carbon‐carbon bonds at the anomeric position of galactose (IBCG), rather than sulfur bonds, have also been shown to be tolerated by these *lac* protein expression systems. [56, 57] The use of these two galactose analogs (IPTG and IBCG) in gene expression and protein expression work point to the possibility of incorporating such modifications in the design of new 2′‐fucosyllactose analog mimetics for protein expression.

In addition to addressing such biological challenges, the synthesis of HMO analogs and the production of sufficient quantities of material for biological use remains challenging [58, 59, 60, 61]. Solution‐phase automated methods, which have been previously used towards the synthesis of a variety of glycosylated targets [61, 62, 63, 64, 65], address some synthetic challenges in the field of carbohydrate synthesis. Automated continuous‐flow processes using easily accessible and widely available parts and equipment, such as syringe pumps and HPLC pumps and open‐source software, have the potential to make accessing HMOs synthetically easier, more reproducible, and readily scalable for the needs of biological applications [66, 67, 68, 69].

**SCHEME 1:**
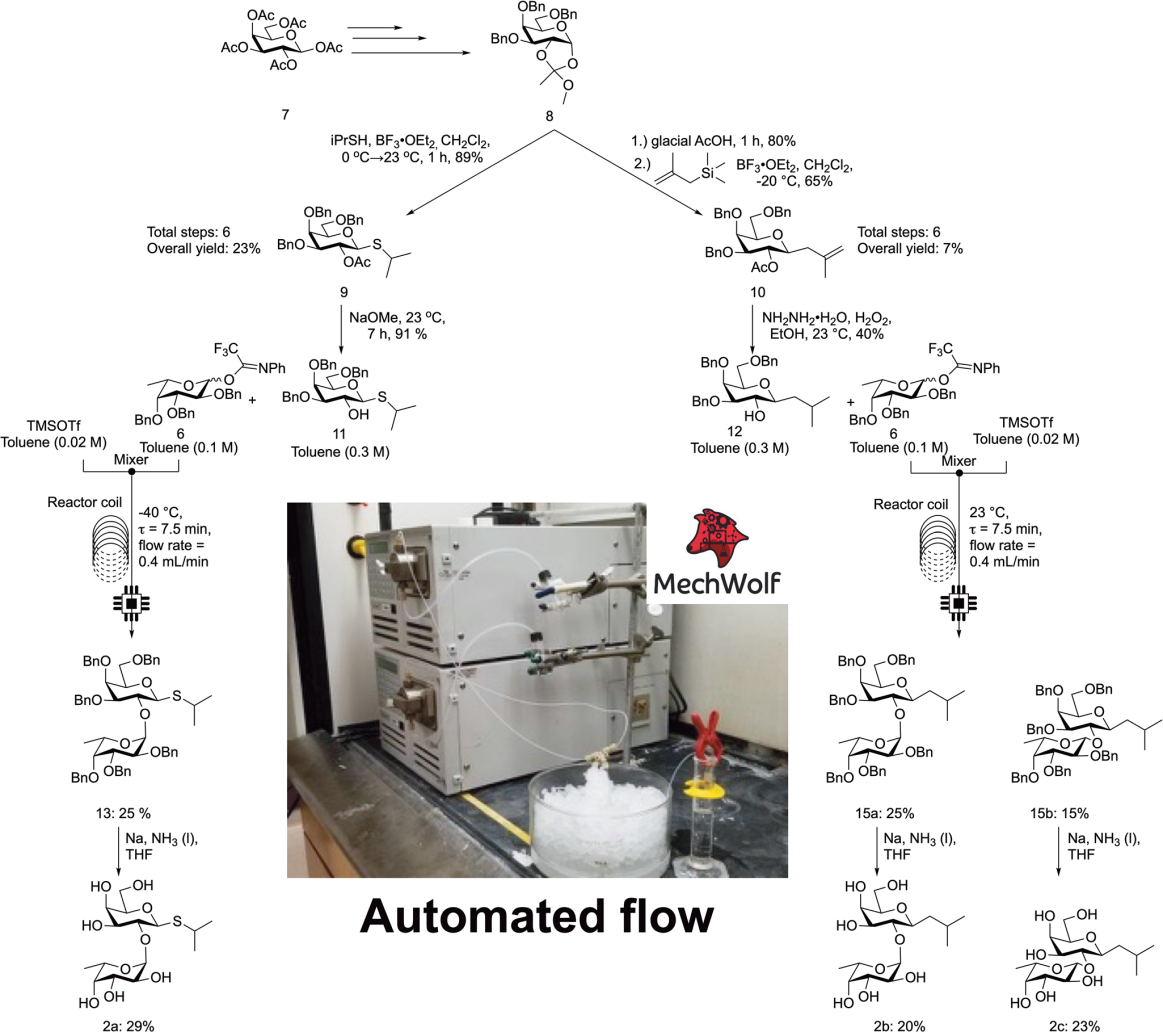
Generation of 2′‐fucosyllactose 2′F‐IPTG **13** and 2′F‐IBCG **15a, 15b** mimics from commercially available compound **7** utilizing batch and continuous flow processes.

## 2 Results and Discussion

To find a stable inducer or protein expression that is dependent on the presence of enzymes that cleave fucose, the mimics first were synthesized. In the construction of the acceptor monosaccharide, the known compound **8** [70, 71] was synthesized followed by the 2‐propanethiol installation under batch processes followed by deacetylation under an automated continuous flow process (see SI for flow optimization and use of static mixer), providing the 2′‐fucosyl IPTG acceptor building block, compound **11** (Scheme 1). For the construction of compound **12** under batch processes, compound **8** was used as a common intermediate, and the orthoester was first opened using glacial acetic acid and was then coupled with methylallyltrimethylsilane to give the *C*‐linked monosaccharide building block, compound **10**. Using hydrazine monohydrate, compound **10** was deacetylated and the alkene on the anomeric group was reduced in a single step to provide the final 2′‐fucosyl IBCG acceptor building block, compound **12**.

After exploration of various glycosyl donors (see SI for reaction optimization information), compounds **13**, **15a,** and **15b** were constructed using an automated continuous flow process in moderate yields, which utilized a self‐documenting MechWolf program for remote control of the flow system, as well as an electronic lab notebook for recording reaction parameters. Glycosylated compounds were then globally deprotected under batch processes using Birch reduction conditions, giving target compounds **2a**, **2b**, and **2c**. Birch reduction conditions were necessary as Pd catalyzed hydrogenation of a test substrate under continuous flow process using H‐Cube hydrogenation system showed to be unsuccessful (Table S1). Traditional Pd catalyzed hydrogenation has shown to poison the catalyst due to the thiol functionality [72].

**FIGURE 2:**
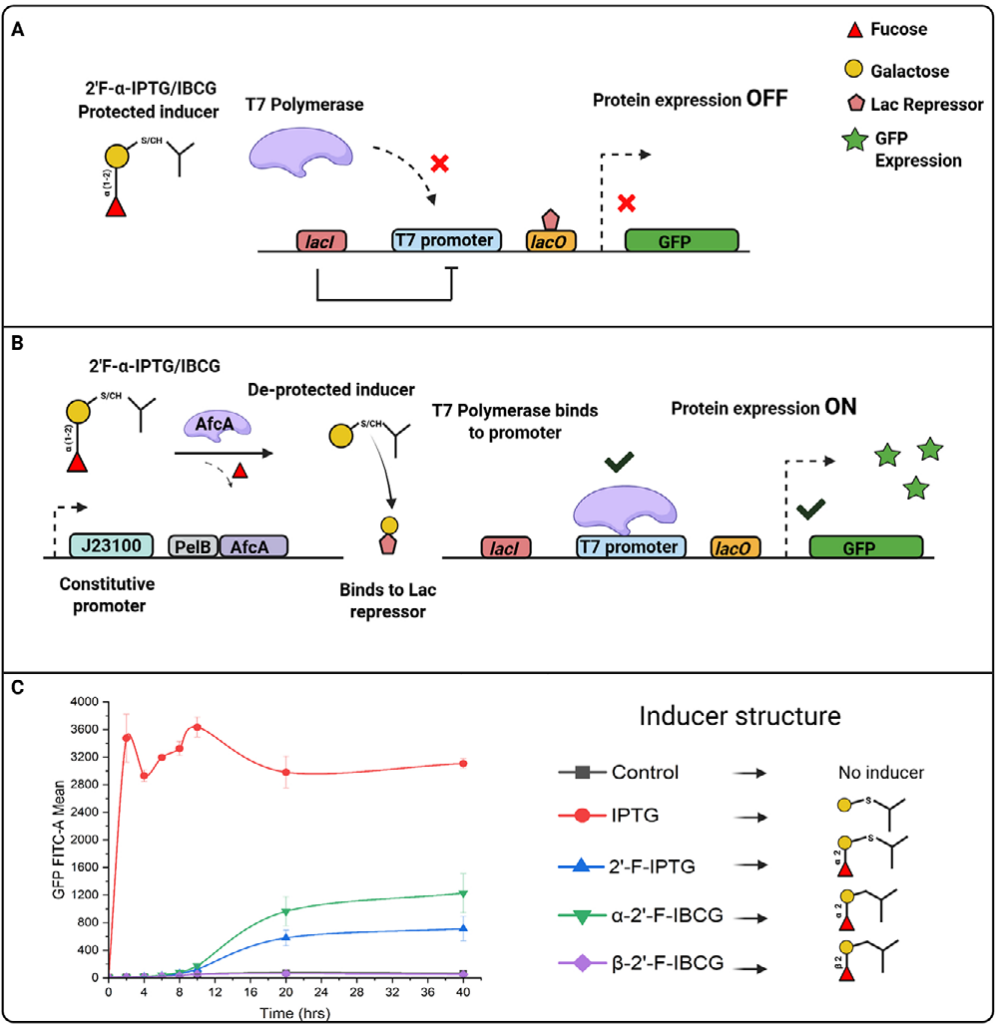
2′‐Fucosyl IPTG and IBCG mimics of 2′‐fucosyllactose can be used to induce protein expression in *E. coli* cells. (a) The fucosylated inducer does not bind LacI and therefore does not induce reporter protein expression via the T7 promoter. (b) In the presence of α1,2‐fucosidase AfcA, the IPTG/IBCG moiety is released and can bind the LacI repressor, allowing protein expression. (c) Dynamics of GFP expression using fucosylated and nonfucosylated inducers. Cells were incubated with 1 mM inducer and GFP fluorescence was measured over 40 hours.

With the two desired analogs in hand, the fucosylated inducers were tested for their ability to express protein in the industry standard T7 expression system as in previous work with 2′‐fucosyllactose [53]. We induced cultures of BL21 (DE3) *Escherichia coli* cells harboring plasmid pET28:GFP, which contains the gene for green fluorescent protein under control of the T7 promoter. In this strain, the T7 RNA polymerase is under control of the *Lac* promoter and inducible by IPTG analogs (Figure 2a/b). We hypothesized that protecting IPTG or IBCG with fucose (by analogy to HMOs) would provide induction of protein expression only in organisms which are engineered to deprotect it [73], so cells were cultured with a plasmid expressing the α1,2‐fucosidase AfcA from *Bifidobacterium bifidum*, secreted to the bacterial periplasm via the ssPelB signal sequence [53].

Figure 2c shows that as expected, IPTG induction leads to high levels of GFP expression. The 2′‐fucosyl‐IPTG (**2a**) also showed induction of GFP activity, albeit at a lower level on an equimolar basis. Interestingly, we also notice a lag in expression. In the previously referenced work, we observed a similar lag using 2′‐fucosyllactose as an inducer, and the lag here could be attributable to differences in substrate specificity between the native substrate of AfcA and our synthetic analogs, since transport limitations to the periplasm are likely to be minimal for molecules under 600 Da [74]. **2b** also showed substantial GFP induction, higher than **2a**, while **2c**, which contains a β1,2 linkage showed no induction as it is not acted upon by AfcA [75]. Better induction for **2b** could be linked to its increased stability over IPTG as previously demonstrated [56]. In this previously demonstrated work, IPTG was known to degrade under culture conditions through enzymatic oxidation and cleavage of the anomeric carbon‐sulfur bond, whereas the carbon‐carbon bond of IBCG has been shown to be tolerated by these *lac* protein expression systems. To determine whether induction was specific to cells expressing AfcA, we added inducers to BL21 cells with and without the AfcA expression plasmid. Supplementary Fig. S5 shows that only cells expressing AfcA showed GFP protein expression, confirming the requirement for hydrolysis of the inducer for functional induction. Hence, the synthesized 2′‐fucosyl IPTG and IBCG analogs can be used as a cell‐specific inducer for T7 protein expression.

## 3 Conclusion

The demonstration that induction of heterologous protein expression is dependent on enzymatic hydrolysis of inducers has broad implications for the use of these inducers in microbial communities. For example, co‐cultures are often utilized in metabolic engineering to distribute workload among several strains [76, 77, 78] and selective induction could provide another level of control to optimize production by T7 promoters. In addition, in the context of the gut microbiome, engineered live biotherapeutics often rely on dynamic control of protein expression [79] and the ability to selectively induce expression in a complex microbial community could lead to greater control of therapeutic effects [80]. Overall, automation‐guided glycosylation of small molecule inducers expands the repertoire of inducible expression and provides an additional layer of control that can be exploited in both monocultures and microbial communities.

## Conflicts of Interest

The authors declare no conflict of interest.

## Supporting information



Complete details of the synthesis and protein expression system, including NMR spectra, are included in the supporting information [81, 82, 83, 84, 85, 86, 87, 88, 89, 90, 91, 92].
**Supporting File 1**: chem70539‐sup‐0001‐SuppMat.pdf.

## Data Availability

The data that support the findings of this study are available in the supplementary material of this article.
