# Supplementary material for: Expanding the Toolbox for Inducible Protein Expression With Automation‐enabled Generation of Glycomimetics
Source: Chemistry. 2026 Jan 7;32(10):e02010. doi: 10.1002/chem.202502010 (PMC12995849; doi:10.1002/chem.202502010)
Supplement: Supplementary file 1 — Complete details of the synthesis and protein expression system, including NMR spectra, are included in the supporting information [81, 82, 83, 84, 85, 86, 87, 88, 89, 90, 91, 92]. Supporting File 1: chem70539‐sup‐0001‐SuppMat.pdf. [file CHEM-32-e02010-s001.pdf]

Supporting Information
©Wiley-VCH 2021
69451 Weinheim, Germany

Expanding the toolbox for inducible protein expression with automation enabled generation of glycomimetics

Ashley E. DeYong,[+a] Keevan C. Marion,[+a] Murat Ozturk,[a] Sanjeeva Kumar Murali,[b] Fatima Enam [b], Thomas J. Mansell,[b] and Nicola L. B. Pohl [*a]

Abstract: Inducible protein expression is a cornerstone of many aspects of industrial and molecular biotechnological processes. However, limited availability of inducible transcription factors can reduce our ability to control expression at a population level. The design and synthesis of a powerful inducer containing a fucose is demonstrated to induce protein expression through the *lac* operon only in cells with the ability to selectively de-fucosylate them. Batch and automated continuous-flow processes are reported for the syntheses of both 2'-fucosyl isopropyl-β-D-thiogalactopyranose (IPTG) and isobutyl-C-galactoside (IBCG) mimics. Fucosylation of the inducer allowed for fucosidase-dependent expression of a reporter protein, providing an additional layer of control over inducible gene expression.

DOI: 10.XXXXXXX

Table of Contents

TABLE OF CONTENTS 2

EXPERIMENTAL PROCEDURES 3

GENERAL EXPERIMENTAL INFORMATION 3

DESIGN AND CONSTRUCTION OF FLOW APPARATUS 3

SYNTHETIC PROCEDURES 6

^{1}H NMR, ^{13}C NMR, DQCOSY, HMBC, HSQC, HRMS OF NEW COMPOUNDS 25

INDUCIBLE PROTEIN EXPRESSION 117

SUPPORTING INFORMATION

Experimental Procedures

General Experimental Information

All solvents used for air- and moisture-sensitive reactions were high purity reagent grade. Solvent was collected from a solvent tower followed by the addition of oven-dried room temperature (~23 °C) 4Å molecular sieves and placed under argon gas via a syringe, balloon and septum contained in an Erlenmeyer flask or glass bottle and stored for 12 hours before use. Compounds were dissolved in anhydrous solvent prior to uptake in the HPLC pumps. Thin layer chromatography (TLC) was performed using Sorbent Technologies silica gel TLC plates, glass-backed and pre-coated with a thickness of 0.25 mm. After TLC development, TLC plates were visualized using UV light followed by *p*-anisaldehyde solution containing absolute ethanol and sulfuric acid (1:18:1, *p*-anisaldehyde:ethanol:sulfuric acid). Flash silica gel chromatography was carried out using the Teledyne ISCO CombiFlash® purification system (Combi flash R_f 200 and 200i) with preloaded silica columns and operated under the conditions stated for the column used.

Proton nuclear magnetic resonance (^{1}H NMR) and carbon nuclear magnetic resonance (^{13}C NMR) were performed on either 1) a Varian 500 MHz NMR containing a dual pulsed field gradient (PFG) probe with proton (^{1}H) signal to noise ratio 390 to 1, carbon (^{13}C) signal to noise ratio 290 to 1, and temperature range from -80 to 120 °C, 2) a Varian 400 MHz NMR containing a pulsed field gradient (PFG) probe with proton (^{1}H) signal to noise ratio 175 to 1, carbon (^{13}C) signal to noise ratio 160 to 1, and temperature range from -80 to 130 °C, or a Bruker 500 MHz NMR. Proton, carbon, and 2D analyses (dqCOSY, HMBC, HSQC) were performed and recorded in parts per million (ppm) and the residual signal of chloroform ($CDCl_3$) (δ 7.26 ppm ^{1}H NMR; δ 77.0 ppm ^{13}C NMR) was used as reference. ESI-MS Agilent 1200 HPLC-6130 MSD was used for mass analysis of synthesized compounds.

Design and Construction of Flow Apparatus

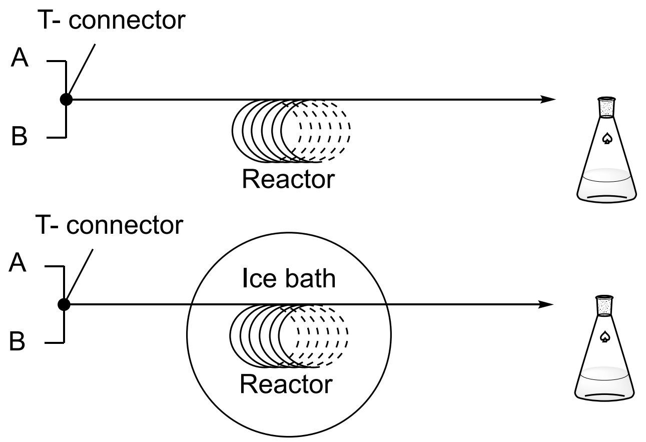

Figure 1 SI. Schematic of flow apparatus used in the syntheses.

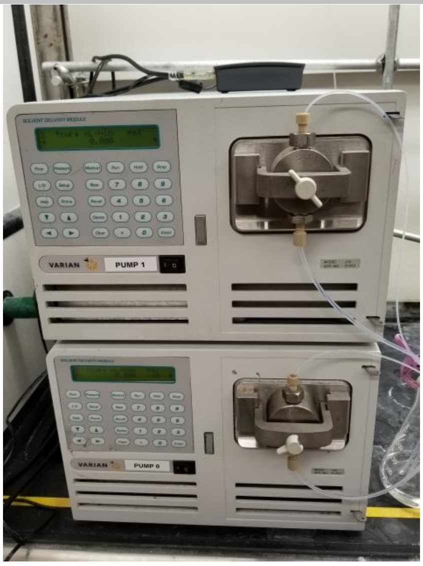

Figure 2 SI. HPLC Varian pump model 210

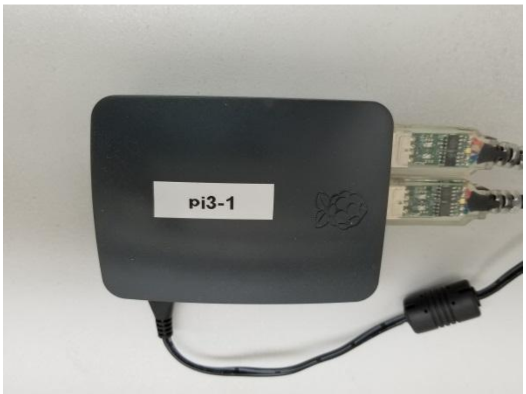

Figure 3 SI. Raspberry Pi with WiFi

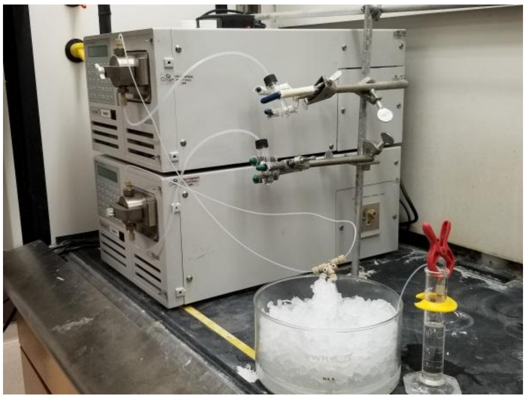

Figure 4 SI. Automated continuous-flow setup with ice-bath (ice-bath conditions can be changed for a -40 ^{o}C setup)

<u>Automated Continuous-flow equipment:</u>

HPLC pump Varian model 210

5 mL/min max pump heads

Raspberry Pi with WiFi

MechWolf program

Perfluoroalkoxy (PFA) tubing reactor coil (0.02 I.D., 1/16 O.D., 1524 cm (50 ft), 3 mL)

Perfluoroalkoxy (PFA) tubing (0.062 I.D., 1/8 O.D., 41.91 cm, 0.66 mL)

Perfluoroalkoxy (PFA) tubing (0.02 I.D., 1/16 O.D., 51.4 cm, 0.1 mL)

Flangeless fittings (1/16 in)

Flangeless fittings (1/8 in)

Ferrule (1/16 in)

Ferrule (1/8 in)

T connector (0.02 in I.D.)

Scheme 1 SI. Synthesis of *n*-Propyl-2,3,4-tri-O-benzyl-1-thio-β-L-fucopyranoside and acyl derivative.

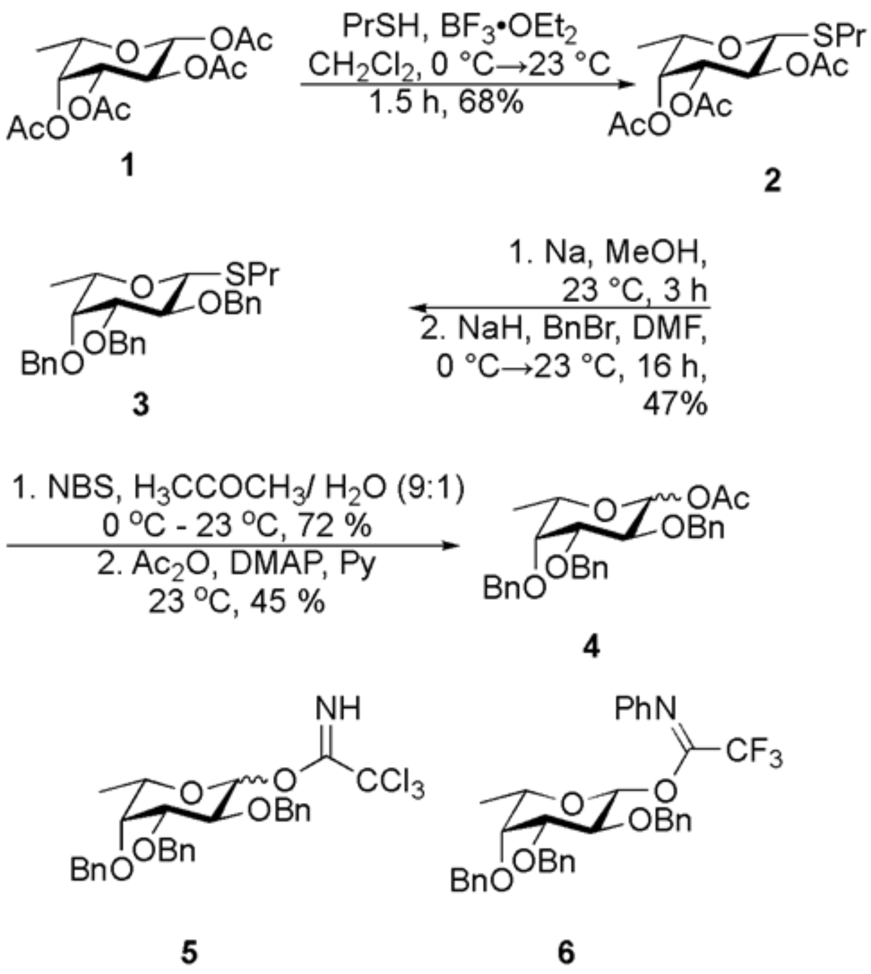

In construction of the fucose donor, propane thiol installation was performed on the known compound **1**, followed by deacylation under the Zemplén conditions and benzylation to produce compound **3**. The benzyl ether protected fucose thiol donor can also be used for glycosylation. Thiol donors wide range of stability under acidic and basic conditions make them ideal for synthesis along with their ability to be activated under various conditions.[81-85] Pentabenzylated fucose donors are known to be more stable under glycosylation conditions compared to other protected fucose derivatives. The acyl fucose donor **4**[86,87] was also prepared using NBS for thiol removal followed by acylation and known fucose donors **5** and **6** was prepared using known synthetic routes.[88,89]

Synthetic Procedures

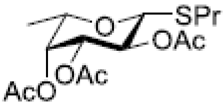

***n*-Propyl-2,3,4-tri-*O*-acyl-1-thio-β-L-fucopyranoside (2)**

Compound **1** (20.24 g, 60.91 mmol) was dissolved in anhydrous dichloromethane (50 mL) over an ice-water bath at 0 to 5 °C under argon atmosphere and stirred for 15 min. 1-Propanethiol (7.17 mL, 79.18 mmol, 1.30 equiv.) was added followed by the addition of boron trifluoride diethyletherate (21.92 mL, 85.27 mmol, 1.40 equiv.). The reaction mixture was stirred for 15 min then gradually raised to 23 °C and stirred for 3 h under argon. Upon completion, the reaction was quenched with excess triethylamine until the reaction solution was basic. The organic solution was concentrated via rotary evaporation followed by column purification via Teledyne ISCO CombiFlash® Rf 200i in hexane ethyl acetate step wise gradient with elution of compound in 15-20% ethyl acetate. The product fractions were collected, concentrated, and placed on high vacuum to give 14.37 g, 41.24 mmol, 68% as a white solid; R_f 0.28 (5:1 hexane: ethyl acetate).

^{1}H NMR (500MHz ,CHLOROFORM-d, 25 °C) δ 5.26 (dd, *J* = 1.0, 3.4 Hz, 1 H, H-4), 5.23 - 5.17 (m, 1 H, H-3), 5.03 (dd, *J* = 3.4, 9.8 Hz, 1 H, H-2), 4.43 (d, *J* = 9.8 Hz, 1 H, H-1), 3.81 (dd, *J* = 1.0, 6.3 Hz, 1 H, H-5), 2.73 - 2.59 (m, 2 H, $S\underline{CH_2}CH_2CH_3$), 2.16 (s, 3 H, CH_3C=O), 2.05 (s, 3 H, CH_3C=O), 1.97 (s, 3 H, CH_3C=O), 1.69 - 1.57 (m, 2 H,$SCH_2\underline{CH_2}CH_3$), 1.20 (d, *J* = 6.3 Hz, 3 H, H-6), 0.98 (t, *J* = 7.3 Hz, 3 H, $SCH_2CH_2\underline{CH_3}$);

^{13}C NMR (126 MHz, $CDCl_3$, 25 °C) δ 170.61, 170.09, 169.62, 83.72, 73.16, 72.36, 70.47, 67.42, 32.08, 22.99, 20.83, 20.68, 20.61, 16.40, 13.42;

HRMS (ESI): $[M + Na]^+$ *m/z* calc. for $C_{15}H_{24}O_7SNa^+$ 371.1140 found 371.1134.

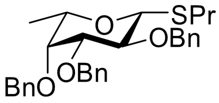

***n*-Propyl-2,3,4-tri-*O*-benzyl-1-thio-β-L-fucopyranoside (3)**

Compound **2** (14.37g, 41.24 mmol) was dissolved in methanol (415 mL) at 23 °C and stirred for 5 min. Sodium metal (0.4 g) was added and the reaction was stoppered with a septum containing an empty balloon and stirred for 4 h at 23 °C. Upon completion, the reaction was concentrated via rotary evaporation followed by co-evaporation with toluene (3x15mL). The crude product (10.58 g, 47.59 mmol) was dissolved in *N,N*-dimethylformamide (475 mL) over an ice-water bath at 0 to 5 °C under argon atmosphere and stirred for 15 min. Sodium hydride (9.52 g, 237.97 mmol, 5.0 equiv.) was added to the solution and the reaction was stirred for 15 min followed by the addition of benzyl bromide (28.30 mL, 237.97 mmol, 5.0 equiv). The reaction solution was stirred for an additional 15 min then allowed to rise to 23 °C and stirred for 13 h. Upon completion, the reaction mixture was quenched with methanol (~150 mL), diluted with dichloromethane (~200 mL) and washed with 2 M aqueous hydrochloric acid (~500 mL, 2x), saturated sodium bicarbonate (500 mL, 2x), and water (500 mL, 2x). The organic layer was dried over sodium sulfate, concentrated under reduced pressure and purified via Teledyne ISCO CombiFlash® Rf 200i. The product fractions were collected, concentrated, and placed on high vacuum to give 11.0 g, 22.33 mmol, 47% as a white solid; R_f 0.8 (1:1 hexane: ethyl acetate).

^{1}H NMR (500MHz ,CHLOROFORM-d, 25 °C) δ 7.53 - 7.32 (m, 15 H, Ar-H), 5.10 (d, *J* = 11.7 Hz, 1 H, $BnCH_2$), 5.01 (d, *J* = 10.3 Hz, 1 H, $BnCH_2$), 4.93 - 4.76 (m, 4 H, $BnCH_2$), 4.47 (d, *J* = 9.3 Hz, 1 H, H-1), 3.93 (t, *J* = 9.5 Hz, 1 H, H-2), 3.70 (d, *J* = 2.9 Hz, 1 H, H-3), 3.65 (dd, *J* = 2.9, 9.3 Hz, 1 H, H-4), 3.59 - 3.53 (m, 1 H, H-5), 2.88 - 2.71 (m, 2 H, $S\underline{CH_2}CH_2CH_3$), 1.84 - 1.71 (m, 2 H, $SCH_2\underline{CH_2}CH_3$), 1.31 (d, *J* = 6.3 Hz, 3 H, H-6), 1.09 (t, *J* = 7.3 Hz, 3 H, $SCH_2CH_2\underline{CH_3}$);

^{13}C NMR (126 MHz, $CDCl_3$, 25 °C) δ 138.82, 138.59, 138.54, 128.52, 128.38, 128.23, 127.76, 127.73, 127.63, 127.57, 85.28, 84.58, 78.50, 76.61, 75.77, 74.59, 72.95, 32.70, 23.36, 17.35, 13.72;

HRMS (ESI): $[M + Na]^+$ *m/z* calc. for $C_{30}H_{36}O_4SNa^+$ 515.2232 found 515.2209

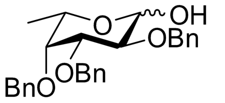

2,3,4-tri-*O*-benzyl-α-L-fucopyranoside

Compound **3** (1.215 g, 2.47 mmol) was dissolved in 9:1 acetone/water (25 mL) at 0 °C followed by the addition of *N*-bromosuccinimide (NBS) (1.76 g, 9.87 mmol, 4.0 equiv) and stirred until consumption of starting material via TLC, ~ 2 h. Upon completion the reaction was diluted with dichloromethane (25 mL) and washed with water (25 mL), sodium bicarbonate (25 mL), and brine (25 mL). The organic layer was dried over sodium sulfate, concentrated under reduced pressure and purified via Teledyne ISCO CombiFlash® Rf 200i in hexane ethyl acetate step wise gradient with elution of compound in 20% ethyl acetate. The product fractions were collected, concentrated, and placed on high vacuum to give 0.77 g, 1.77 mmol, 72% as a white solid; R_f 0.6 (1:1 hexane: ethyl acetate).

^{1}H NMR (500MHz ,CHLOROFORM-d) δ 7.45 - 7.29 (m, 16 H), 5.29 (d, *J* = 3.4 Hz, 1 H), 5.04 - 4.93 (m, 2 H), 4.88 - 4.63 (m, 6 H), 4.13 (q, *J* = 6.3 Hz, 1 H), 4.07 (dd, *J* = 3.7, 10.0 Hz, 1 H), 3.93 (dd, *J* = 2.7, 10.0 Hz, 1 H), 3.77 (dd, *J* = 7.6, 9.5 Hz, 1 H), 3.70 (d, *J* = 1.5 Hz, 1 H), 3.63 - 3.54 (m, 1 H), 1.23 (d, *J* = 6.3 Hz, 1 H), 1.17 (d, *J* = 6.3 Hz, 3 H);

^{13}C NMR (126MHz ,CHLOROFORM-d) δ 138.7, 138.5, 138.2, 128.4, 128.4, 128.4, 128.2, 128.2, 128.0, 127.9, 127.7, 127.6, 127.6, 127.6, 127.5, 97.7, 91.9, 82.6, 80.7, 79.1, 76.6, 76.4, 75.1, 74.8, 74.7, 73.6, 73.0, 70.8, 66.7, 35.3, 17.0, 16.8

Reference: [90]

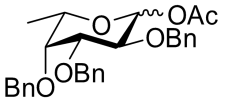

l-*O*-Acetyl-2,3,4-tri-O-benzyl-α-L-fucopyranose (4)

2,3,4-tri-*O*-benzyl-α-L-fucopyranoside (1.54 g, 3.54 mmol) was dissolved in anhydrous pyridine (35 mL) followed by the addition of acetic anhydride (0.67 mL, 7.09 mmol, 2.0 equiv) and 4-dimethylaminopyridine (DMAP) (216.5 mg, 1.77 mmol, 0.5 equiv) at 23 °C and stirred until consumption of starting material via TLC. The reaction solution was diluted with ethyl acetate (40 mL) and extraction was performed on the reaction mixture with water (40 mL). The organic layer was dried over sodium sulfate, concentrated under reduced pressure and purified via Teledyne ISCO CombiFlash® Rf 200i in hexane ethyl acetate step wise gradient with elution of compound in 10% ethyl acetate. The product fractions were collected, concentrated, and placed on high vacuum to give 0.76 g, 1.59 mmol, 45% as an oil; R_f 0.8 (1:1 hexane: ethyl acetate).

^{1}H NMR (500MHz ,CHLOROFORM-d) δ 7.44 - 7.29 (m, 15 H), 6.40 (d, *J* = 3.9 Hz, 1 H), 5.02 (d, *J* = 11.7 Hz, 1 H), 4.89 (d, *J* = 11.7 Hz, 1 H), 4.80 - 4.74 (m, 2 H), 4.72 (d, *J* = 6.3 Hz, 2 H), 4.68 (d, *J* = 11.7 Hz, 1 H), 4.19 (dd, *J* = 3.7, 10.0 Hz, 1 H), 4.03 - 3.97 (m, 1 H), 3.91 (dd, *J* = 2.9, 10.3 Hz, 1 H), 3.73 (d, *J* = 2.0 Hz, 1 H), 2.14 (s, 3 H), 1.17 (d, *J* = 6.3 Hz, 3 H);

^{13}C NMR (126MHz ,CHLOROFORM-d) δ 169.6, 138.7, 138.4, 138.1, 128.4, 128.4, 128.4, 128.2, 128.0, 127.8, 127.7, 127.7, 127.6, 127.6, 127.4, 90.9, 79.0, 75.0, 73.3, 73.2, 69.1, 21.2, 16.7

Reference: [91]

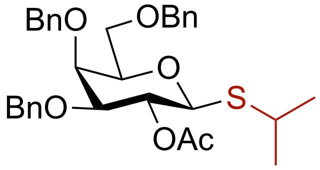

2-Propyl-2-acyl-3,4,6-tri-*O*-benzyl-1-thio-β-D-galactopyranoside (9)

Known compound **8** (5.0 g, 9.88 mmol) was dissolved in anhydrous dichloromethane (50 mL) over an ice-water bath at 0 to 5 °C under argon atmosphere and stirred for 15 min. 2-Propapnethiol (1.83 mL, 19.76 mmol, 2.0 equiv) was added followed by the addition of boron trifluoride diethyletherate (2.59 mL, 9.88 mmol, 1.0 equiv). The reaction mixture was stirred for 15 min then gradually raised to 23 °C and stirred for 1 h under argon. Upon completion, the reaction was quenched with excess triethylamine until the reaction solution was basic. The organic solution was concentrated via rotary evaporation followed by column purification via Teledyne ISCO CombiFlash® Rf 200i in hexane ethyl acetate step wise gradient with elution of compound in 10% ethyl acetate. The product fractions were collected, concentrated, and placed on high vacuum to give 4.88 g, 8.86 mmol, 89% as an oil; R_f 0.37 (4:1 hexane: ethyl acetate); ^{1}**H NMR** (500MHz ,CHLOROFORM-d) δ 7.41 - 7.25 (m, 15 H), 5.41 (t, *J* = 9.8 Hz, 1 H, C2), 4.97 (d, *J* = 11.7 Hz, 1 H, BnCH), 4.70 (d, *J* = 12.2 Hz, 1 H, BnCH), 4.65 - 4.53 (m, 2 H, $BnCH_2$), 4.50 - 4.41 (m, 3 H,$BnCH_2$,C1), 4.01 (d, *J* = 2.4 Hz, 1 H, C4), 3.66 - 3.54 (m, 4 H, C3, C5, C6), 3.20 (quin, *J* = 6.8 Hz, 1 H, $CH_3S\underline{CH}CH_3$), 2.05 (br. s., 3 H, OAc), 1.29 (dd, *J* = 6.8, 16.1 Hz, 6 H, $\underline{CH_3}SCH\underline{CH_3}$); ^{13}**C NMR** (126MHz ,CHLOROFORM-d) δ 169.6, 138.6, 138.0, 137.9, 128.4, 128.4, 128.4, 128.3, 128.3, 128.2, 128.1, 128.1, 128.1, 128.1, 127.9, 127.9, 127.8, 127.7, 127.5, 127.4, 83.7, 81.6, 74.4, 73.6, 72.9, 72.0, 70.0, 68.8, 34.8, 24.3, 23.7, 21.1;
HRMS (APCI): $[MH+]^+$ *m/z* calc. for $C_{32}H_{39}O_6SH^+$ 551.2462 found 551.2458; HSQC coupled 154 Hz meaning anomeric proton is axial.

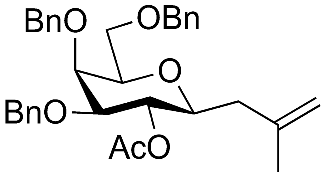

1-(2-methylallyl)-2-*O*-acetyl,3,4,6-tri-*O*-benzyl-β-D-galactopyranose (10)

Compound **8** (3.00 g, 5.92 mmol) was diluted in glacial acetic acid (10 mL) and stirred for 1 h at ambient temperature under argon. The reaction was then concentrated and coevaporated with toluene to give the crude product, which was then purified by MPLC (120 g silica, hexane to 1:1 hexane/ethyl acetate) to give compound **8A** (2.12 g, 67%).

^{1}**H NMR** (600 MHz, $CDCl_3$) δ 7.39 – 7.22 (m, 14H), 5.58 (dd, *J* = 8.2, 1.4 Hz, 1H), 5.48 (dd, *J* = 10.0, 8.2 Hz, 1H), 4.94 (d, *J* = 11.5 Hz, 1H), 4.67 (d, *J* = 12.1 Hz, 1H), 4.60 (d, *J* = 11.5 Hz, 1H), 4.51 (d, *J* = 12.2 Hz, 1H), 4.43 (q, *J* = 11.7 Hz, 2H), 4.12 (q, *J* = 7.1 Hz, 1H), 4.01 (dd, *J* = 2.9, 1.2 Hz, 1H), 3.74 – 3.68 (m, 1H), 3.67 – 3.55 (m, 3H), 2.05 (d, *J* = 8.8 Hz, 4H), 2.00 (d, *J* = 1.4 Hz, 3H).

Compound **8A** (2.12 g, 3.97 mmol) was dissolved in anhydrous dichloromethane (46.1 mL, 86.0 mM) and was cooled to -20 °C. Methylallyltrimethylsilane (2.79 mL, 15.9 mmol) was added, followed by boron trifluoride diethyletherate (1.25 mL, 10.2 mmol). The reaction was stirred overnight under argon and upon completion was poured into saturated bicarbonate solution (100 mL) and was extracted with dichloromethane (3 x 100 mL). The organic layers were collected and dried with magnesium sulfate and solvent was removed under reduced pressure. The crude material was then purified via MPLC (120 g silica, hexane to 20% ethyl acetate in hexane) to give the purified product **10** (1.1172 g, 53%).

^{1}**H NMR** (500 MHz, $CDCl_3$) δ 7.34 – 7.16 (m, 15H), 5.23 (t, *J* = 9.6 Hz, 1H), 4.88 (d, *J* = 11.7 Hz, 1H), 4.71 – 4.66 (m, 2H), 4.63 (d, *J* = 12.2 Hz, 1H), 4.55 (d, *J* = 11.7 Hz, 1H), 4.49 – 4.39 (m, 2H), 4.36 (d, *J* = 11.7 Hz, 1H), 3.93 (d, *J* = 2.8 Hz, 1H), 3.56 – 3.43 (m, 4H), 3.39 (td, *J* = 9.0, 3.6 Hz, 1H), 2.32 – 2.05 (m, 2H), 1.94 (s, 3H), 1.68 (s, 3H).

^{13}C NMR (126 MHz, $CDCl_3$) δ 170.13, 142.38, 138.72, 138.31, 138.20, 128.59, 128.48, 128.41, 128.39, 128.07, 127.93, 127.87, 127.75, 127.62, 112.37, 81.97, 77.61, 77.45, 77.40, 77.34, 77.20, 76.94, 74.56, 73.72, 73.23, 72.29, 71.94, 69.23, 40.26, 29.88, 23.00, 21.27.

HRMS (ESI) m/z $[M+Na]^+$ Calcd for $C_{33}H_{38}O_6Na$ 553.2561; Found 553.2560, $[M+O+Na]^+$ Calcd for $C_{33}H_{38}O_7Na$ 569.2510; Found 569.2508.

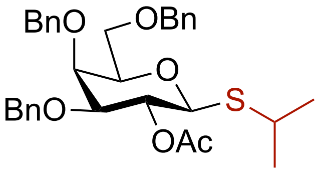

2-Propyl-3,4,6-tri-*O*-benzyl-1-thio-β-D-galactopyranoside (11)

Compound **9** (0.86 g, 1.56 mmol) was dissolved in anhydrous methanol (100 mL) at 23 °C followed by the addition of sodium metal (~0.3 g). The reaction mixture was stirred for 7 h and upon completion the reaction was quenched with DOWEX H^+ resin until pH was acidic. The organic solution was filtered and concentrated via rotary evaporation followed by column purification in chloroform/ethanol. The product fractions were collected, concentrated, and placed on high vacuum to give 0.72 g, 1.42 mmol, 91% as an oil; R_f 0.62 (30:1 chloroform: ethanol);

^{1}H NMR (500MHz ,CHLOROFORM-d) δ 7.43 - 7.24 (m, 15 H), 4.92 (d, *J* = 11.7 Hz, 1 H), 4.80 - 4.70 (m, 2 H), 4.62 (d, *J* = 11.7 Hz, 1 H), 4.52 - 4.43 (m, 2 H), 4.41 (d, *J* = 9.3 Hz, 1 H), 4.02 - 3.94 (m, 2 H), 3.67 - 3.60 (m, 3 H), 3.48 (dd, *J* = 2.4, 9.3 Hz, 1 H), 3.22 (td, *J* = 6.8, 13.3 Hz, 1 H), 2.45 (d, *J* = 1.5 Hz, 1 H), 1.34 (d, *J* = 6.8 Hz, 6 H);

^{13}C NMR (126MHz ,CHLOROFORM-d) δ 138.6, 128.5, 128.4, 128.2, 128.1, 127.9, 127.8, 127.8, 127.6, 127.5, 86.2, 83.3, 77.6, 74.5, 73.6, 73.3, 72.5, 70.0, 68.9, 35.3, 24.1;

HRMS (ESI): $[M + Na]^+$ *m/z* calc. for $C_{30}H_{36}O_5SNa^+$ 531.2176 found 531.2174; HSQC decoupled 155.5 Hz meaning anomeric proton is axial.

Deacetylation under Zemplén Conditions using continuous-flow process:

Compound **9** (0.0478 g, 0.08679 mmol) was dissolved in anhydrous methanol (2 mL) and sodium methoxide 25 wt% in MeOH (2.5 mL) was streamed together via HPLC pump at a flowrate of 0.1 mL/min through high purity PFA tubing [0.02 I.D., 1524 cm (50 ft), 3 mL) with a residence time of 30 min at 23 °C. Additional methanol was passed through the flow system to collect the remaining reaction solution (8 mL). The reaction solution was collected in a vial containing DOWEX H^+ resin (7.0 g), filtered and purified via Teledyne ISCO CombiFlash® Rf 200i in hexane/ethyl acetate in a stepwise gradient with elution of compound in 20% ethyl acetate to provide 41.2 mg, 0.081 mmol, 93%

MechWolf Code:

```
import mechwolf as mw

# create components
vessel_1 = mw.Vessel("47.8 mg of 2-OAc, 3,4,6-OBn IPTG in 2 mL MeOH", name='47.8 mg of2-OAc, 3,4,6-OBn IPTG in 2 mL MeOH')
vessel_2 = mw.Vessel("2.5 mL of 25 wt% NaOMe", name='2.5 mL of 25 wt% NaOMe')
out = mw.Vessel("quench 8.2 g 50WX8 Hydrogen Form 200 to 400 mesh DOWEX", name='quench 8.2 g 50WX8 Hydrogen Form 200 to 400 mesh DOWEX')

thick_tube = mw.Tube(length="1.375 feet", ID="0.062 in", OD="1/8 in", material="PFA")
```

```
rxn_tube = mw.Tube(length="50 feet", ID="0.02 in", OD="1/16 in", material="reaction zone PFA")

thin_tube = mw.Tube(length="1.68 feet", ID="0.02 in", OD="1/16 in", material="PFA")

pump0 =mw.VarianPump(name="pump0")
pump1 =mw.VarianPump(name="pump1")

mixer = mw.TMixer()

# create apparatus
A = mw.Apparatus("Deacylation2")

A.add(vessel_1, pump0, thick_tube)
A.add(vessel_2, pump1, thick_tube)

A.add([pump0,pump1],mixer,thin_tube)

A.add(mixer, out, rxn_tube)

A.describe()
A.visualize()

# create protocol
P = mw.Protocol(A, name="Deacylation")
P = mw.Protocol(A, duration="auto")

P.add(pump0, start="0 secs", stop="190 min", rate="0.05 mL/min")
P.add(pump1, start="0 secs", stop="190 min", rate="0.05 mL/min")


#P.visualize()

#print(P.yaml())

P.execute(hub_id='kee', security_key='easiness-wilder-observant-urethane-embody-contour')
```

Deacetylation under Zemplén Conditions using continuous-flow process:

Compound **9** (0.0484 g,0.08788 mmol) was dissolved in anhydrous methanol (2 mL) and sodium methoxide 25 wt% in MeOH (2.5 mL) was streamed together via HPLC pump at a flowrate of 0.2 mL/min through high purity PFA tubing [0.02 I.D., 1524 cm (50 ft), 3 mL) with a residence time of 15 min at 23 °C. Additional methanol was passed through the flow system to collect the remaining reaction solution (8 mL). The reaction solution was collected in a vial containing DOWEX H^+ resin (7.0 g), filtered and purified via Teledyne ISCO CombiFlash® Rf 200i in hexane/ethyl acetate in a stepwise gradient with elution of compound in 20 % ethyl acetate to provide 30.4 mg, 0.05976 mmol, 68%

MechWolf Code:

```
import mechwolf as mw

# create components
vessel_1 = mw.Vessel("48.4 mg of 2-OAc, 3,4,6-OBn IPTG in 2 mL MeOH", name='48.4 mg of2-OAc, 3,4,6-OBn IPTG in 2 mL MeOH')
vessel_2 = mw.Vessel("2.5 mL of 25 wt% NaOMe", name='2.5 mL of 25 wt% NaOMe')
out = mw.Vessel("quench 7.0 g 50WX8 Hydrogen Form 200 to 400 mesh DOWEX", name='quench 7.0 g 50WX8 Hydrogen Form 200 to 400 mesh DOWEX')

thick_tube = mw.Tube(length="1.375 feet", ID="0.062 in", OD="1/8 in", material="PFA")

rxn_tube = mw.Tube(length="50 feet", ID="0.02 in", OD="1/16 in", material="reaction zone PFA")

thin_tube = mw.Tube(length="1.68 feet", ID="0.02 in", OD="1/16 in", material="PFA")

pump0 =mw.VarianPump(name="pump0")
pump1 =mw.VarianPump(name="pump1")

mixer = mw.TMixer()

# create apparatus
A = mw.Apparatus("Deacylation2")

A.add(vessel_1, pump0, thick_tube)
A.add(vessel_2, pump1, thick_tube)

A.add([pump0,pump1],mixer,thin_tube)

A.add(mixer, out, rxn_tube)

A.describe()
A.visualize()

# create protocol
P = mw.Protocol(A, name="Deacylation")
P = mw.Protocol(A, duration="auto")

P.add(pump0, start="0 secs", stop="120 min", rate="0.1 mL/min")
P.add(pump1, start="0 secs", stop="120 min", rate="0.1 mL/min")


#P.visualize()

#print(P.yaml())

P.execute(hub_id='kee', security_key='easiness-wilder-observant-urethane-embody-contour')
```

Deacetylation under Zemplén Conditions using continuous-flow process:
Compound **9** (0.0481 g, 0.08734 mmol) was dissolved in anhydrous methanol (2 mL) and sodium methoxide 25 wt% in MeOH (2.5 mL) was streamed together via HPLC pump at a flowrate of 0.4 mL/min through high purity PFA tubing [0.02 I.D., 1524 cm (50 ft), 3 mL) with a residence time of 7.5 min at 23 °C. Additional methanol was passed through the flow system to collect the remaining reaction solution (8 mL). The reaction solution was collected in a vial containing DOWEX H^+ resin (7.0 g), filtered and purified via Teledyne ISCO CombiFlash® Rf 200i in hexane/ethyl acetate in a stepwise gradient with elution of compound in 20% ethyl acetate to provide 21.5 mg, 0.04227 mmol, 48.4 %

MechWolf Code:

```
import mechwolf as mw

# create components
vessel_1 = mw.Vessel("48.1 mg of 2-OAc, 3,4,6-OBn IPTG in 2 mL MeOH", name='48.1 mg of2-OAc, 3,4,6-OBn IPTG in 2 mL MeOH')
vessel_2 = mw.Vessel("2.5 mL of 25 wt% NaOMe", name='2.5 mL of 25 wt% NaOMe')
out = mw.Vessel("quench 7.0 g 50WX8 Hydrogen Form 200 to 400 mesh DOWEX", name='quench 7.0 g 50WX8 Hydrogen Form 200 to 400 mesh DOWEX')

thick_tube = mw.Tube(length="1.375 feet", ID="0.062 in", OD="1/8 in", material="PFA")

rxn_tube = mw.Tube(length="50 feet", ID="0.02 in", OD="1/16 in", material="reaction zone PFA")

thin_tube = mw.Tube(length="1.68 feet", ID="0.02 in", OD="1/16 in", material="PFA")

pump0 =mw.VarianPump(name="pump0")
pump1 =mw.VarianPump(name="pump1")

mixer = mw.TMixer()

# create apparatus
A = mw.Apparatus("Deacylation2")

A.add(vessel_1, pump0, thick_tube)
A.add(vessel_2, pump1, thick_tube)

A.add([pump0,pump1],mixer,thin_tube)

A.add(mixer, out, rxn_tube)

A.describe()
A.visualize()

# create protocol
P = mw.Protocol(A, name="Deacylation")
P = mw.Protocol(A, duration="auto")
```

```
P.add(pump0, start="0 secs", stop="48 min", rate="0.2 mL/min")
P.add(pump1, start="0 secs", stop="48 min", rate="0.2 mL/min")


#P.visualize()

#print(P.yaml())

P.execute(hub_id='kee', security_key='easiness-wilder-observant-urethane-embody-contour')
```

Deacetylation under Zemplén Conditions using continuous-flow process with Static Mixer StaMixCo:

Compound **9** (0.0487 g, 0.08843 mmol) was dissolved in anhydrous methanol (2 mL) and sodium methoxide 25 wt% in MeOH (2.5 mL) was streamed together via HPLC pump at a flowrate of 0.4 mL/min through high purity PFA tubing [0.02 I.D., 1524 cm (50 ft), 3 mL) with a residence time of 7.5 min at 23 °C containing a StaMixCo Model HT-40-1.70-10-PTFE (3 cm, 1.7 mm O.D.) static mixer placed inside 6 cm 0.062 I.D. x 1/8 O.D. PFA tubing. Additional methanol was passed through the flow system to collect the remaining reaction solution (8 mL). The reaction solution was collected in a vial containing DOWEX H^+ resin (8.2 g), filtered and purified via Teledyne ISCO CombiFlash® Rf 200i in hexane/ethyl acetate in a stepwise gradient with elution of compound in 20% ethyl acetate to provide 34.4 mg, 0.06763 mmol, 76.5 %

MechWolf Code:

```
import mechwolf as mw

# create components
vessel_1 = mw.Vessel("48.7 mg of 2-OAc, 3,4,6-OBn IPTG in 2 mL MeOH", name='48.7 mg of2-OAc, 3,4,6-OBn IPTG in 2 mL MeOH')
vessel_2 = mw.Vessel("2.5 mL of 25 wt% NaOMe", name='2.5 mL of 25 wt% NaOMe')
out = mw.Vessel("quench 7.0 g 50WX8 Hydrogen Form 200 to 400 mesh DOWEX", name='quench 7.0 g 50WX8 Hydrogen Form 200 to 400 mesh DOWEX')

thick_tube = mw.Tube(length="1.375 feet", ID="0.062 in", OD="1/8 in", material="PFA")

rxn_tube = mw.Tube(length="50 feet", ID="0.02 in", OD="1/16 in", material="reaction zone PFA")

thin_tube = mw.Tube(length="1.68 feet", ID="0.02 in", OD="1/16 in", material="PFA")

pump0 =mw.VarianPump(name="pump0")
pump1 =mw.VarianPump(name="pump1")

mixer = mw.TMixer()

# create apparatus
A = mw.Apparatus("Deacylation2")

A.add(vessel_1, pump0, thick_tube)
A.add(vessel_2, pump1, thick_tube)
```

```
A.add([pump0,pump1],mixer,thin_tube)

A.add(mixer, out, rxn_tube)

A.describe()
A.visualize()

# create protocol
P = mw.Protocol(A, name="Deacylation")
P = mw.Protocol(A, duration="auto")

P.add(pump0, start="0 secs", stop="48 min", rate="0.2 mL/min")
P.add(pump1, start="0 secs", stop="48 min", rate="0.2 mL/min")



#P.visualize()

#print(P.yaml())

P.execute(hub_id='kee', security_key='easiness-wilder-observant-urethane-embody-contour')
```

Discussion of deacetylation optimization

We first sought to investigate the deprotection of compound **9** under Zemplén conditions. Noticing the viscous nature of 25 wt% NaOMe, it seemed likely that faster flow rates could result in better reaction performance due to an increased turbulence resulting in adequate mixing. Reactions were performed under various flow rates to determine minimal residence time for full conversion, and faster flow rates gave poor reaction performance, resulting in incomplete consumption of compound **9**. This evidence suggested that an increase in residence time (decreased flow rate) was needed for full conversion (Figure 5 SI, 0.1 mL/min, 30 min). When a PTFE helical static mixer (HT-40-1.70-10-PTFE) from StaMixCo LLC was incorporated, better reaction performance was observed at a high flow rate (76.5% with static mixer vs 48.4% without static mixer at 0.4 mL/min), showcasing the importance of adequate mixing.

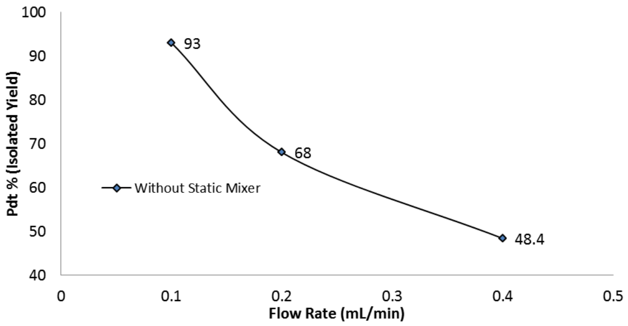

Figure 5 SI. Deacetylation under Zemplén conditions continuous flow process without static mixer.

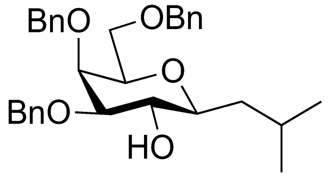

1-(2-isobutyl)- 3,4,6-tri-*O*-benzyl-β-D-galactopyranose (12)

Compound **10** (500 mg, 942 µmol) and hydrazine monohydrate (3.89 mL, 80.1 mmol) were dissolved in ethanol (19.4 mL, 48.6 mM) and aqueous hydrogen peroxide (30% in water, 1.44 mL, 14.1 mmol) was added slowly over the course of 3 h. The reaction was stirred at ambient temperature for 18 h and was then quenched with saturated sodium thiosulfate (2.1 mL) under ice cooling and was stirred for 30 min. This was then diluted in ethyl acetate and washed with water, sodium thiosulfate solution, and brine. The organic layer was collected and dried with magnesium sulfate, filtered, and concentrated under reduced pressure. The crude material was purified via MPLC (12 g silica, hexane to 15% ethyl acetate in hexane) to give compound **12** (185.0 mg, 40%).

^{1}H NMR (500 MHz, $CDCl_3$) δ 7.46 – 7.19 (m, 24H), 4.83 (d, *J* = 11.7 Hz, 1H), 4.72 (d, *J* = 11.7 Hz, 1H), 4.63 (d, *J* = 11.8 Hz, 1H), 4.51 – 4.41 (m, 3H), 4.02 (d, *J* = 2.7 Hz, 1H), 3.72 (d, *J* = 9.3 Hz, 1H), 3.65 – 3.54 (m, 2H), 3.37 (dd, *J* = 9.3, 2.7 Hz, 1H), 3.23 (td, *J* = 9.7, 2.4 Hz, 1H), 1.88 (dtd, *J* = 16.3, 6.6, 3.3 Hz, 1H), 1.60 (ddd, *J* = 14.2, 9.8, 2.4 Hz, 1H), 1.49 (ddd, *J* = 14.2, 10.0, 4.5 Hz, 1H), 1.01 – 0.81 (m, 11H).

^{13}C NMR (126 MHz, $CDCl_3$) δ 138.75, 138.17, 138.01, 128.93, 128.87, 128.83, 128.69, 128.66, 128.62, 128.50, 128.42, 128.37, 128.19, 128.14, 128.10, 127.98, 127.95, 127.81, 84.61, 78.42, 77.46, 77.41, 77.20, 76.95, 74.56, 73.76, 72.69, 71.78, 71.24, 69.20, 40.93, 24.40, 23.91, 21.75.

HRMS (ESI) m/z: $[M+Na]^+$ Calcd for $C_{31}H_{38}O_5Na$ 513.2611; Found 513.2612

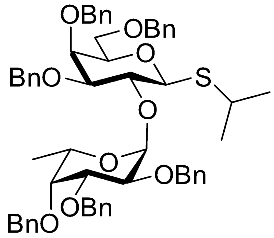

2-Propyl thio 2,3,4-tri-*O*-benzyl-alpha-L-fuco-pyranosyl-(1-2)- 3,4,6-tri-*O*-benzyl-beta-D-galactopyranoside (13)

Compound **11** (265.0 mg, 0.52096 mmol) and **4** (496.55 mg, 1.04 mmol) was dissolved in CH_2Cl_2 (5.3 mL) at 0 °C with stirring. $BF_3{\cdot}OEt_2$ (6.43 mmL, 0.05210 mmol) was added and the reaction solution was stirred until consumption of compound **11**. The reaction mixture was quenched with triethylamine, concentrated via rotary evaporation followed by column chromatography Teledyne ISCO CombiFlash® Rf 200i in hexane ethyl acetate step wise gradient with elution of compound in 10% ethyl acetate. Additional column using 5:1 Pentane/Ether was also performed. The product fractions were collected, concentrated, and placed on high vacuum to give 38.8 mg, 0.04194 mmol, 8% as an oil; R_f 0.46 (5:1 Hexane/ethyl acetate);

^{1}H NMR (500MHz ,CHLOROFORM-d) δ 7.45 - 7.22 (m, 28 H), 7.20 - 7.14 (m, 3 H), 5.55 (d, *J* = 5.4 Hz, 1 H), 5.12 (d, *J* = 3.4 Hz, 1 H), 5.00 (d, *J* = 11.2 Hz, 2 H), 4.97 - 4.90 (m, 2 H), 4.84 - 4.70 (m, 2 H), 4.70 - 4.56 (m, 4 H), 4.52 - 4.29 (m, 5 H), 4.06 (dd, *J* = 3.9, 10.3 Hz, 1 H), 4.03 - 3.98 (m, 1 H), 3.94 - 3.88 (m, 1 H), 3.81 (dd, *J* = 2.9, 9.8 Hz, 1 H), 3.72 - 3.67 (m, 1 H), 3.67 - 3.60 (m, 1 H), 3.59 - 3.52 (m, 2 H), 3.03 - 2.93 (m, 1 H), 1.27 (dd, *J* = 1.2, 6.6 Hz, 6 H), 1.13 (d, *J* = 6.3 Hz, 3 H);

^{13}C NMR (126MHz ,CHLOROFORM-d) δ 139.3, 139.1, 138.7, 138.1, 128.4, 128.4, 128.4, 128.3, 128.3, 128.3, 128.3, 128.2, 128.2, 128.2, 128.1, 128.1, 128.1, 127.9, 127.9, 127.9, 127.9, 127.7, 127.6, 127.6, 127.6, 127.6, 127.5, 127.4, 127.4, 127.4, 127.2, 127.1, 100.9, 83.6, 80.5, 79.3, 79.2, 78.4, 78.0, 77.8, 77.2, 76.1, 75.7, 74.8, 74.6, 73.4, 73.3, 73.2, 72.6, 69.7, 69.1, 67.6, 33.1, 23.6, 23.3, 16.6;

HRMS (ESI): [M + Na]$^+$ *m/z* calc. for $C_{57}H_{64}O_9SNa^+$ 947.4166 found 947.4163

Batch Conditions:

Entry	Solvent	Concentration (mmol/mL)*	Fucose Donor	Promoter	Promoter Equiv	Temperature (°C)	Yield (%)
1	CH_2Cl_2	0.009	N-Phenyl trifluoroacetimidate	TMSOTf	0.2	-40	20
2	CH_2Cl_2	0.05	N-Phenyl trifluoroacetimidate	TMSOTf	0.2	-40	25.7
3	Toluene	0.05	N-Phenyl trifluoroacetimidate	TMSOTf	0.2	-40	40.7
4	CH_2Cl_2	0.09	acyl	$BF_3{\cdot}OEt_2$	0.1	0	8

*Concentration relative to acceptor

Continuous-flow process procedure:

Compound **11** (0.025 g, 0.04915 mmol) and **6** (0.08930 g, 0.14744 mmol) was together dissolved in anhydrous toluene (0.5 mL) and TMSOTf (1 mL of 0.02 M) was streamed together via HPLC pump at a flowrate of 0.4 mL/min through high purity PFA tubing (0.02 I.D., 1524 cm (50 ft), 3 mL) with a residence time of 7.5 min at -40 °C. Additional toluene was passed through the flow system to collect the remaining reaction solution (8 mL). The reaction solution was collected in test tubes containing 0.05 mL of Et_3N and reaction solution at steady state was collected, concentrated, and purified a via column chromatography in hexane/ethyl acetate in a stepwise gradient with elution of compound in 16% ethyl acetate to provide 11.5 mg, 0.01243 mmol, 25%

Continuous-flow Conditions:

Entry	Solvent	Concentration (mmol/mL)*	Fucose Donor	Promoter	Temp (°C)	Flow rate (mL/min)	Residence time (min)	Yield (%)
1	Toluene	0.04	N-Phenyl trifluoroacetimidate	TMSOTf	-40	0.1	30	degrade
2	Toluene	0.04	N-Phenyl trifluoroacetimidate	TMSOTf	-40	0.4	7.5	25

*Concentration relative to acceptor in the reactor tube.

MechWolf Code:

```
import mechwolf as mw

# create components
vessel_1 = mw.Vessel("25 mg of IPTG acceptor and 89.3 mg Fucose donor in 0.5 mL toluene", name='25 mg of IPTG acceptor and 89.3 mg Fucose donor in 0.5 mL toluene')
vessel_2 = mw.Vessel("0.02 mM of TMSOTf in 1.0 mL toluene", name='0.02 mM of TMSOTf in 1.0 mL toluene')
out = mw.Vessel("quench 0.05 mL Et3N in Test tubes", name='quench 0.05 mL Et3N in Test tubes')

thick_tube = mw.Tube(length="1.375 feet", ID="0.062 in", OD="1/8 in", material="PFA")

rxn_tube = mw.Tube(length="50 feet", ID="0.02 in", OD="1/16 in", material="reaction zone PFA")

thin_tube = mw.Tube(length="1.68 feet", ID="0.02 in", OD="1/16 in", material="PFA")

pump0 =mw.VarianPump(name="pump0")
pump1 =mw.VarianPump(name="pump1")

mixer = mw.TMixer()

# create apparatus
A = mw.Apparatus("Deacylation2")

A.add(vessel_1, pump0, thick_tube)
A.add(vessel_2, pump1, thick_tube)

A.add([pump0,pump1],mixer,thin_tube)

A.add(mixer, out, rxn_tube)

A.describe()
A.visualize()

# create protocol
P = mw.Protocol(A, name="Deacylation")
P = mw.Protocol(A, duration="auto")

P.add(pump0, start="0 secs", stop="60 min", rate="0.2 mL/min")
P.add(pump1, start="0 secs", stop="60 min", rate="0.2 mL/min")
#P.visualize()

#print(P.yaml())

P.execute(hub_id='kee', security_key='easiness-wilder-observant-urethane-embody-contour')
```

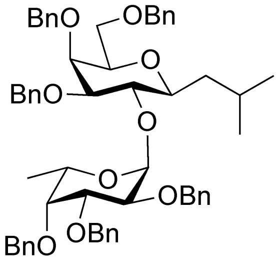

1-isobutyl-2,3,4-tri-*O*-benzyl-α-L-fucopyranose-(1-2)-3,4,6-tri-*O*-benzyl-β-D-galactopyranose (15α)

Compound **12** (50.0 mg, 101 µmol) and compound **6** (226 mg, 373 µmol). Compound **12** and compound **6** were coevaporated with toluene separately and were dried under high vacuum overnight. Compounds **12** and **6** were then combined into a vial and were dissolved in toluene (0.8 mL). A TMSOTf solution was prepared in a separate vial (10 µL of TMSOTf in 2.8 mL of toluene). These two solutions were then loaded into separate syringes. The syringes were connected to PFA tubing (I. D. 0.02", 1 ft), and the tubing was connected to a tee assembly (0.02"). From the tee assembly, the reactor tubing was connected (I. D. 0.02", 50 ft). Desired flow rate, run time, and reaction parameters were entered into Jupyter notebook, and this information was used to start the pumps at a flow rate of 0.2 mL/min (giving an overall flow rate of 0.4 mL/min). The donor and acceptor solution was flowed with the TMSOTf solution at a flow rate of 0.4 mL/min through PFA tubing with a residence time of 7.5 min. The reaction solution was collected at steady state, and the volume of the reaction solution was determined. The reaction solution was then concentrated in vacuo and purified using semipreparative HPLC on a Phenomenex C-18 column (100 Å, 5 µm, 250 x 10 mm) using the method in the table below:

A = water, B = acetonitrile

Time (minutes: seconds)	%A	%B
0:00	90	10
2:00	90	10
7:30	25	75
12:30	15	85
15:00	0	100
25:00	0	100

Flow rate = 4.0 mL/min, column temperature = 45 °C, products eluted at 21 min and 23 min

Two anomerically pure disaccharides were isolated from the purification: **15α** (16.2 mg, 25%) and **15β** (9.8 mg, 15%).
^{1}H NMR (600 MHz, $CDCl_3$) δ 7.43 – 7.13 (m, 32H), 5.90 (d, *J* = 4.1 Hz, 1H), 4.97 (d, *J* = 11.6 Hz, 1H), 4.90 – 4.73 (m, 5H), 4.68 (d, *J* = 11.6 Hz, 1H), 4.60 – 4.50 (m, 6H), 4.48 (d, *J* = 11.7 Hz, 1H), 4.12 – 4.00 (m, 4H), 3.93 (dd, *J* = 10.4, 2.7 Hz, 1H), 3.78 (dd, *J* = 9.1, 3.0 Hz, 1H), 3.75 – 3.70 (m, 1H), 3.62 (d, *J* = 6.3 Hz, 2H), 3.58 (d, *J* = 6.3 Hz, 1H), 3.49 – 3.40 (m, 1H), 3.19 (qd, *J* = 7.3, 4.9 Hz, 1H), 2.00 – 1.91 (m, 1H), 1.56 (dt, *J* = 9.5, 4.6 Hz, 2H), 1.44 (t, *J* = 7.3 Hz, 1H), 1.18 (d, *J* = 6.5 Hz, 3H), 0.93 (ddd, *J* = 16.0, 14.7, 6.6 Hz, 7H).
HRMS (ESI) m/z $[M+Na]^+$ Calcd for $C_{58}H_{66}O_9Na$ 929.4599; Found 929.4605

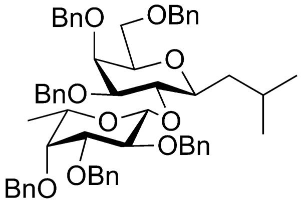

1-isobutyl-2,3,4-tri-*O*-benzyl-β-L-fucopyranose-(1-2)-3,4,6-tri-*O*-benzyl-β-D-galactopyranose (15β)

Compound **12** (50.0 mg, 102 μmol) and compound **6** (226 mg, 373 μmol) were coevaporated with toluene separately and were dried under high vacuum overnight. Compounds **12** and **6** were then combined into a vial and were dissolved in toluene (0.8 mL). A TMSOTf solution was prepared in a separate vial (10 μL of TMSOTf in 2.8 mL of toluene). These donor and acceptor solution (50.0 mg compound **12**, 226 mg compound **6**, 1.0 mL toluene) and TMSOTf solution (3.7 μL in 1000 μL of toluene) were then loaded into two separate syringes. The syringes were connected to PFA tubing (I. D. 0.02", 1 ft), and the tubing was connected to a tee assembly (0.02"). From the tee assembly, the reactor tubing was connected (I. D. 0.02", 50 ft). Desired flow rate, run time, and reaction parameters were entered into Jupyter notebook, and this information was used to start the pumps at a flow rate of 0.4 mL/min. The donor and acceptor solution was flowed with the TMSOTf solution at a flow rate of 0.4 mL/min through PFA tubing with a residence time of 7.5 min. The reaction solution was collected at steady state, and the volume of the reaction solution was determined. The reaction solution was then concentrated in vacuo and purified using semipreparative HPLC on a Phenomenex C-18 column (100 Å, 5 μm, 250 x 10 mm). Two anomerically pure disaccharides were isolated from the purification: **15α** (16.2 mg, 25%) and **15β** (9.8 mg, 15%).

^{1}H NMR (600 MHz,$CDCl_3$) δ 7.42 – 7.36 (m, 2H), 7.36 – 7.17 (m, 28H), 4.95 (d, *J* = 11.7 Hz, 1H), 4.91 (d, *J* = 11.2 Hz, 1H), 4.89 – 4.81 (m, 3H), 4.76 – 4.65 (m, 5H), 4.62 (d, *J* = 11.7 Hz, 1H), 4.55 (d, *J* = 11.5 Hz, 1H), 4.48 (d, *J* = 11.7 Hz, 1H), 4.41 (d, *J* = 11.7 Hz, 1H), 4.00 – 3.94 (m, 2H), 3.75 (dd, *J* = 9.7, 7.6 Hz, 1H), 3.63 – 3.48 (m, 6H), 3.33 (ddd, *J* = 11.1, 9.2, 2.1 Hz, 1H), 3.22 – 3.11 (m, 2H), 1.83 (ddt, *J* = 13.5, 6.6, 3.8 Hz, 1H), 1.75 (ddd, *J* = 12.8, 10.5, 2.1 Hz, 1H), 1.50 (ddd, *J* = 14.0, 10.7, 3.7 Hz, 1H), 1.40 (t, *J* = 7.3 Hz, 2H), 1.00 (d, *J* = 6.3 Hz, 3H), 0.75 (dd, *J* = 6.6, 5.0 Hz, 5H).

HRMS (ESI) m/z $[M+Na]^+$ Calcd for $C_{58}H_{66}O_9Na$ 929.4599; Found 929.4608

Scheme 2 SI. Synthesis of n-Propyl-6,4-benzylidene-2,3-di-O-benzyl-1-thio-β-D-galactopyranoside.

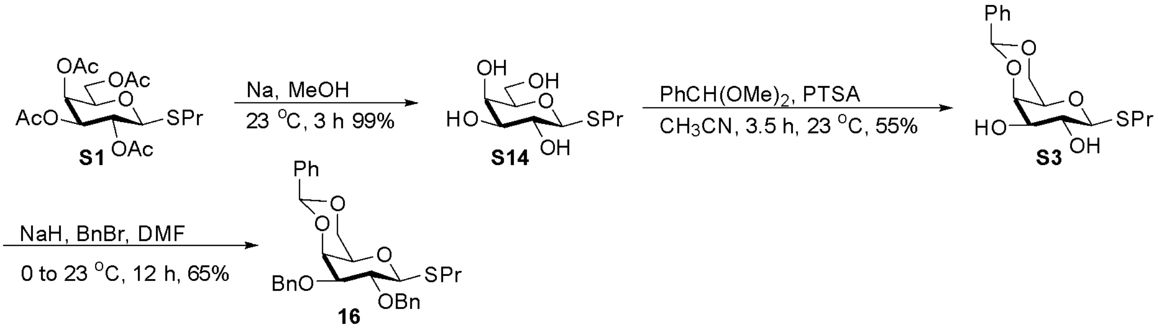

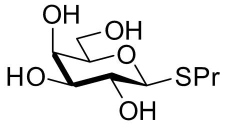

n-Propyl-1-thio-β-D-galactopyranoside (S**14**)

Compound **S1** (7.24 g, 17.81 mmol) was dissolved in anhydrous methanol (50 mL) at 23 °C. Sodium metal (~0.3 g) was added and the mixture was allowed to mix at 23 °C under atmosphere. Once the reaction was complete, the reaction mixture was quenched with DOWEX H^+ resin until the pH was neutral. The reaction mixture was filtered, coevaoporated with Toluene and dried on high vacuum to give 4.19 g, 17.62 mmol, 99%.^{1}**H NMR** (500MHz ,DMSO-d$_6$) δ 4.88 (d, *J* = 5.4 Hz, 1 H), 4.75 (d, *J* = 5.4 Hz, 1 H), 4.54 (t, *J* = 5.6 Hz,

1 H), 4.36 (d, J = 3.9 Hz, 1 H), 4.18 (d, J = 9.8 Hz, 1 H), 3.70 - 3.66 (m, 1 H), 3.53 - 3.42 (m, 2 H), 3.37 - 3.30 (m, 2 H), 3.29 - 3.24 (m, 1 H), 2.66 - 2.52 (m, 2 H), 1.61 - 1.51 (m, 2 H), 0.93 (t, J = 7.3 Hz, 3 H);

^{13}C NMR (126MHz ,DMSO-d$_6$) δ 86.1, 79.6, 75.2, 70.3, 68.8, 61.0, 31.5, 23.3, 13.9;

HRMS (ESI): [M + Na]$^+$ *m/z* calc. for $C_9H_{18}O_5SNa^+$ 261.0773 found 261.0761

*Batch process for the construction of **16**

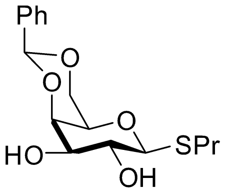

n-Propyl-6,4-benzylidene-1-thio-β-D-galactopyranoside (S3)

Compound **S14** (2.10 g, 8.81 mmol) was dissolved in anhydrous acetonitrile (30 mL) and degassed with nitrogen. P- Toluenesulfonic acid (1.67 g, 8.81 mmol) and benzaldehyde dimethyl acetal (3.98 mL, 26.43 mmol) was added at 23 °C and stirred for 3.5 h. Upon completion the reaction was quenched with Et_3N until reaction is neutral. The crude mixture was purified via Teledyne ISCO CombiFlash® Rf 200i in hexane ethyl acetate step wise gradient with elution of compound in 80 % ethyl acetate. The product fractions were collected, coevaporated with toluene, concentrated, and placed on high vacuum to give 1.59 g, 4.88 mmole, 55 % as a white solid; R_f 0.2 (1:4 hexane: ethyl acetate);**^{1}H NMR** (400MHz ,CHLOROFORM-d) δ 7.53 - 7.40 (m, 2 H), 7.38 - 7.26 (m, 3 H), 5.43 (s, 1 H), 4.31 - 4.18 (m, 2 H), 4.08 (d, J = 3.1 Hz, 1 H), 3.90 (d, J = 11.3 Hz, 1 H), 3.74 (t, J = 9.4 Hz, 1 H), 3.58 (dd, J = 5.1, 7.4 Hz, 1 H), 3.33 (br. s., 2 H), 3.22 (s, 1 H), 2.72 (ddd, J = 6.2, 8.6, 12.5 Hz, 1 H), 2.67 - 2.57 (m, 1 H), 1.77 - 1.55 (m, 2 H), 0.97 (t, J = 7.4 Hz, 3 H);

^{13}C NMR (101MHz ,CHLOROFORM-d) δ 137.8, 129.2, 128.2, 126.5, 101.3, 85.3, 75.7, 73.7, 69.9, 69.5, 69.2, 31.2, 23.6, 13.6;

HRMS (ESI): [M + Na]$^+$ *m/z* calc. for $C_{16}H_{22}O_5SNa^+$ 349.1080 found 349.1080

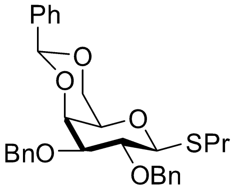

n-Propyl-6,4-benzylidene-2,3-di-O-benzyl-1-thio-β-D-galactopyranoside (16)

Compound **S3** (1.59 g, 4.88 mmol) was dissolved in anhydrous dimethylformamide (30 mL) at 0 °C and sodium hydride (0.58 g, 14.63 mmol) was slowly added. The reaction was stirred for 30 min and benzyl bromide (1.74 mL, 14.63 mmol) was added and the reaction was gradually raised to 23 °C and stirred for 12 h. Upon completion the reaction was quenched with methanol, diluted with dichloromethane (~60 mL) and washed with 2 M aqueous hydrochloric acid (~30 mL, 2x), saturated sodium bicarbonate (30 mL, 2x), and water (30 mL, 2x). The organic layer was dried over sodium sulfate, concentrated under reduced pressure and recrystallized in 200 proof ethanol to give 1.6 g, 3.16 mmol, 65% as a white solid; R_f 0.4 (2:1 Hexane/ Ethyl Acetate);**^{1}H NMR** (500MHz ,CHLOROFORM-d) δ 7.63 - 7.26 (m, 15 H), 5.51 (s, 1 H), 4.95 - 4.86 (m, 2 H), 4.79 (d, J = 1.0 Hz, 2 H), 4.83 - 4.74 (m, 2 H), 4.45 (d, J = 9.8 Hz, 1 H), 4.34 (dd, J = 1.2, 12.4 Hz, 1 H), 4.18 (d, J = 3.4 Hz, 1 H), 4.02 - 3.88 (m, 2 H), 3.62 (dd, J = 3.4, 9.3 Hz, 1 H), 3.38 (d, J = 1.0 Hz, 1 H), 2.91 - 2.67 (m, 2 H), 1.84 - 1.66 (m, 2 H), 1.04 (t, J = 7.6 Hz, 3 H)

^{13}C NMR (126MHz ,CHLOROFORM-d) δ 138.4, 138.3, 138.0, 129.1, 128.4, 128.3, 128.2, 127.8, 127.8, 127.7, 126.6, 101.5, 84.5, 81.1, 76.9, 75.7, 74.0, 71.8, 69.7, 69.4, 31.7, 23.4, 13.7;

HRMS (ESI): [M + Na]$^+$ *m/z* calc. for $C_{30}H_{34}O_5SNa^+$ 529.2019 found 529.2023

General Procedure for benzyl ether deprotection attempts using H-Cube Pro^{TM} Hydrogenation ThalesNano system:

The $Pd(OH)_2/C$ CatCart was inserted into the system and air was removed from the HPLC pump and inlet lines by placing the solvent and reactant lines in the reaction solvent. The inlet valve position on the monitor was set to solvent and the flow rate was set to 1 mL/min. A stainless steel needle with syringe was fasten to the inlet pressure sensor and while the pump is on and the pressure sensor

screw is unscrewed, air was sucked out of the solvent inlet line and pump. Once there is no air bubbles noticed, the inlet valve position was set to reactant and the same procedure was performed. The HPLC pump was stopped and the syringe was disconnected and inlet pressure sensor was screwed finger tight. The solvent line and reactant line were placed in their respective containers. The inlet valve on the monitor was set to solvent and the outlet valve on the monitor was set to waste, and the play button was pressed. The flow rate, pressure, and temperature were set to their desired specs and the protocol was started. Once the instrument has reached the desired specs and builds up the appropriate amount of hydrogen and is under the stable condition, the reactant tube was placed in a graduated cylinder containing the starting material and the product tube was placed in a flask. The instrument monitor inlet line was set to reactant and the outlet line was set to product. Once nearly full consumption of reactant has been flowed through the system, additional reactant solvent (CH_2Cl_2/ MeOH, 1:1) was flowed through the reactant line to flush any reactant that may be left. Once complete, the product output was analyzed by TLC initially and the instrument was stopped.

Recycling of the product solution was performed on entries 1-3 when TLC showed the incomplete consumption of starting material. Recycling was performed by placing the product outlet line into the graduated cylinder containing the reactant solution. Once multiple passes have been performed (3x), the instrument monitor inlet line was switched to solvent and the reactant inlet line was placed in solvent (CH_2Cl_2/ MeOH). The instrument monitor inlet line was switched back to reactant and the solvent was allowed to flow to flush any reactant/product that may be in the lines.

Table 1 SI. Deprotection attempts of n-Propyl-6,4-benzylidene-2,3-di-O-benzyl-1-thio-β-D-galactopyranoside under H-Cube Pro™ Hydrogenation ThalesNano system.

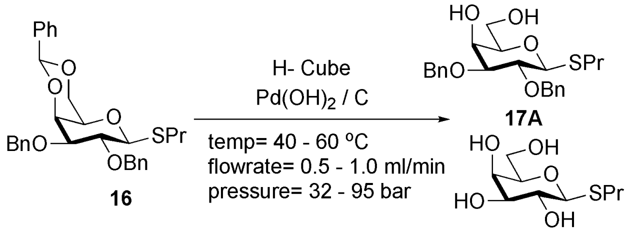

Entry	Flowrate (mL/min)	Pressure (bar)	Temp (°C)	Yield (%)
1	1	32.05	60	**A** 15
2	0.75	35.5	40	**A** 8.5
3	0.5	95	60	**A** 13.2
4	0.5	70	60	SM

Note: Entry 1 & 3 contained 10 equiv of acetic acid. Starting material (SM)

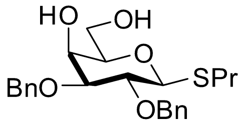

n-Propyl-2,3-di-O-benzyl-1-thio-β-D-galactopyranoside (17A)

General Procedure for benzyl ether deprotection attempts using H-Cube Pro™ Hydrogenation ThalesNano system.

^{1}H NMR (500MHz ,CHLOROFORM-d) δ 7.43 - 7.21 (m, 10 H), 4.88 - 4.59 (m, 5 H), 4.40 - 4.23 (m, 2 H), 4.12 - 3.94 (m, 3 H), 3.86 - 3.77 (m, 1 H), 3.68 (d, *J* = 9.3 Hz, 1 H), 3.56 - 3.37 (m, 3 H), 2.74 - 2.44 (m, 2 H), 1.59 (d, *J* = 7.3 Hz, 2 H), 1.00 - 0.84 (m, 3 H)

No further characterization

Entry 1: 6.2 mg, 14.81 µmol, 15%

Entry 2: 3.5 mg, 8.36 µmol, 8.5%

Entry 3: 3.0 mg, 7.17 µmol, 13.2%

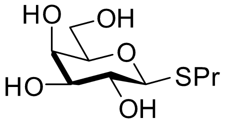

n-Propyl-1-thio-β-D-galactopyranoside (17B)

Thioglycoside **12** (16.0 mg, 0.03158 mmol) was dissolved in THF (2 mL), and liquid ammonia (16 mL) was condensed into the flask using a cold finger at -78 °C. Sodium (70 mg) was then added to the -78 °C reaction flask and stirred until a persistent blue color was obtained. Solid NH_4Cl (100 mg) was then added, and the solvents were allowed to evaporate under air. The crude reaction mixture was purified by chromatography on silica gel in 7:1 CH_2Cl_2/MeOH. The product fractions were collected, concentrated, and placed on high vacuum to give 7.4 mg, 31.05 µmol, 98% as an oil; R_f 0.26 (7:1 CH_2Cl_2/MeOH); Proton matches compound **S14** in the synthesis towards **16**.

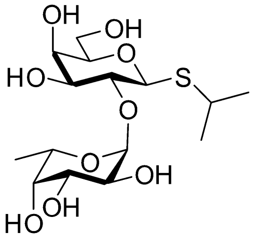

2-Propyl thio-alpha-L-fuco-pyranosyl-(1-2)-beta-D-galactopyranoside (2a)

Compound **13** (14.8 mg, 16.0 µmol) was dissolved in anhydrous THF (2.1 mL), and liquid ammonia (8.2 mL) was condensed into the flask using a cold finger at -78 °C. Sodium (15 mg) was then added and stirred until a persistent blue color was obtained. The reaction was stirred for 2.5 h at -78 °C, while added additional sodium every 40 min to maintain the persistent blue color. Methanol (5 mL) was then added to quench the reaction, and the solvent was allowed to evaporate under air. The crude material was purified through solid phase extraction, using a C18 Sep Pak, and the product eluted in 50% acetonitrile in water, 0.1% formic acid, and the solvents were removed by lyophilization. Partially debenzylated impurities were then removed semipreparative HPLC on a Phenomenex C-18 column (100 Å, 5 µm, 250 x 10 mm) using the method in the table below to give the purified compound **2a** (3.3 mg, 29%, combined material from two separate reactions) as a white solid:

A = water, B = acetonitrile

Time (min)	A%	B%
0.00	90.0	10.0
12.00	58.0	42.0
13.00	0.0	100.0
15.00	0.0	100.0

^{1}H NMR (500 MHz, MeOD) δ 5.55 (d, *J* = 5.5 Hz, 1H), 4.93 (d, *J* = 3.6 Hz, 1H), 4.58 (s, 1H), 4.45 – 4.34 (m, 1H), 4.22 (t, *J* = 6.2 Hz, 1H), 4.02 (dd, *J* = 10.1, 5.6 Hz, 1H), 3.98 – 3.91 (m, 1H), 3.80 (dd, *J* = 10.1, 3.3 Hz, 1H), 3.75 – 3.56 (m, 5H), 3.51 – 3.41 (m, 1H), 3.06 (p, *J* = 6.8 Hz, 1H), 1.30 (dd, *J* = 12.7, 6.8 Hz, 7H), 1.22 (d, *J* = 6.6 Hz, 3H).

HRMS (ESI): $[M + Na]^+$ *m/z* calc. for $C_{15}H_{28}O_9SNa^+$ 407.1346 found 407.1348

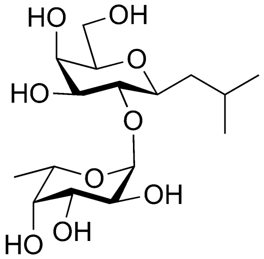

1-isobutyl-α-L-fucopyranose-(1-2)-β-D-galactopyranose (2b)

Compound **15α** (24.6 mg, 27.1 μmol) was dissolved in anhydrous THF (3.5 mL), and liquid ammonia (13.9 mL) was condensed into the flask using a cold finger at -78 °C. Sodium (50 mg) was then added and stirred until a persistent blue color was obtained. The reaction was stirred for 4 h at -78 °C, while added additional sodium every 40 min to maintain the persistent blue color. Methanol (10 mL) was then added to quench the reaction, and the solvent was allowed to evaporate under air. The crude material was purified through solid phase extraction, using a C18 Sep Pak, and the product eluted in 50% acetonitrile in water, 0.1% formic acid, and the solvents were removed by lyophilization to give the purified compound **2b** (2.0 mg, 20%) as a white solid.

^{1}H NMR (600 MHz, CD_3OD) δ 8.55 (s, 4H), 5.00 (d, *J* = 4.0 Hz, 1H), 4.58 (s, 1H), 4.06 (q, *J* = 6.6 Hz, 1H), 3.91 (d, *J* = 3.3 Hz, 1H), 3.80 (dd, *J* = 10.1, 3.9 Hz, 1H), 3.75 – 3.56 (m, 5H), 3.44 – 3.38 (m, 2H), 3.23 (t, *J* = 9.6 Hz, 1H), 1.93 – 1.87 (m, 1H), 1.73 – 1.66 (m, 1H), 1.47 (ddd, *J* = 14.1, 10.1, 4.0 Hz, 1H), 1.34 – 1.31 (m, 2H), 1.21 (d, *J* = 6.8 Hz, 3H), 0.97 – 0.89 (m, 7H).

HRMS (ESI) m/z $[M+Na]^+$ Calcd for $C_{16}H_{30}O_9Na$ 389.1782; Found 389.1783

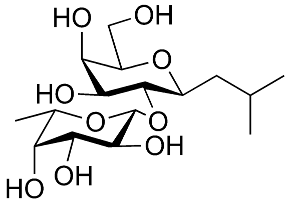

1-isobutyl-β-L-fucopyranose-(1-2)-β-D-galactopyranose (2c)

Compound **15β** (16.4 mg, 18.1 μmol) was dissolved in anhydrous THF (2.3 mL), and liquid ammonia (9.3 mL) was condensed into the flask using a cold finger at -78 °C. Sodium (33 mg) was then added and stirred until a persistent blue color was obtained. The reaction was stirred for 4 h at -78 °C, while added additional sodium every 40 min to maintain the persistent blue color. Methanol (mL) was then added to quench the reaction, and the solvent was allowed to evaporate under air. The crude material was purified through solid phase extraction, using a C18 Sep Pak, and the product eluted in 50% acetonitrile in water, 0.1% formic acid, and the solvents were removed by lyophilization to give the purified compound **2c** (1.5 mg, 23%) as a white solid.

^{1}H NMR (600 MHz, CD_3OD) δ 4.24 (d, *J* = 6.9 Hz, 1H), 3.95 (dd, *J* = 3.4, 1.0 Hz, 1H), 3.78 – 3.71 (m, 1H), 3.71 – 3.65 (m, 2H), 3.62 (dd, *J* = 3.0, 1.1 Hz, 1H), 3.54 – 3.46 (m, 3H), 3.45 – 3.39 (m, 2H), 3.35 (d, *J* = 1.8 Hz, 0H), 1.96 – 1.85 (m, 2H), 1.29 (d, *J* = 6.4 Hz, 3H), 0.97 – 0.88 (m, 7H).

HRMS (ESI) m/z $[M+Na]^+$ Calcd for $C_{16}H_{30}O_9Na$ 389.1782; Found 389.1784

^{1}H NMR, ^{13}C NMR, dqCOSY, HMBC, HSQC, HRMS of New Compounds

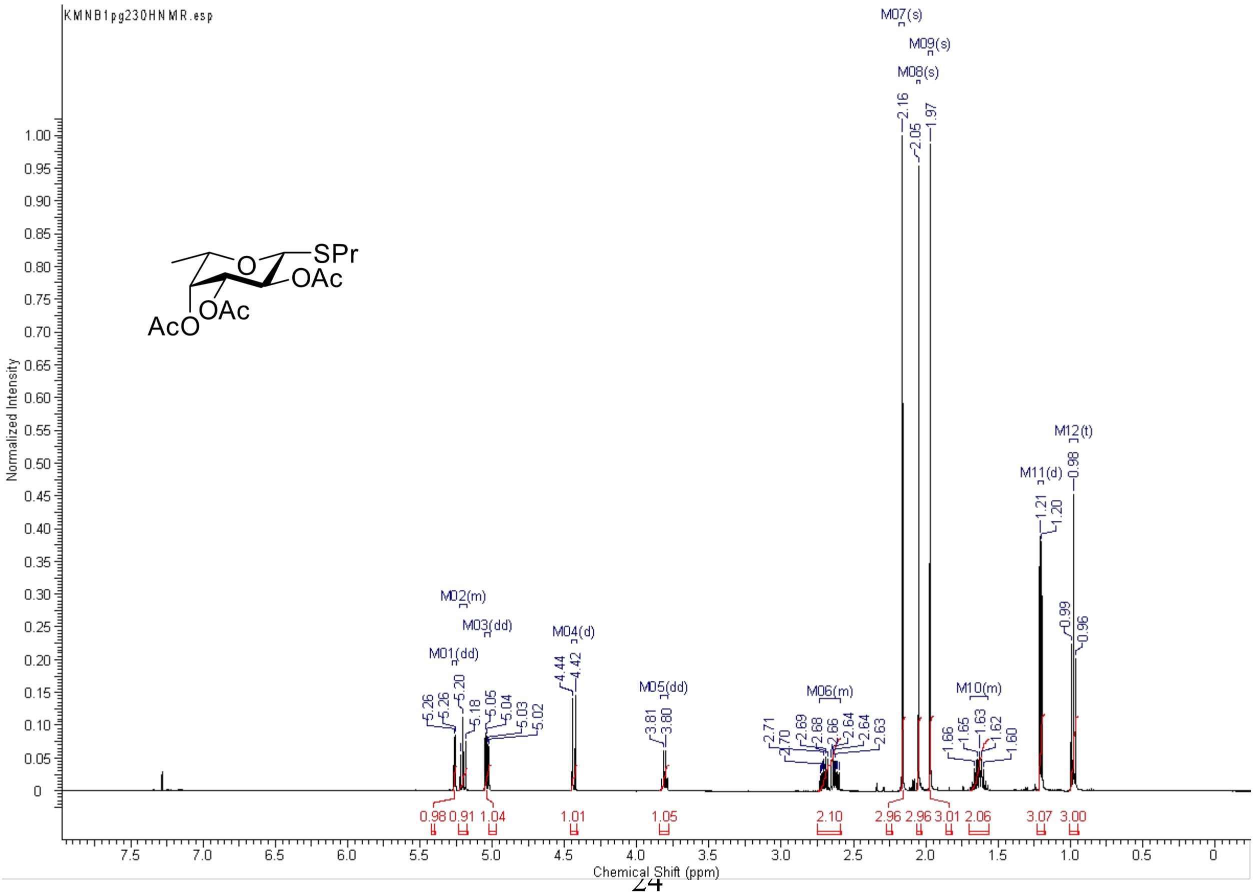

^{1}H NMR (500MHz ,CHLOROFORM-d) spectrum of compound **2**

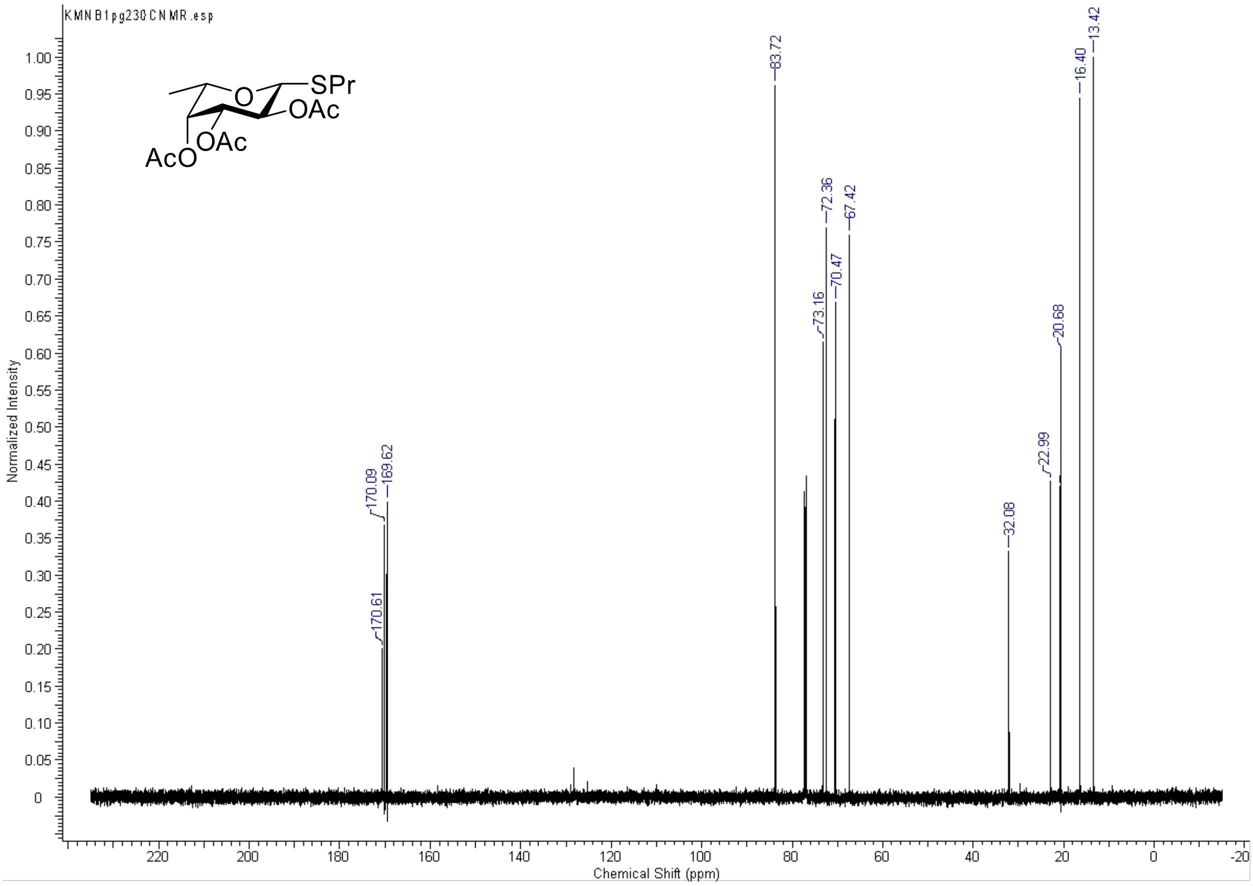

^{13}C NMR (126 MHz, $CDCl_3$) spectrum of compound **2**

KMNB1pg230dqCosy.fid.esp

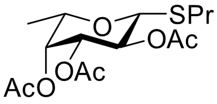

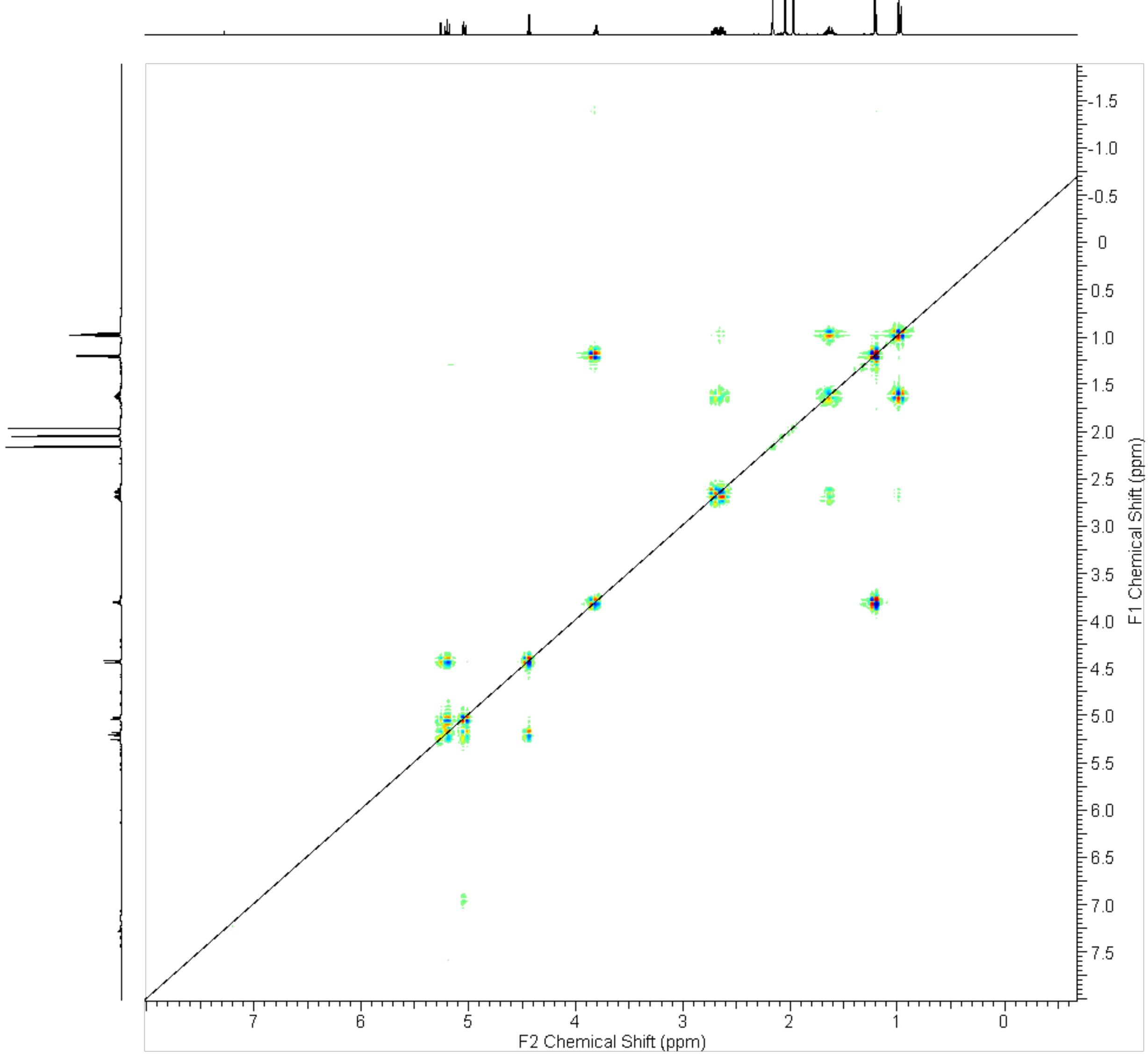

dqCOSY (500MHz ,CHLOROFORM-d) spectrum of compound **2**

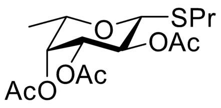

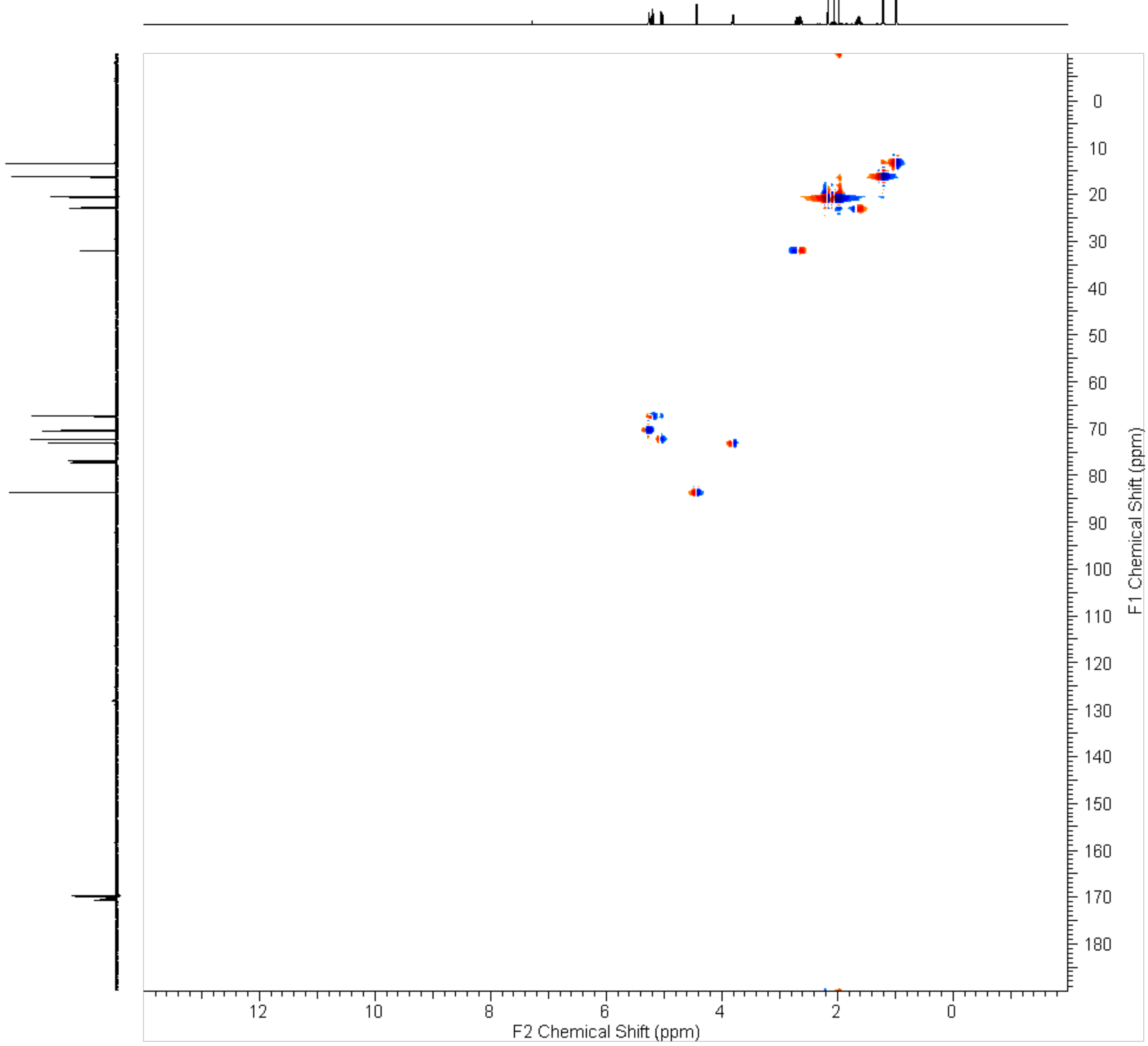

HSQC (500MHz ,CHLOROFORM-d) spectrum of compound **2**

SUPPORTING INFORMATION

KMNB1pg230HMBC.fid.esp

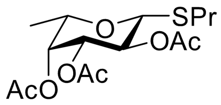

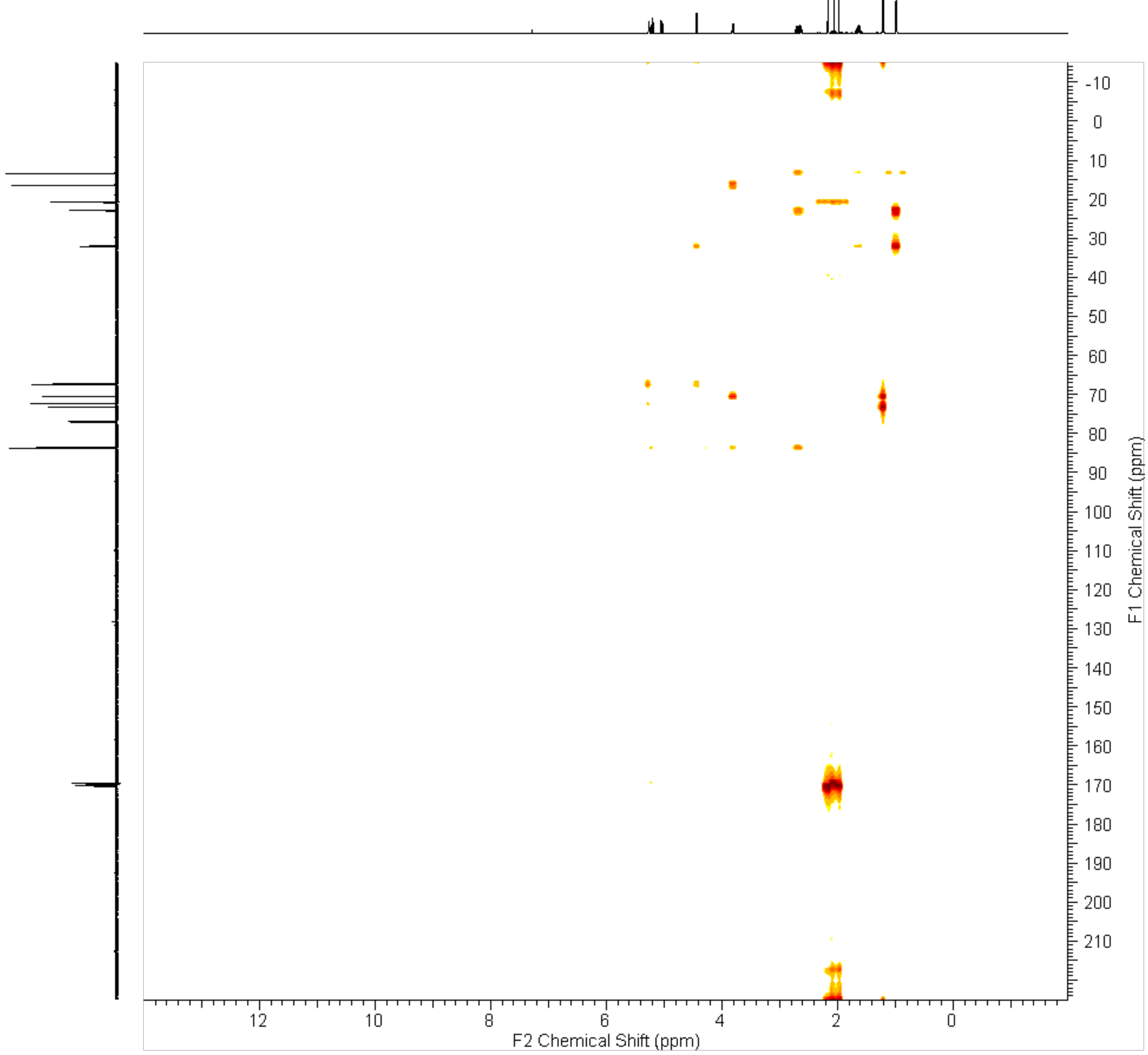

HMBC (500MHz ,CHLOROFORM-d) spectrum of compound **2**

Elemental Composition Report **Page 1**

Tolerance = 50.0 PPM / DBE: min = -1.5, max = 100.0
Element prediction: Off
Number of isotope peaks used for i-FIT = 3

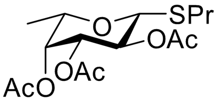

Monoisotopic Mass, Even Electron Ions
31 formula(e) evaluated with 1 results within limits (up to 50 closest results for each mass)
Elements Used:
C: 0-15 H: 0-24 O: 0-7 Na: 0-1 S: 0-1
kmnb1-pg230
kmnb1-pg230c 34 (0.634) AM2 (Ar,9000.0,430.91,0.70,LS 4); Cm (34:36) 1: TOF MS ES+ 8.06e+003

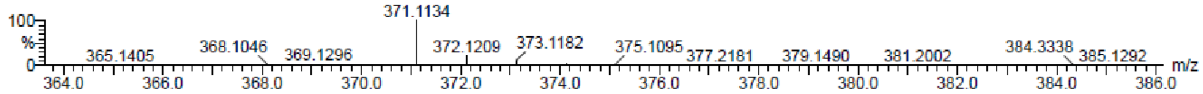

Minimum:	50.00				-1.5			
Maximum:	100.00		5.0	50.0	100.0			
Mass	RA	Calc. Mass	mDa	PPM	DBE	i-FIT	i-FIT (Norm)	Formula
371.1134	100.00	371.1140	-0.6	-1.6	3.5	147.3	0.0	C15 H24 O7 Na S

HRMS for Compound **2**

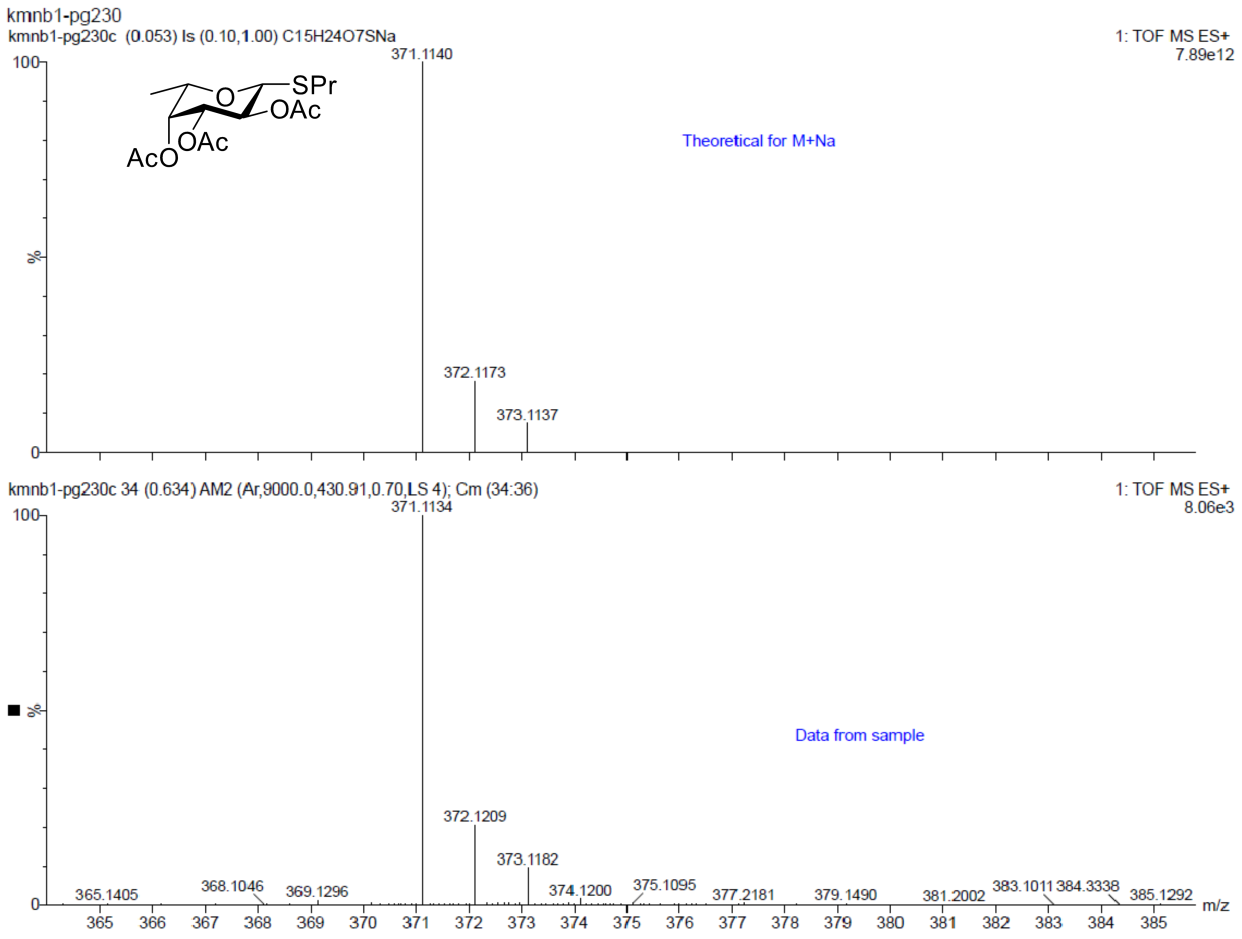

HRMS for compound **2**

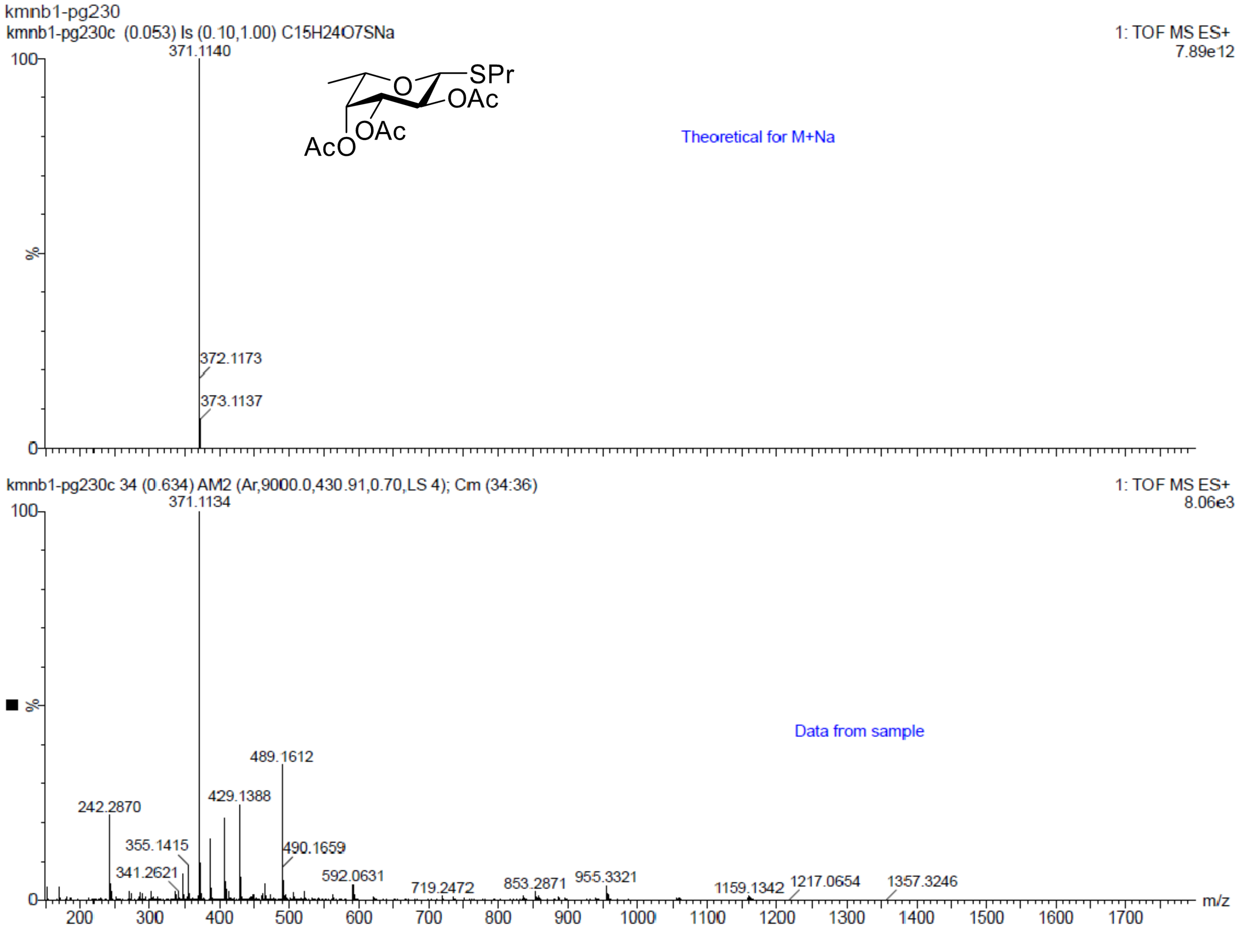

HRMS compound **2**

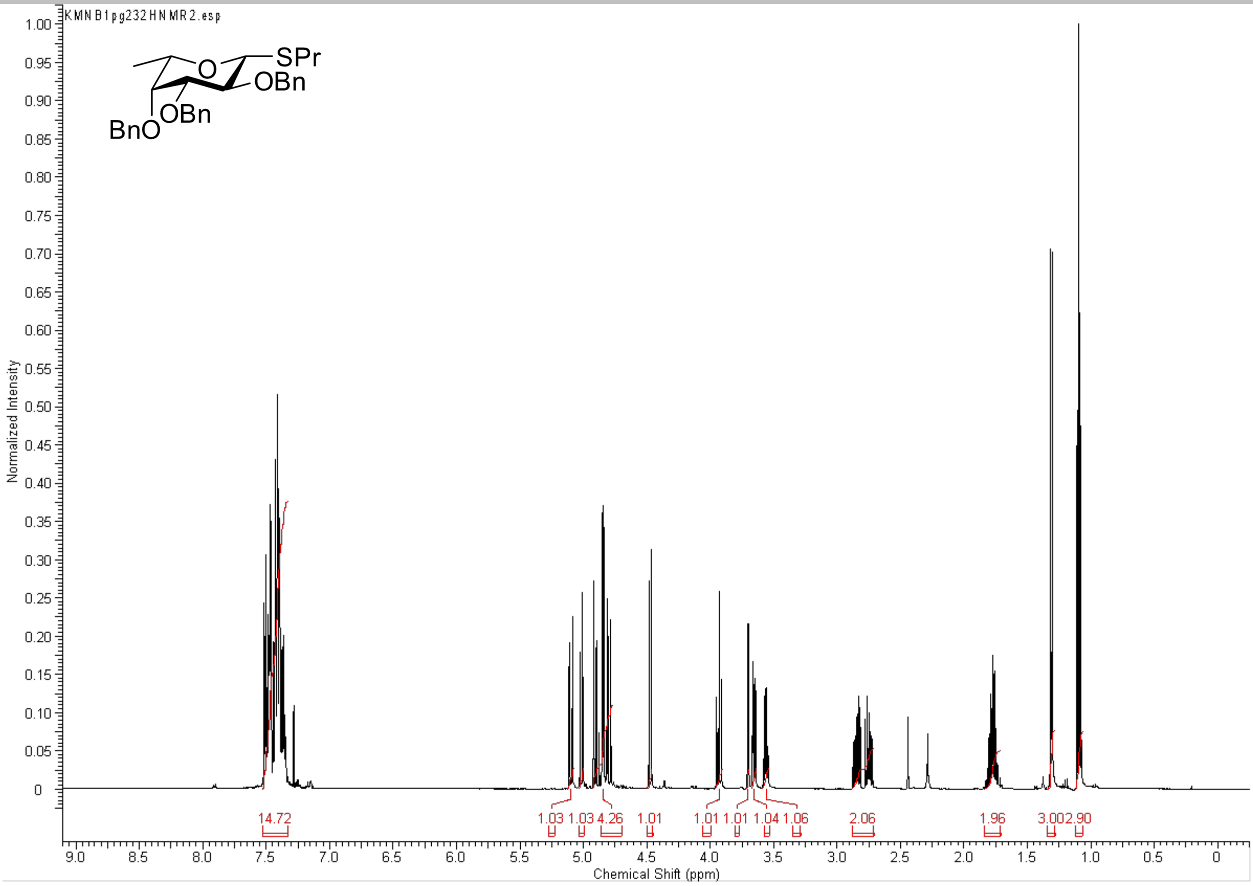

^{1}H NMR (500MHz ,CHLOROFORM-d) spectrum of compound **3**

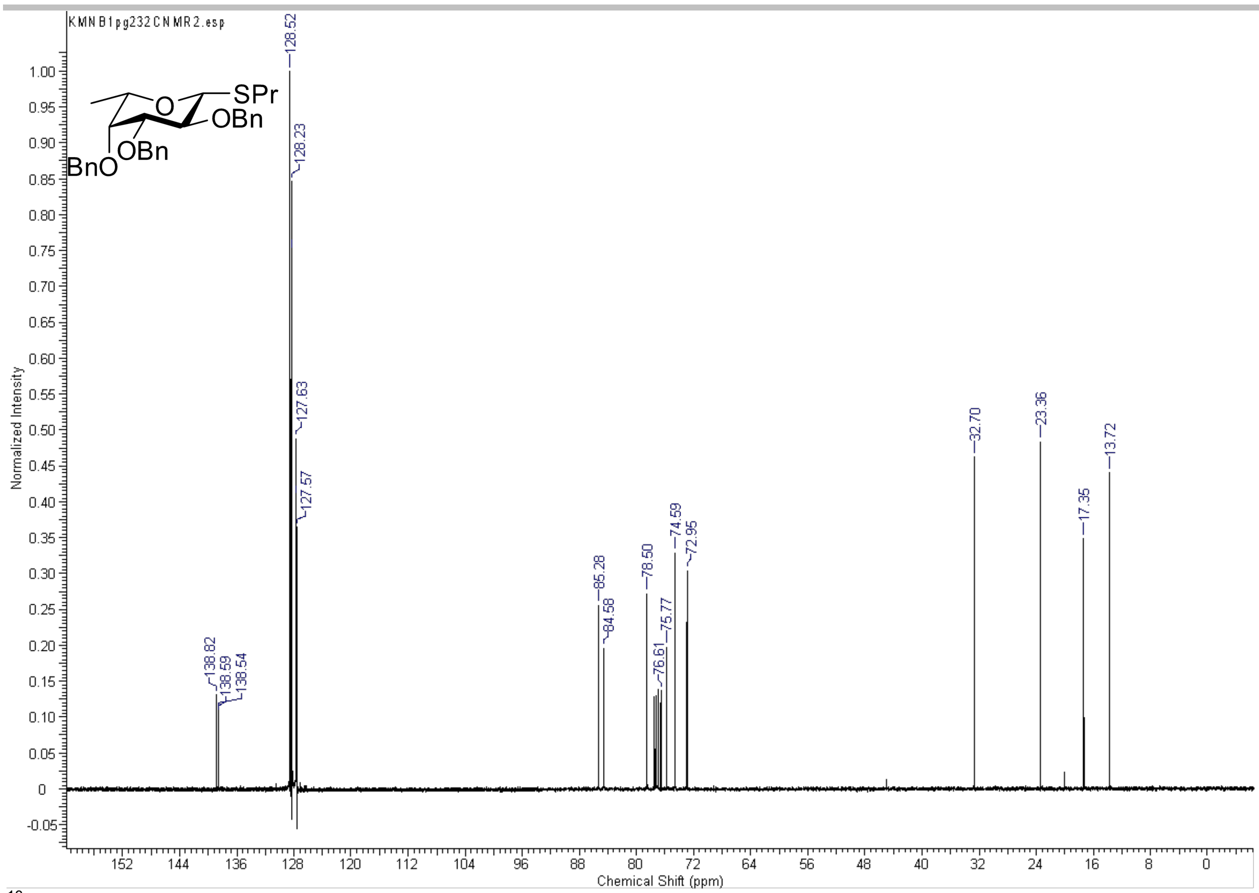

^{13}C NMR (126 MHz, $CDCl_3$) spectrum of compound **3**

KMNB1pg232dqCosy.fid.esp

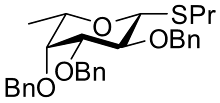

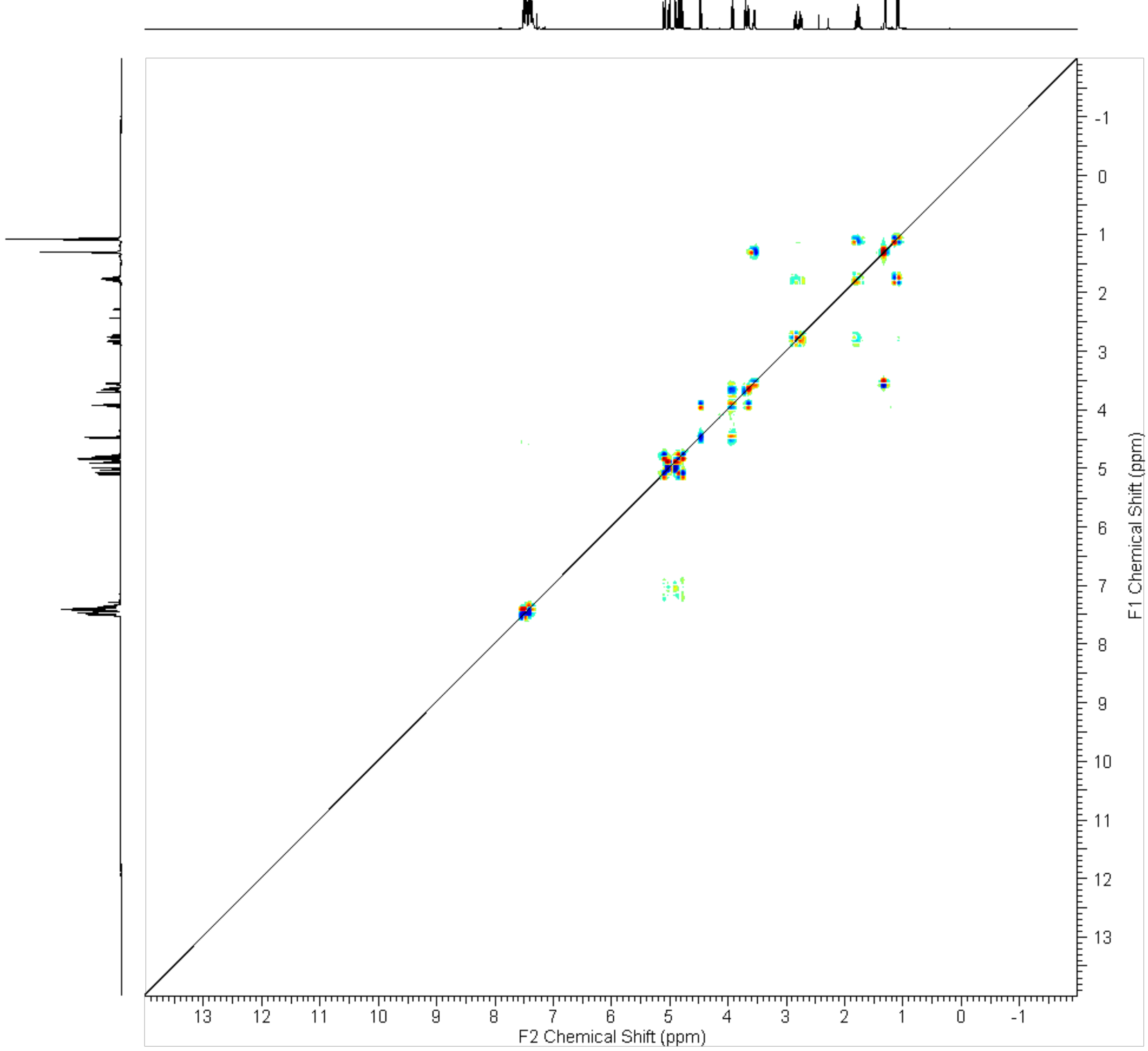

dqCOSY (500MHz ,CHLOROFORM-d) spectrum of compound **3**

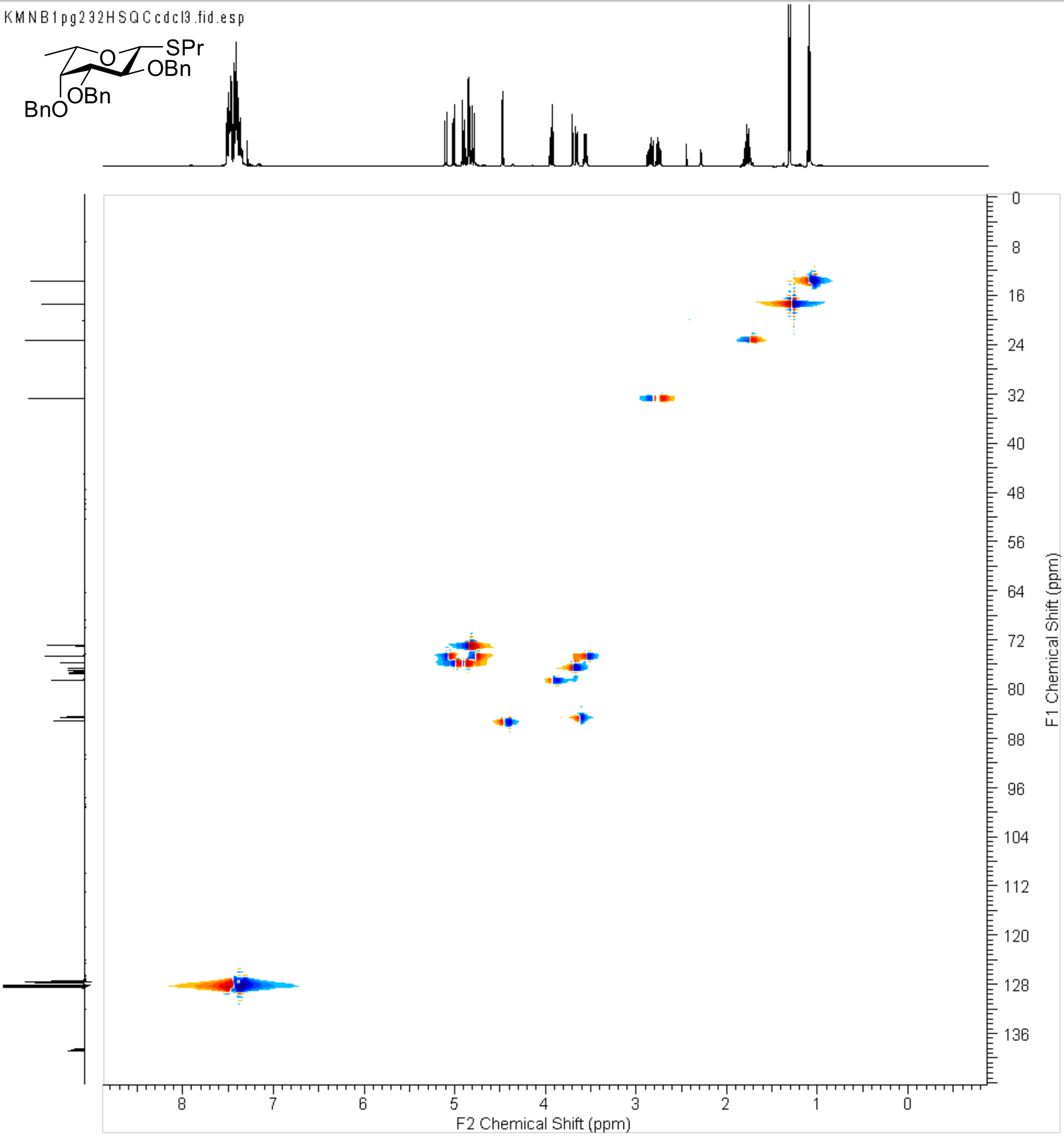

HSQC (500MHz ,CHLOROFORM-d) spectrum of compound **3**

KMNB1pg232HMBC.fid.esp

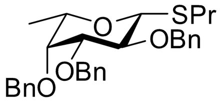

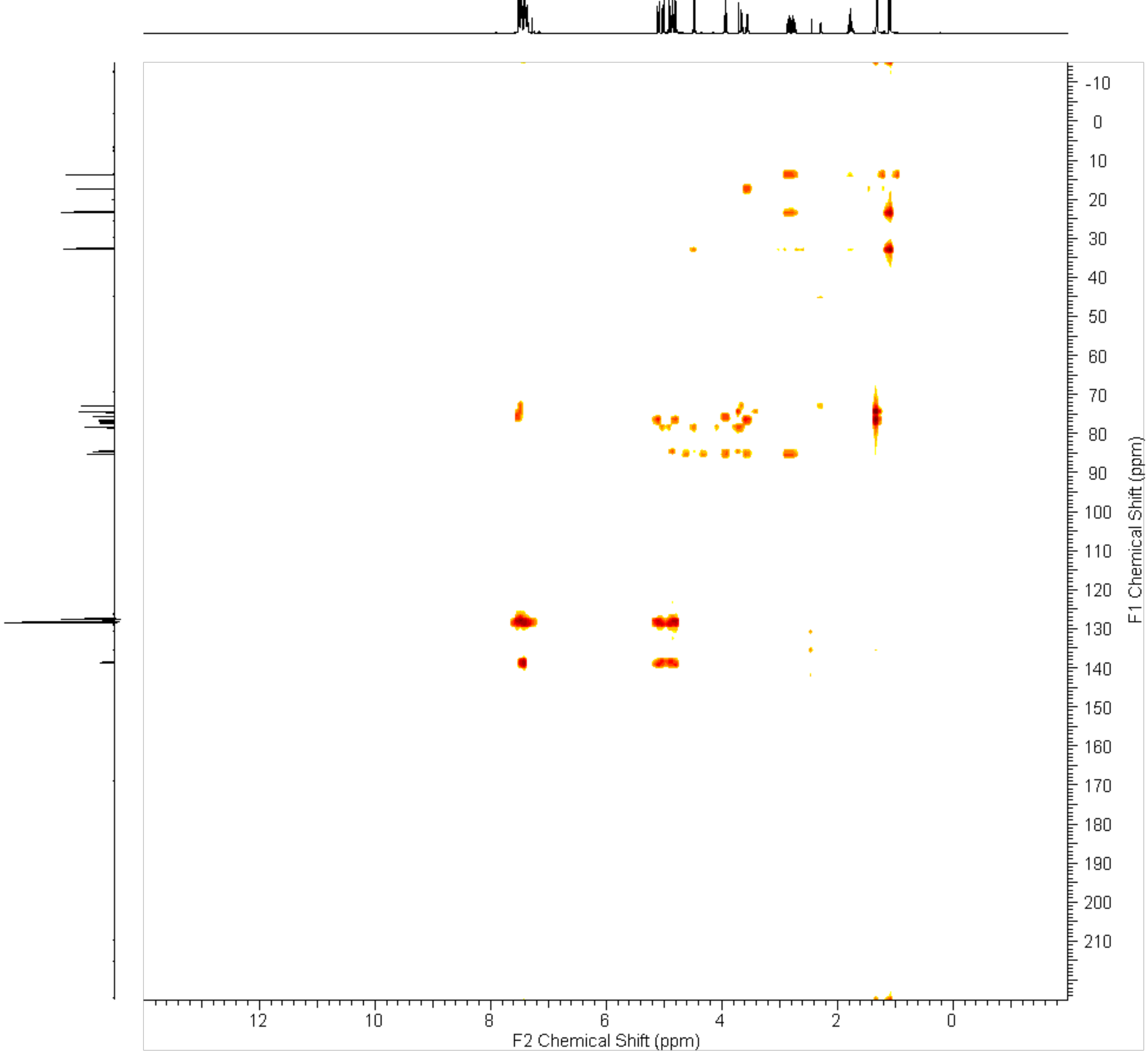

HMBC (500MHz ,CHLOROFORM-d) spectrum of compound **3**

Elemental Composition Report **Page 1**

Tolerance = 50.0 PPM / DBE: min = -1.5, max = 100.0
Element prediction: Off
Number of isotope peaks used for i-FIT = 3

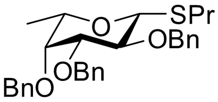

Monoisotopic Mass, Even Electron Ions
16 formula(e) evaluated with 1 results within limits (up to 50 closest results for each mass)
Elements Used:
C: 0-30 H: 0-36 O: 0-4 Na: 0-1 S: 0-1
kmnb1-pg232
kmnb1-pg232c 37 (0.705) AM2 (Ar,9000.0,566.89,0.70,LS 4) 1: TOF MS ES+ 1.17e+003

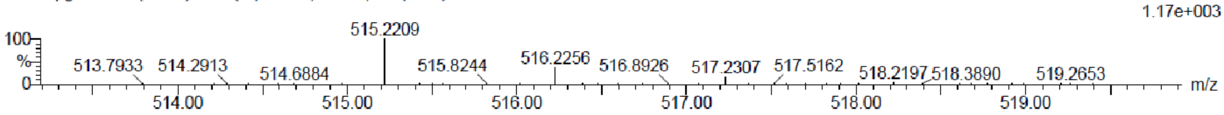

Minimum:	50.00				-1.5			
Maximum:	100.00		5.0	50.0	100.0			
Mass	RA	Calc. Mass	mDa	PPM	DBE	i-FIT	i-FIT (Norm)	Formula
515.2209	100.00	515.2232	-2.3	-4.5	12.5	67.1	0.0	C30 H36 O4 Na S

HRMS for compound **3**

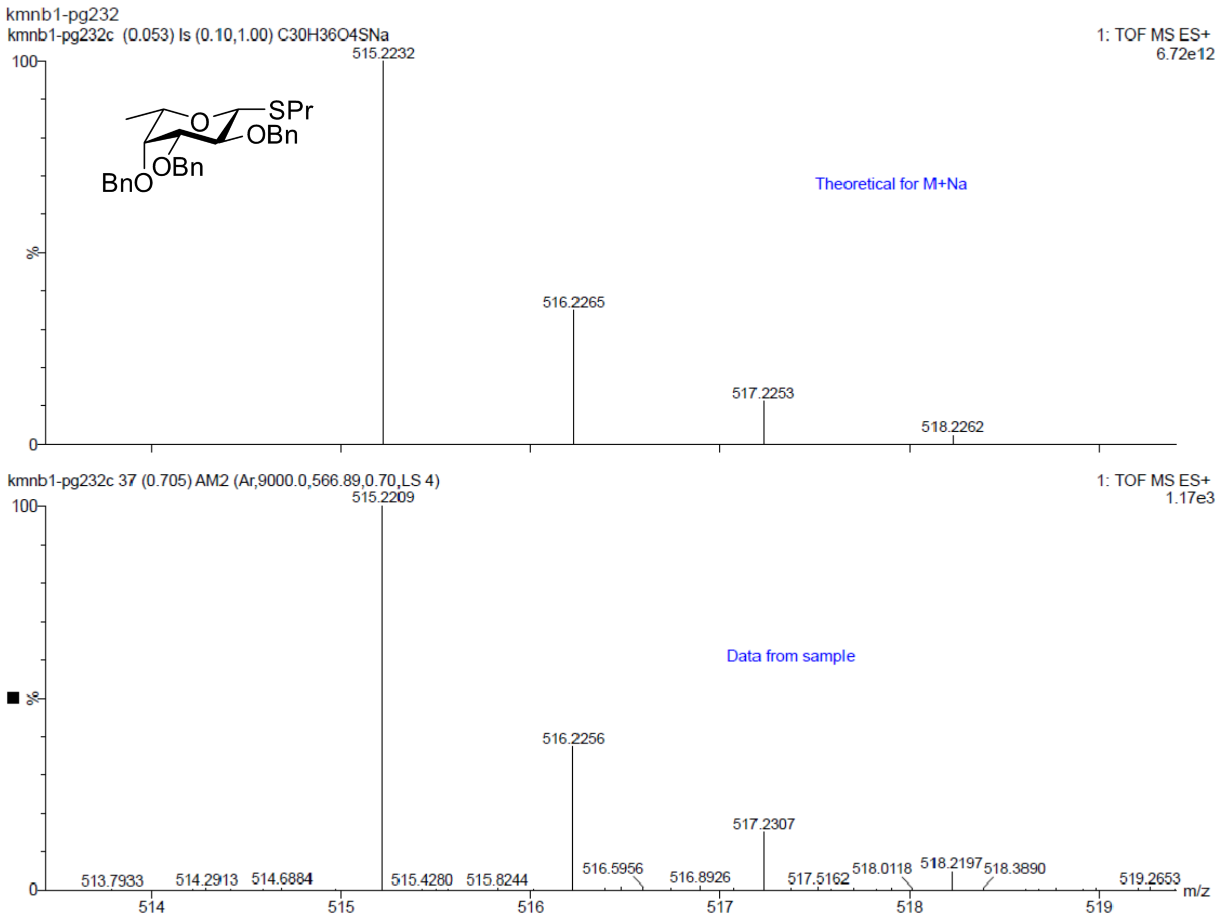

HRMS for compound **3**

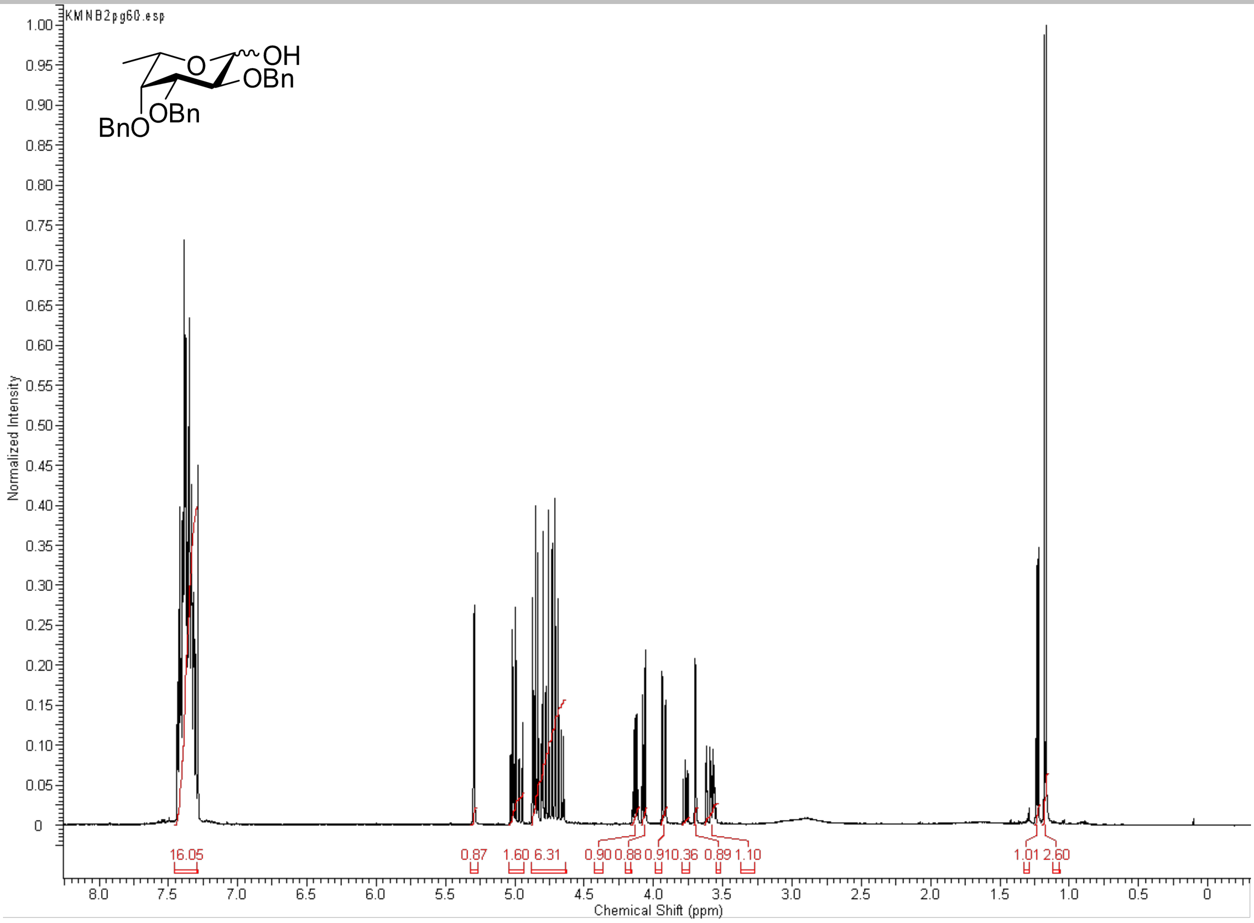

^{1}H NMR (500MHz ,CHLOROFORM-d) known compound

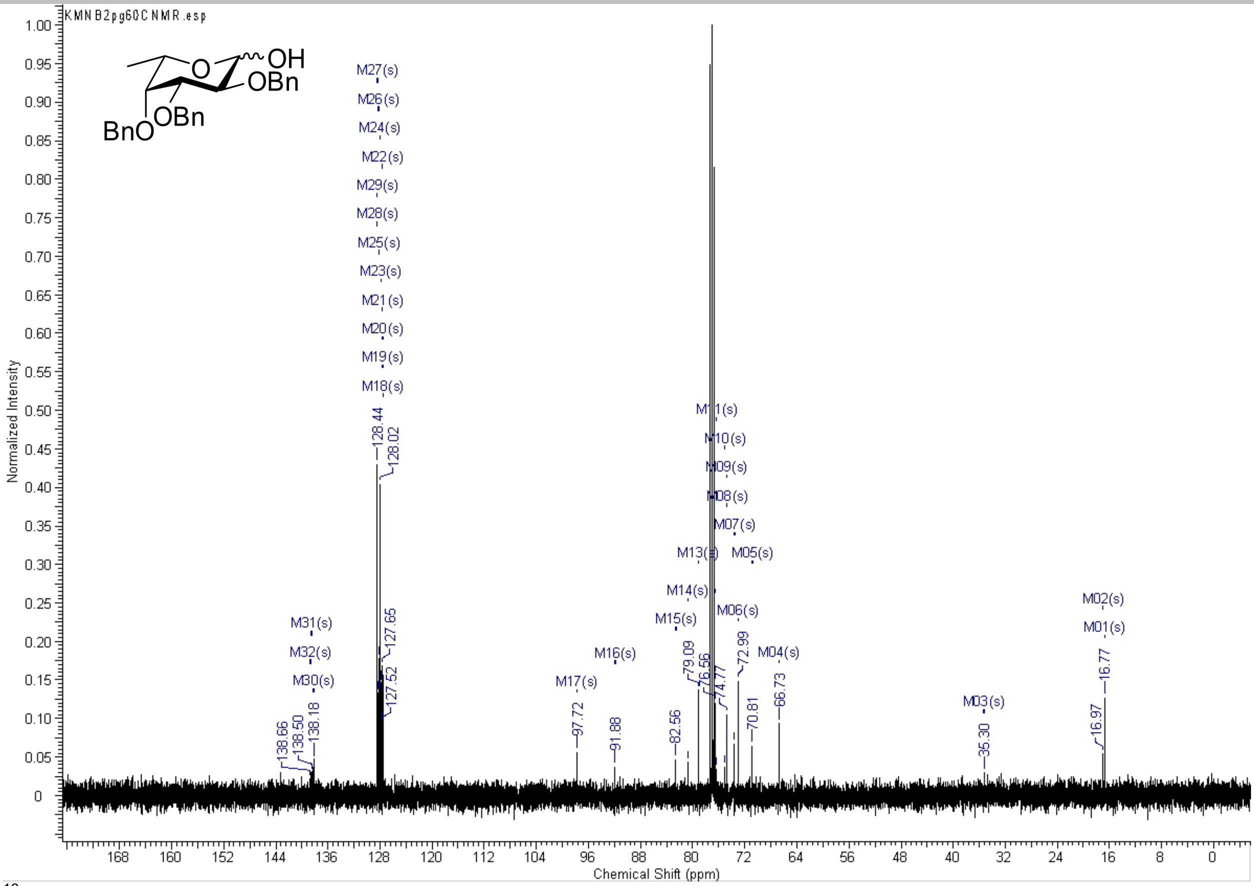

^{13}C NMR (126MHz ,CHLOROFORM-d) known compound

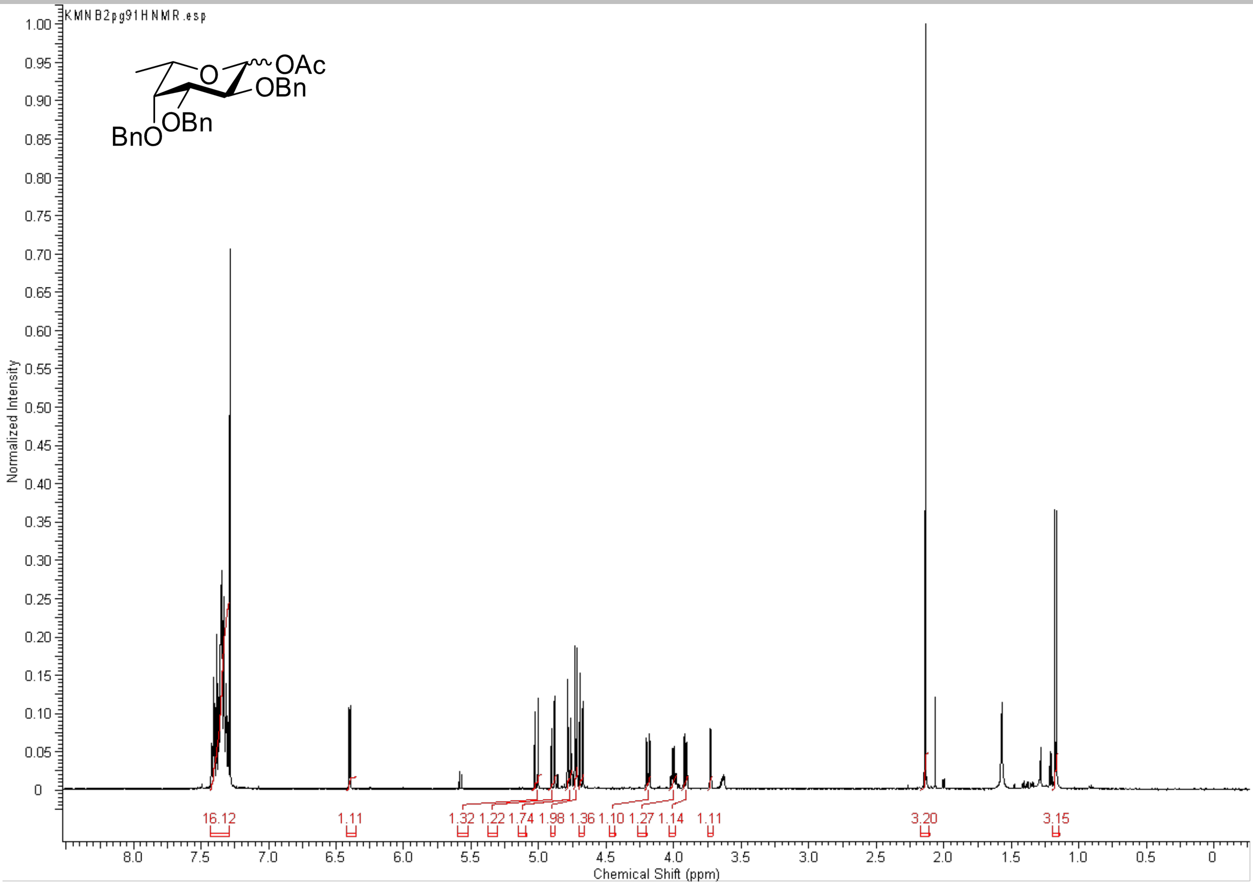

^{1}H NMR (500MHz ,CHLOROFORM-d) for compound **4**

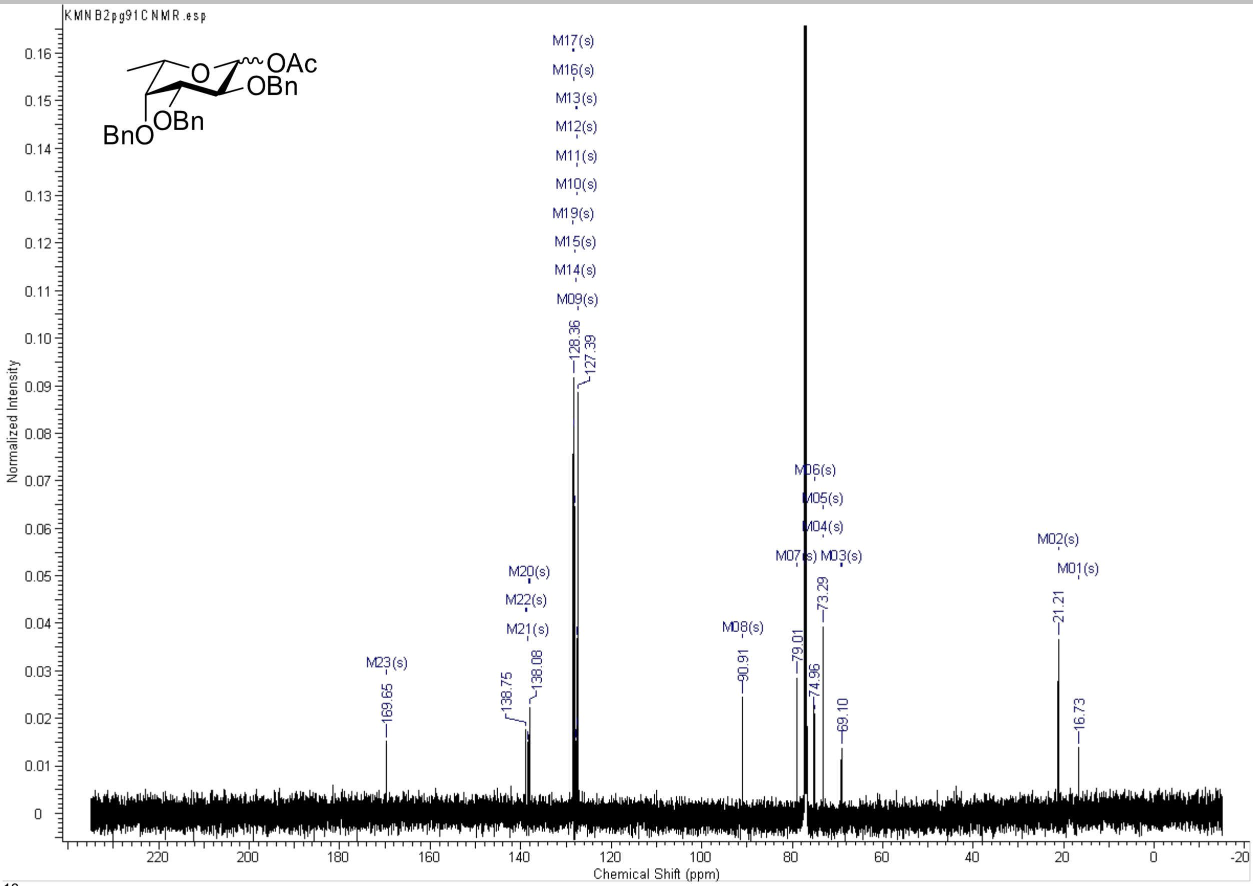

^{13}C NMR (126MHz ,CHLOROFORM-d) for compound **4**

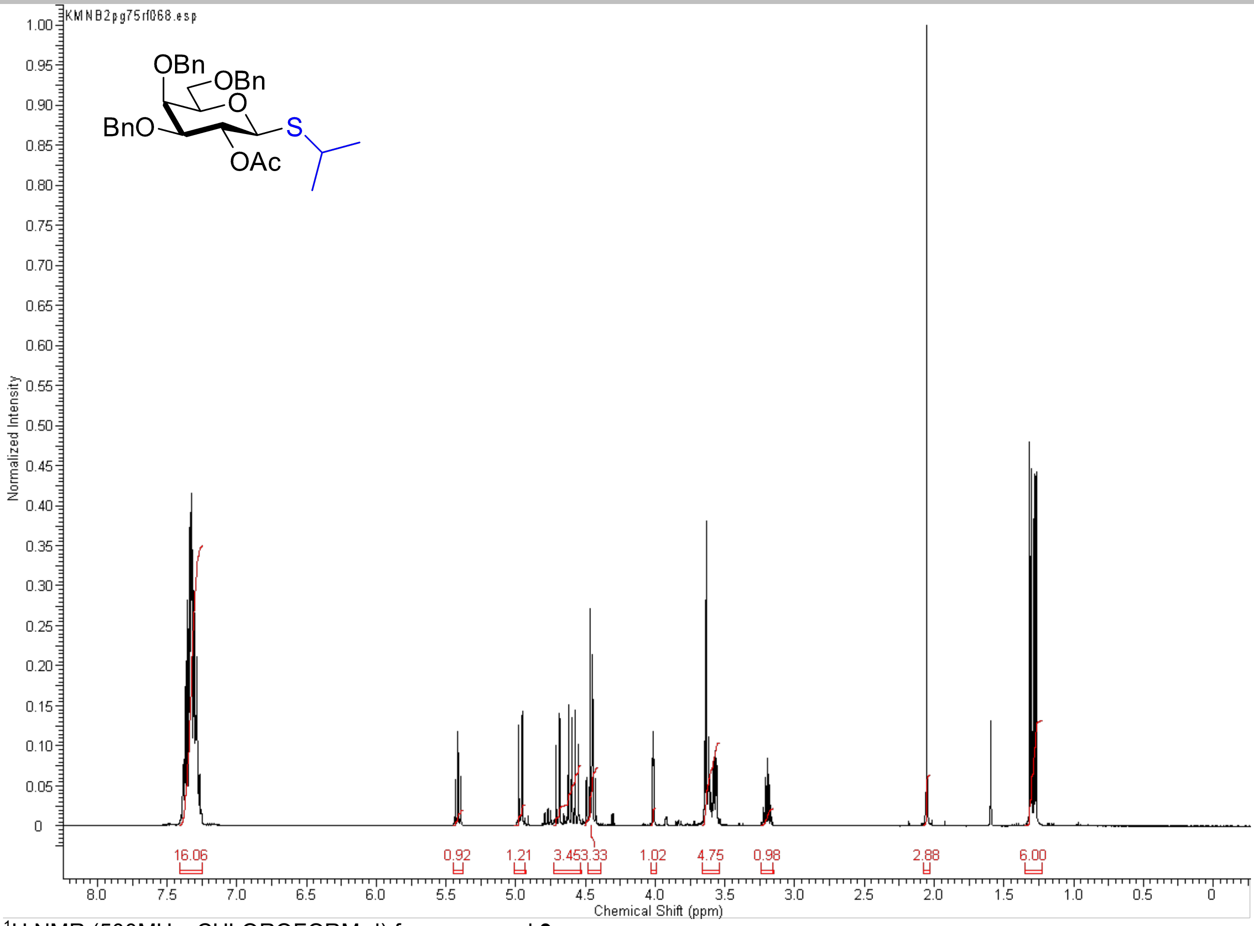

^{1}H NMR (500MHz ,CHLOROFORM-d) for compound **9**

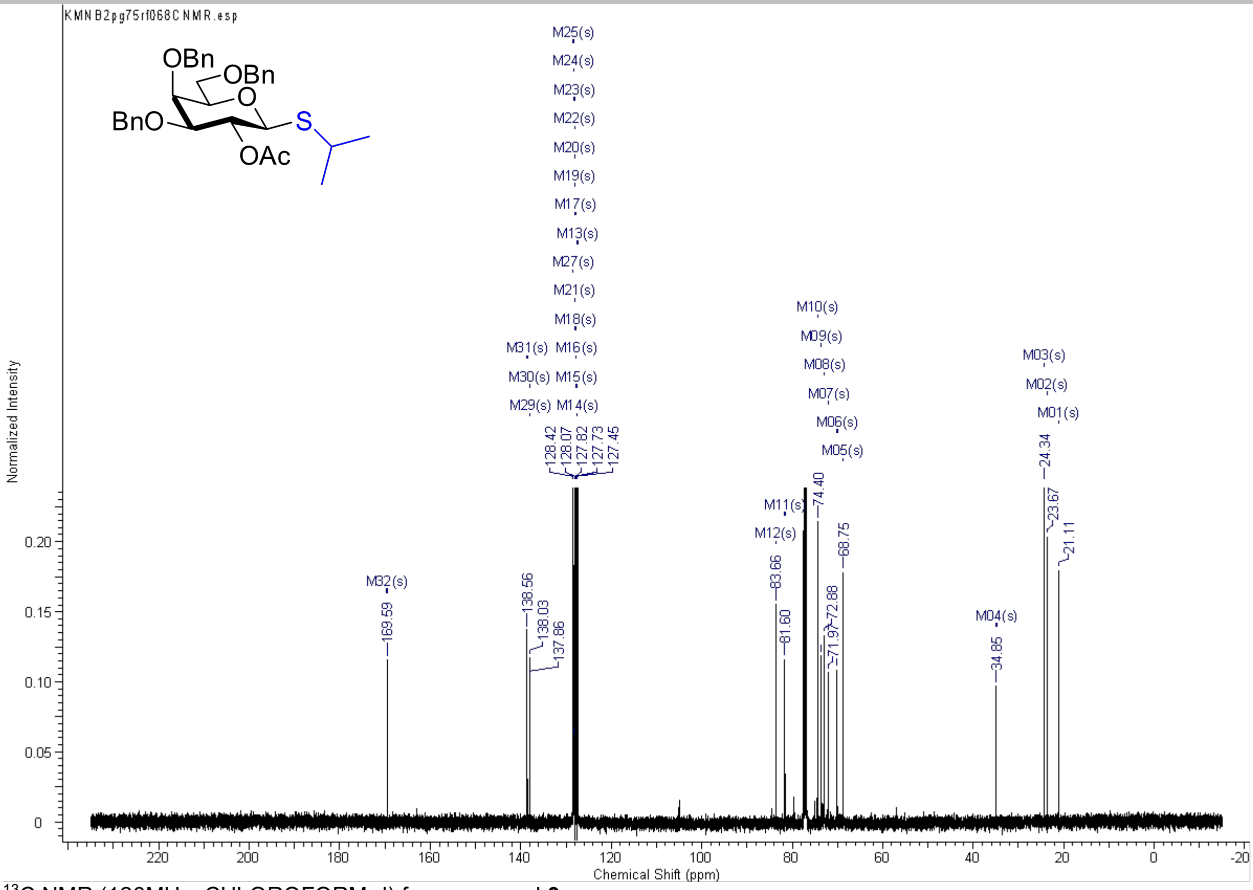

^{13}C NMR (126MHz ,CHLOROFORM-d) for compound **9**

KMNB2pg75rf068dqCOSY.fid.esp

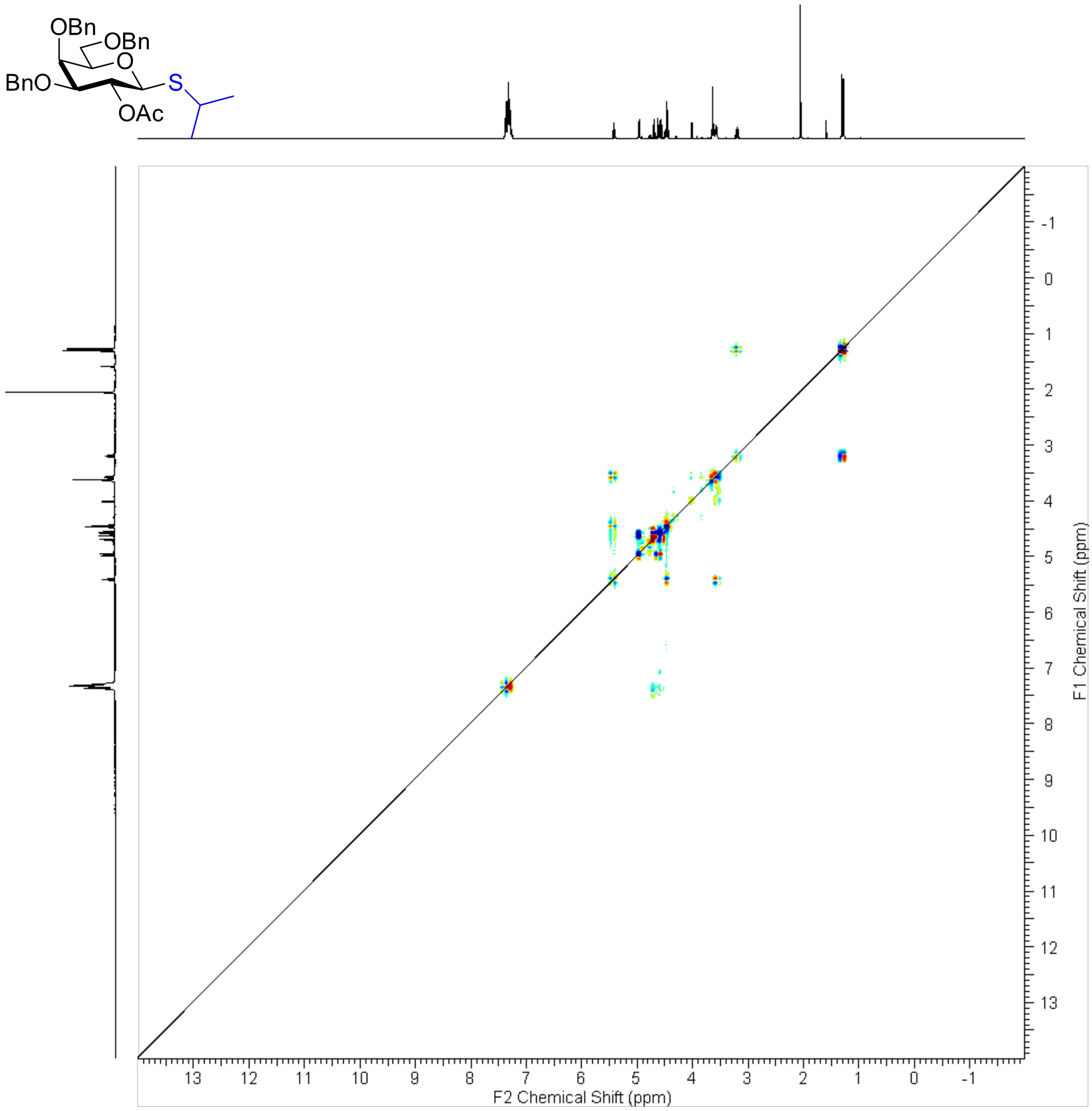

dqCOSY (500MHz ,CHLOROFORM-d) for compound **9**

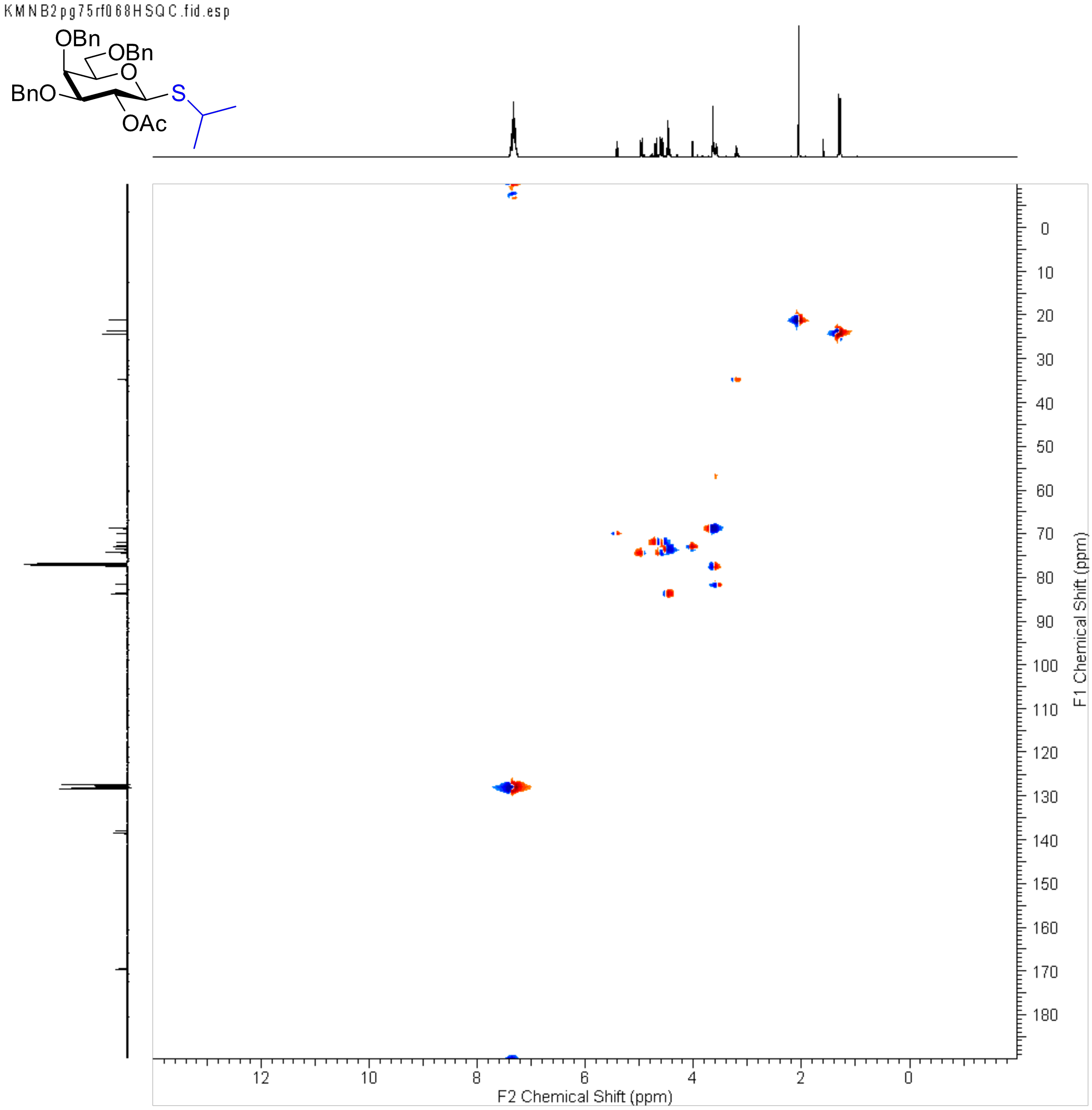

HSQC (500MHz ,CHLOROFORM-d) for compound **9**

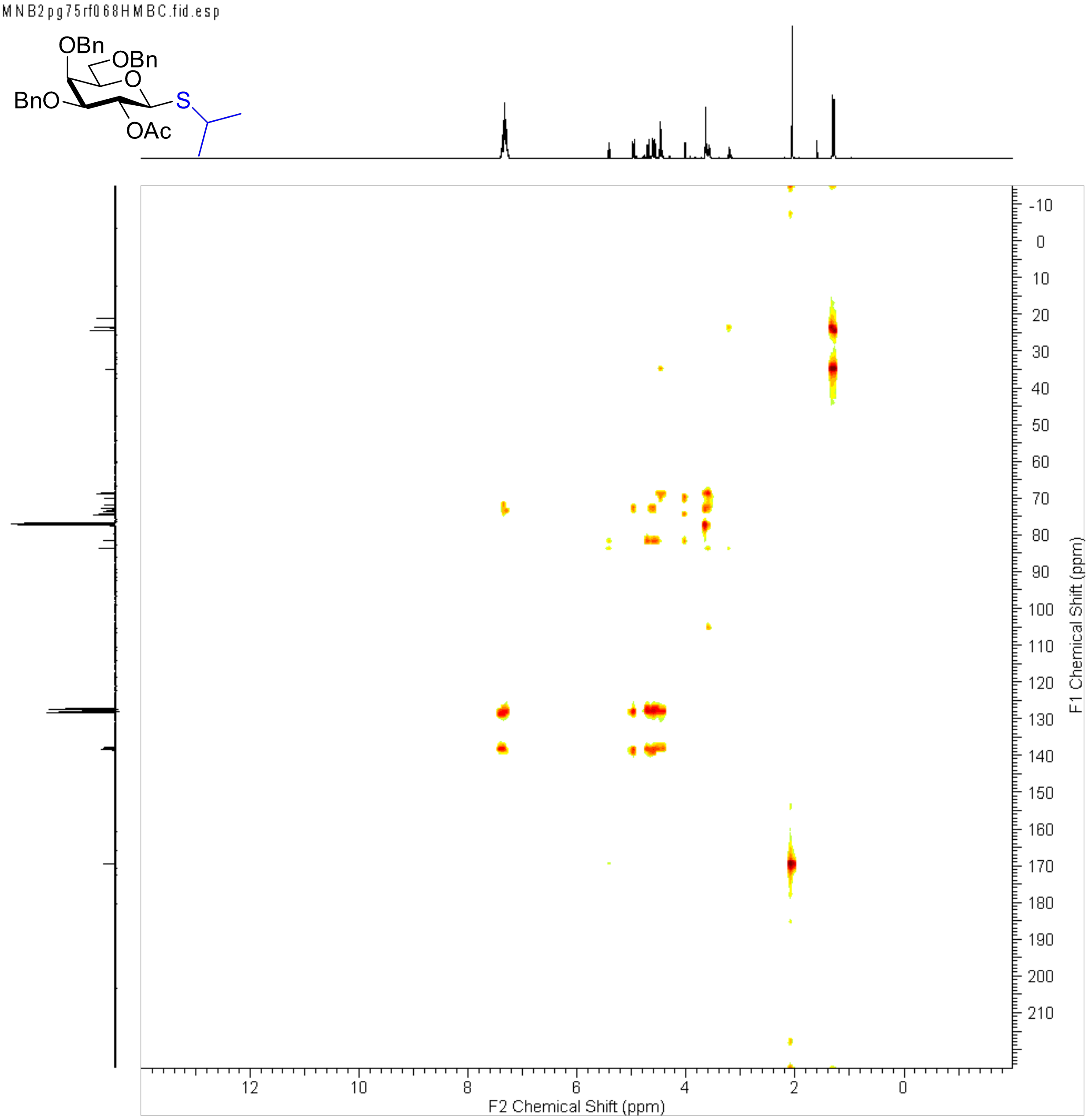

HMBC (500MHz ,CHLOROFORM-d) for compound **9**

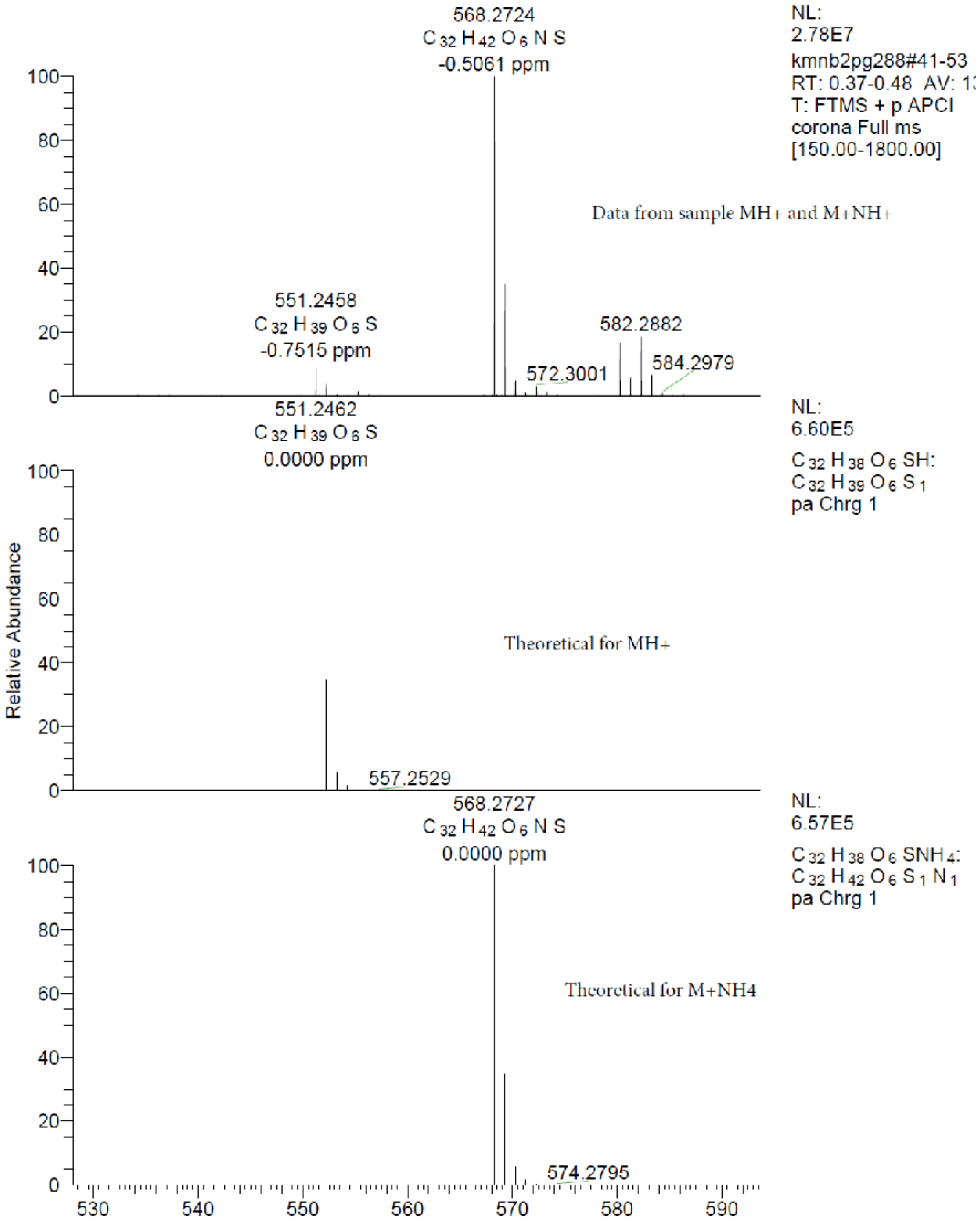

HRMS for compound **9**

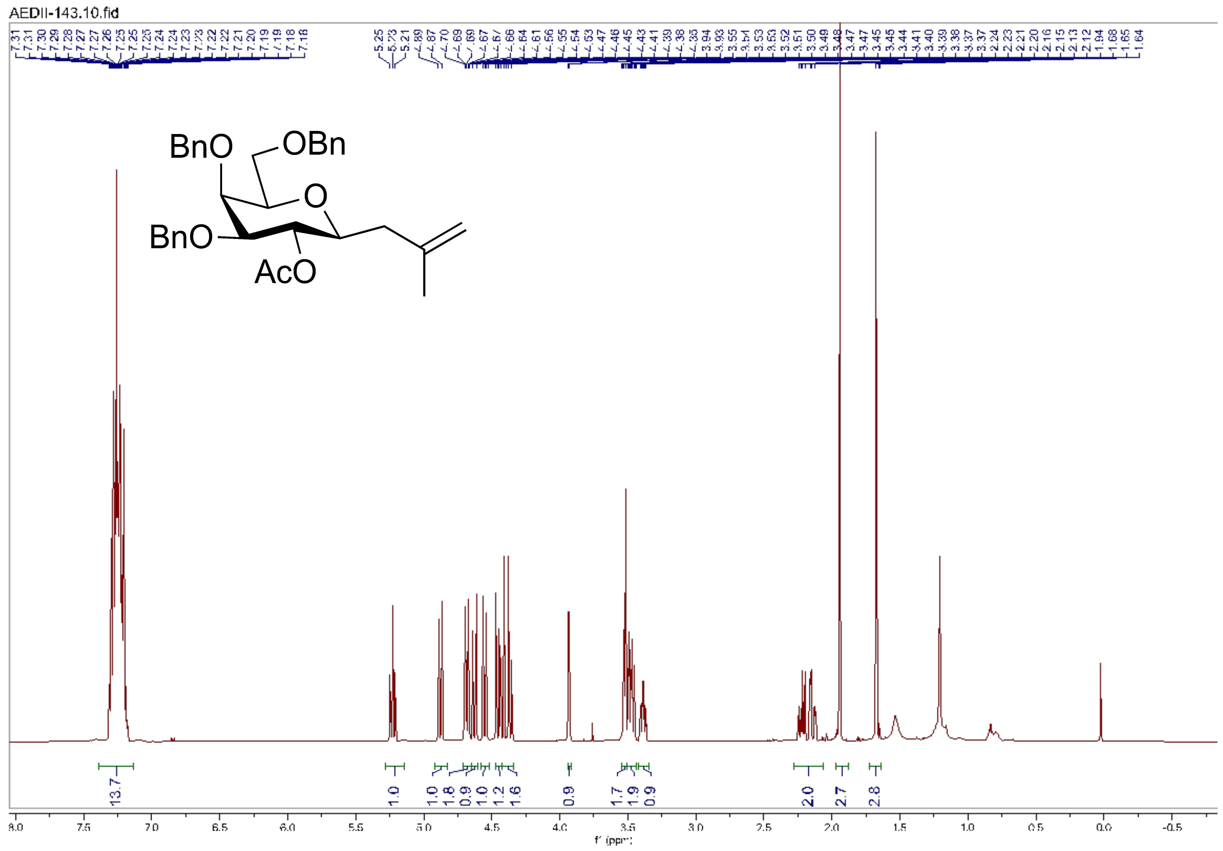

^{1}H NMR (500 MHz, $CDCl_3$) spectrum of compound **10**

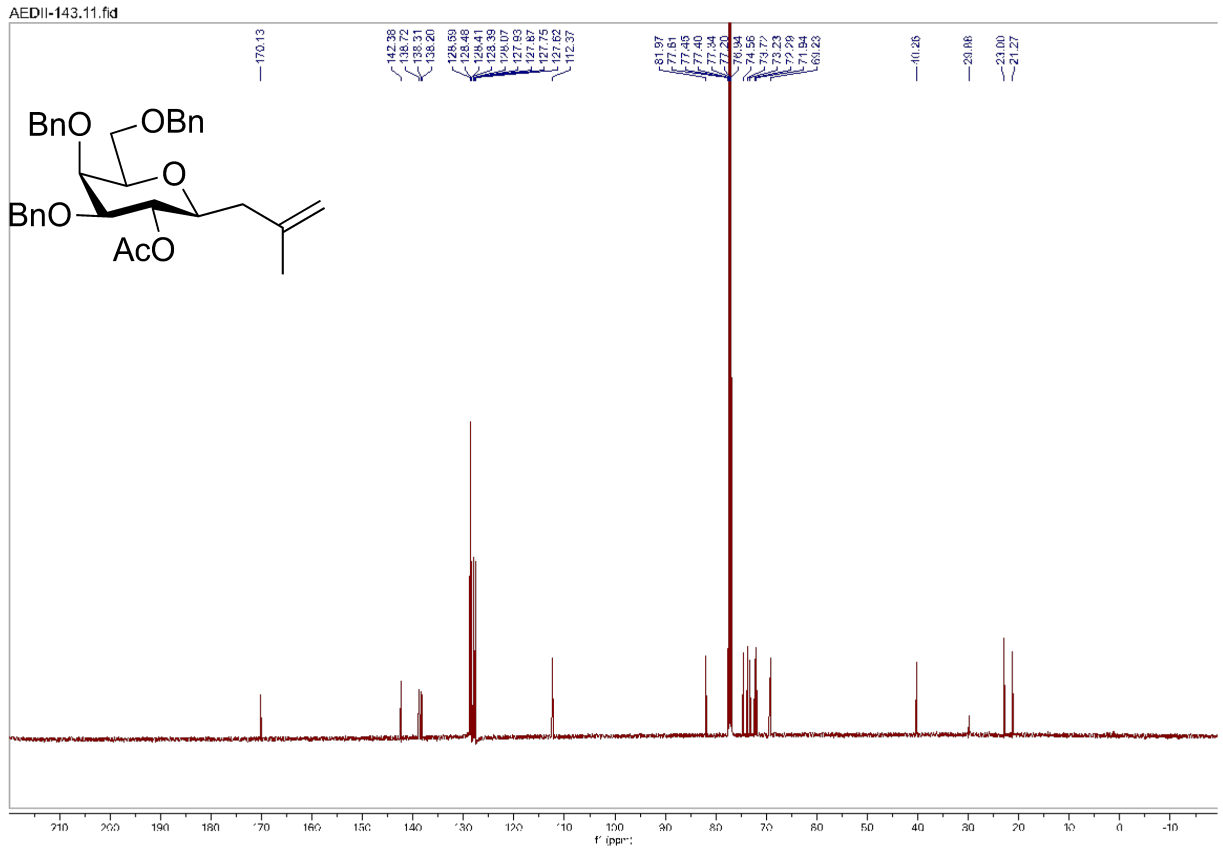

^{13}C NMR (126 MHz, $CDCl_3$) spectrum of compound **10**

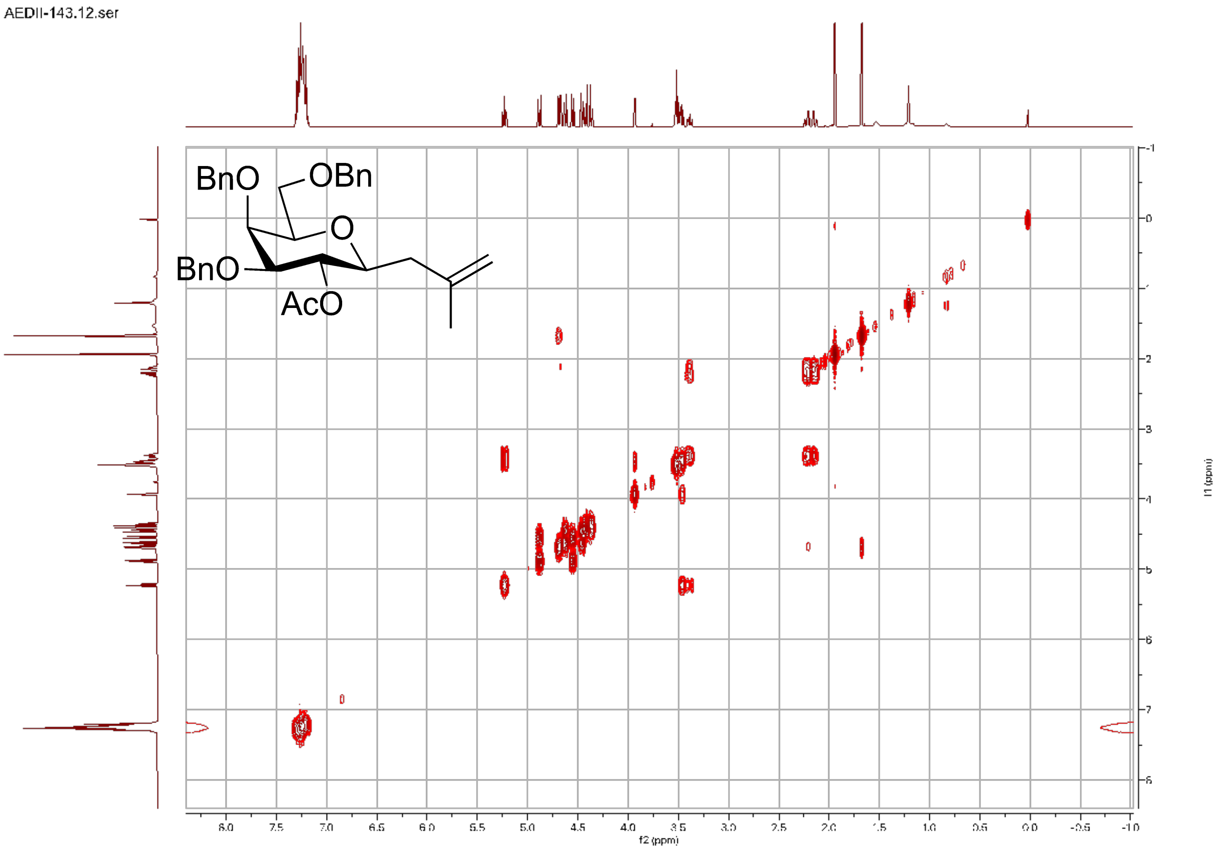

COSY (500 MHz, $CDCl_3$) spectrum of compound **10**

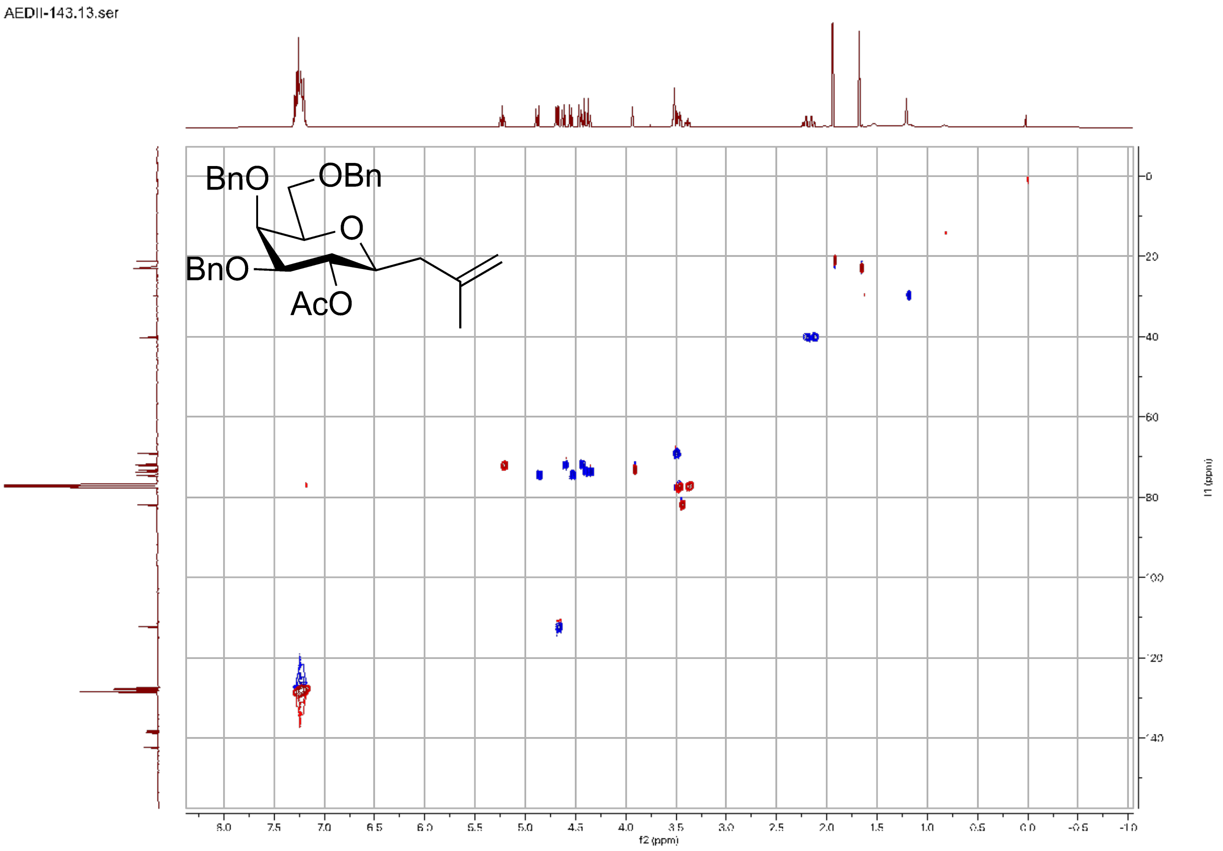

HSQC (500 MHz, 126 MHz, $CDCl_3$) spectrum of compound **10**

AEDII-143.14.ser

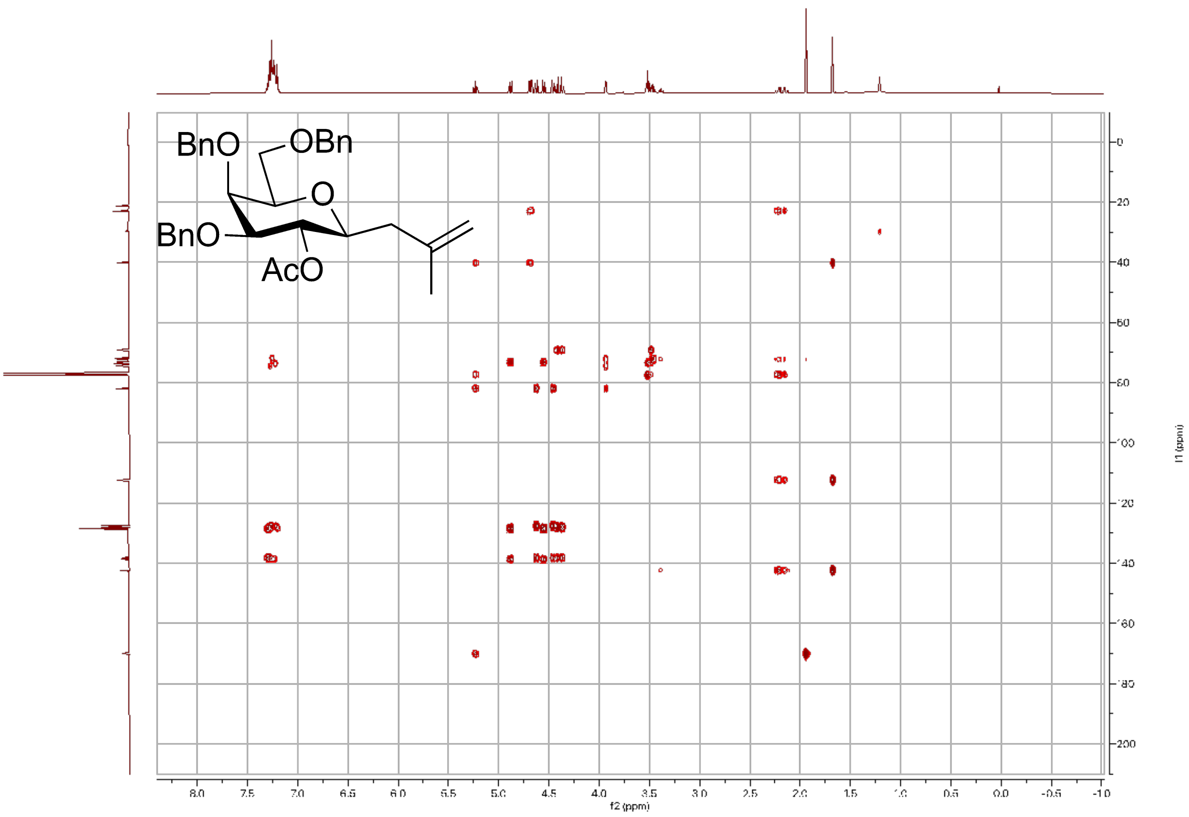

HMBC (500 MHz, 126 MHz, CDCl3) spectrum of compound **10**

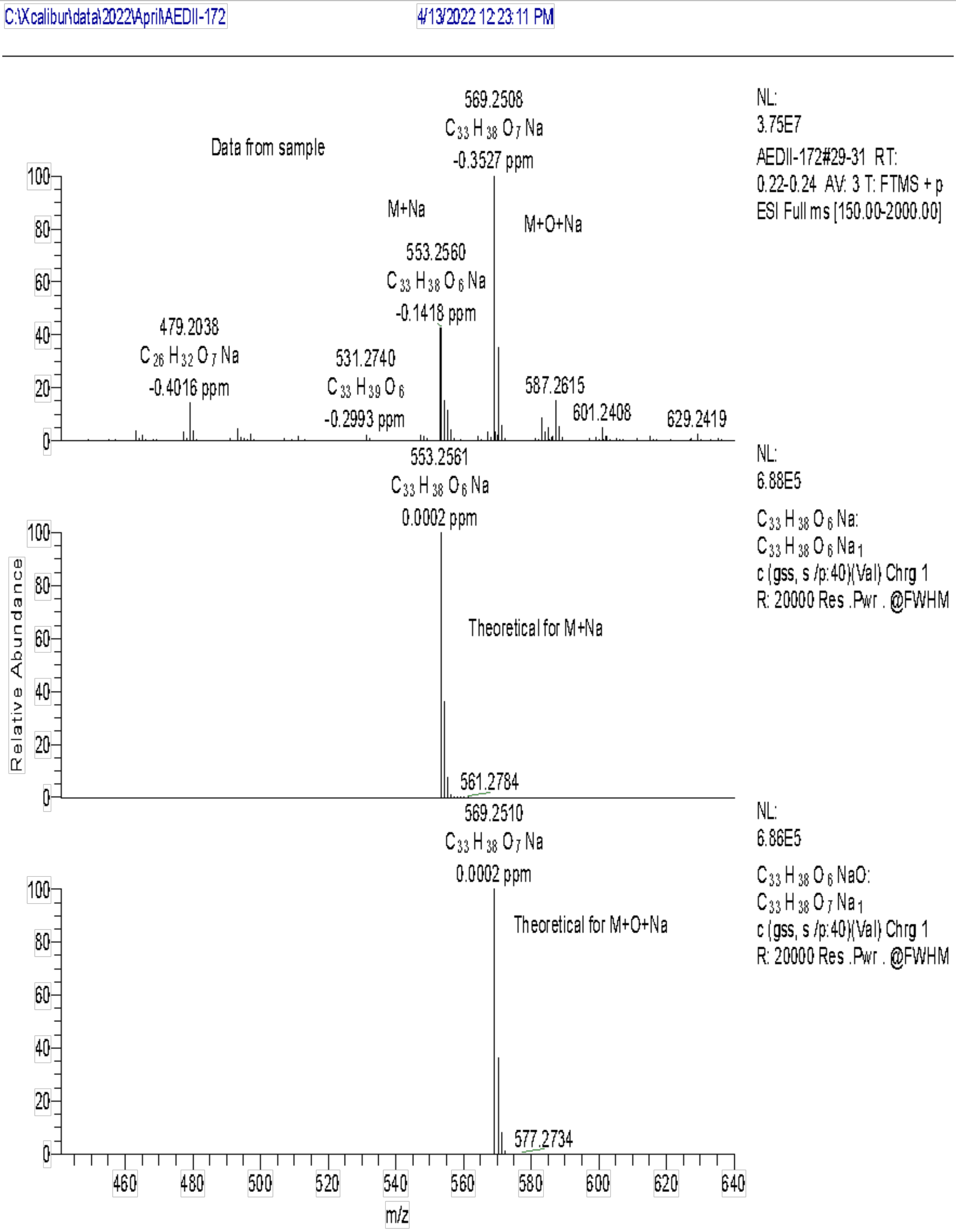

HRMS of compound **10**

SUPPORTING INFORMATION

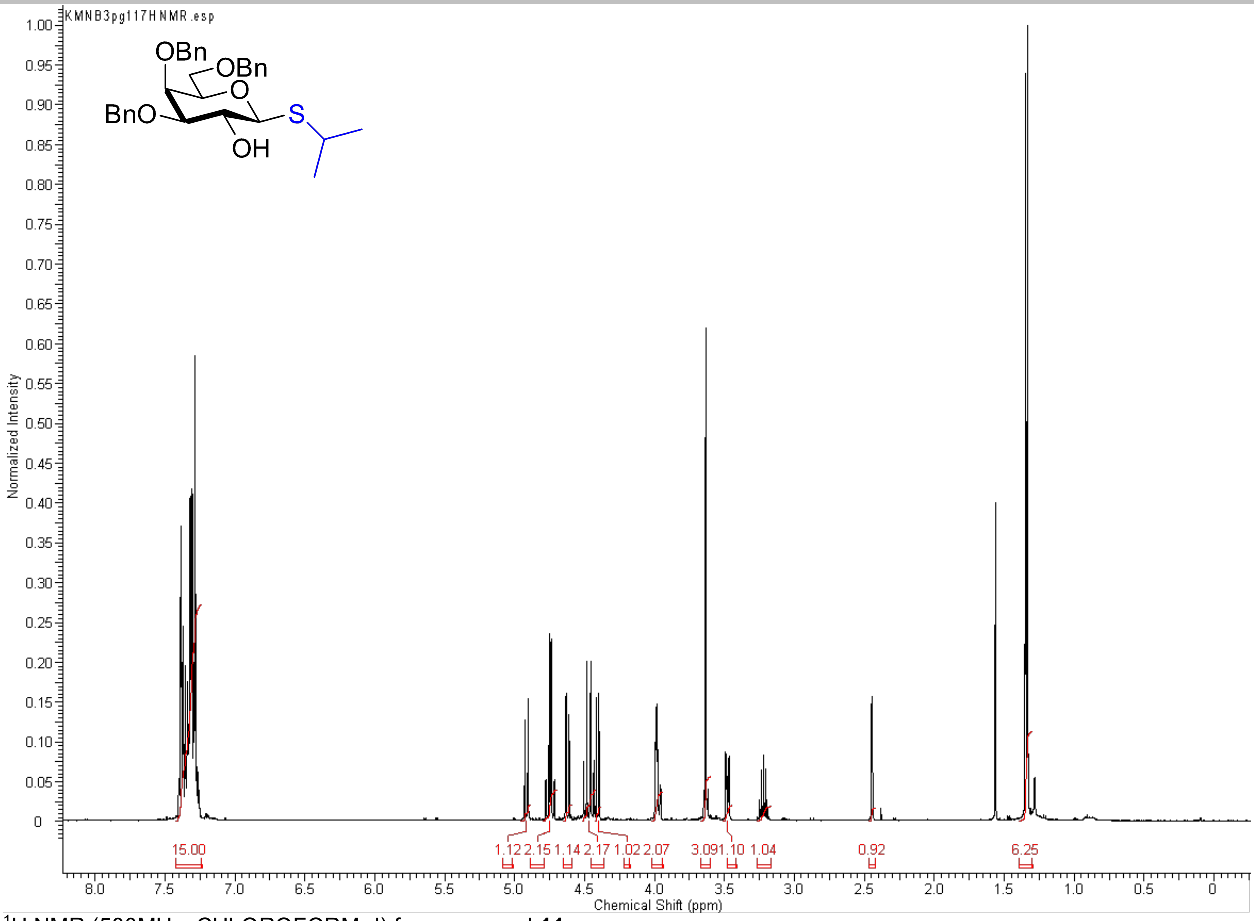

^{1}H NMR (500MHz ,CHLOROFORM-d) for compound **11**

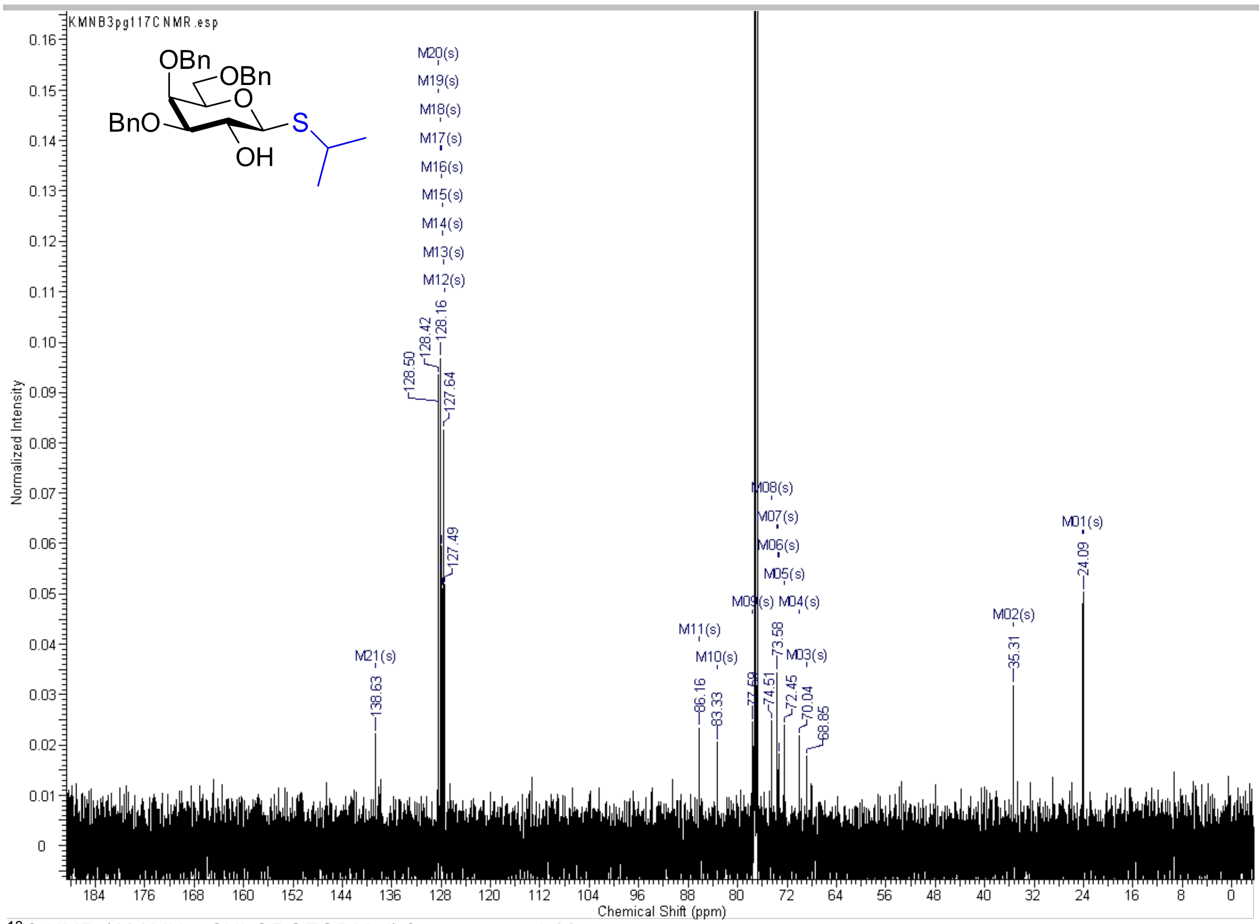

^{13}C NMR (126MHz ,CHLOROFORM-d) for compound **11**

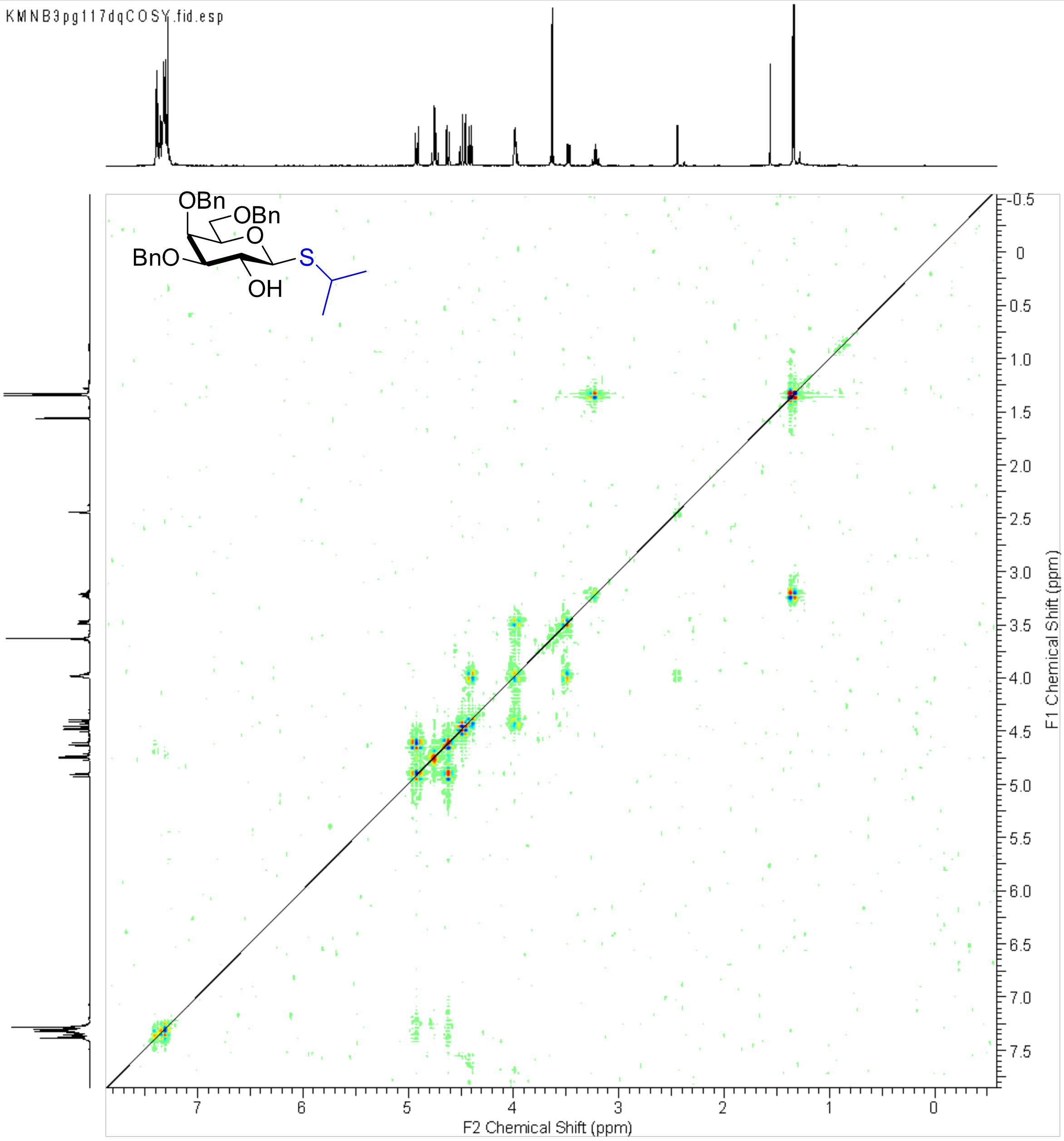

dqCOSY (500MHz ,CHLOROFORM-d) for compound **11**

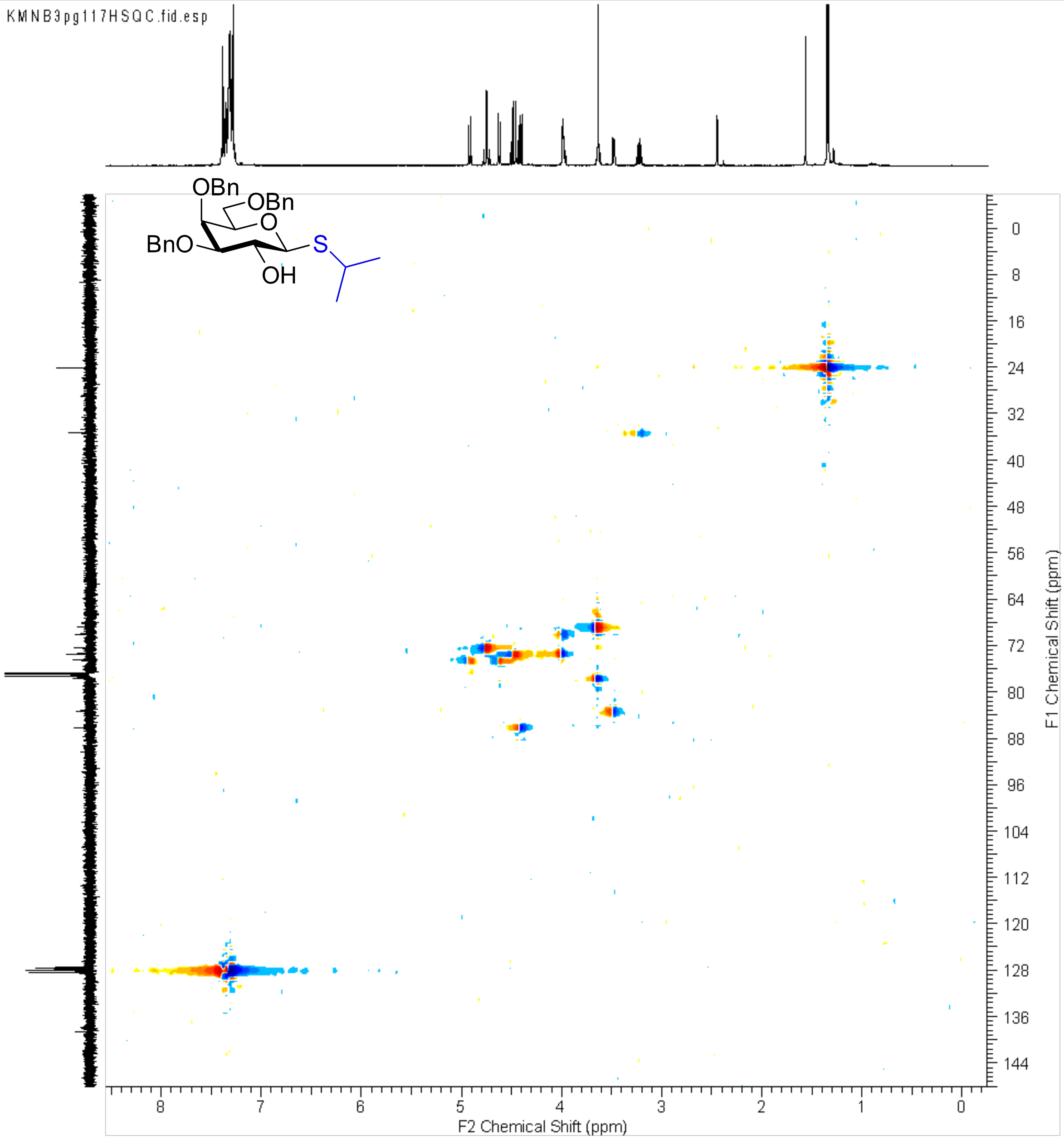

HSQC (500MHz ,CHLOROFORM-d) for compound **11**

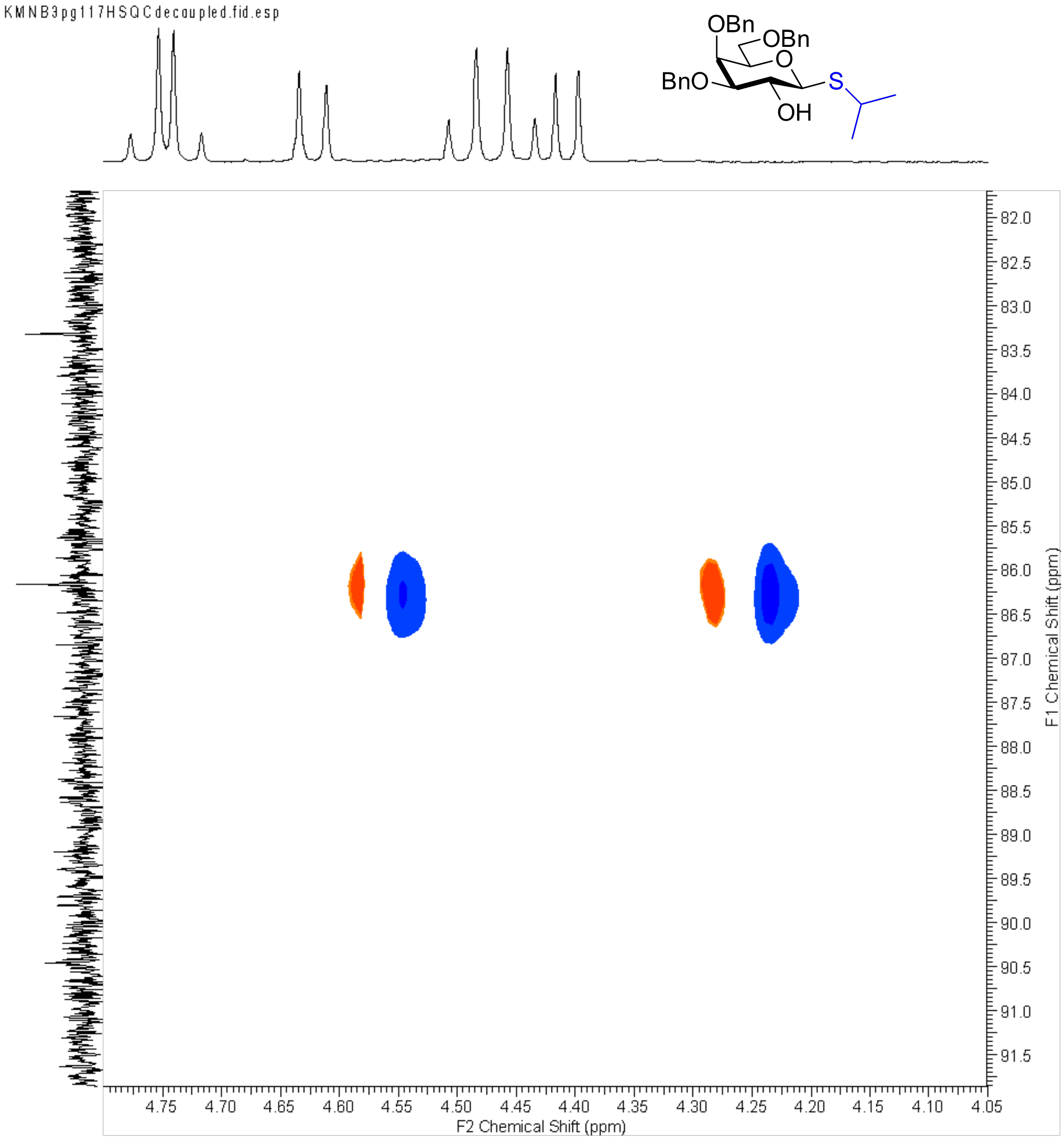

HSQC coupled (500MHz ,CHLOROFORM-d) for compound **11**
$J_{H-1,C-1}$ = 155.5 Hz meaning the anomeric proton is axial.

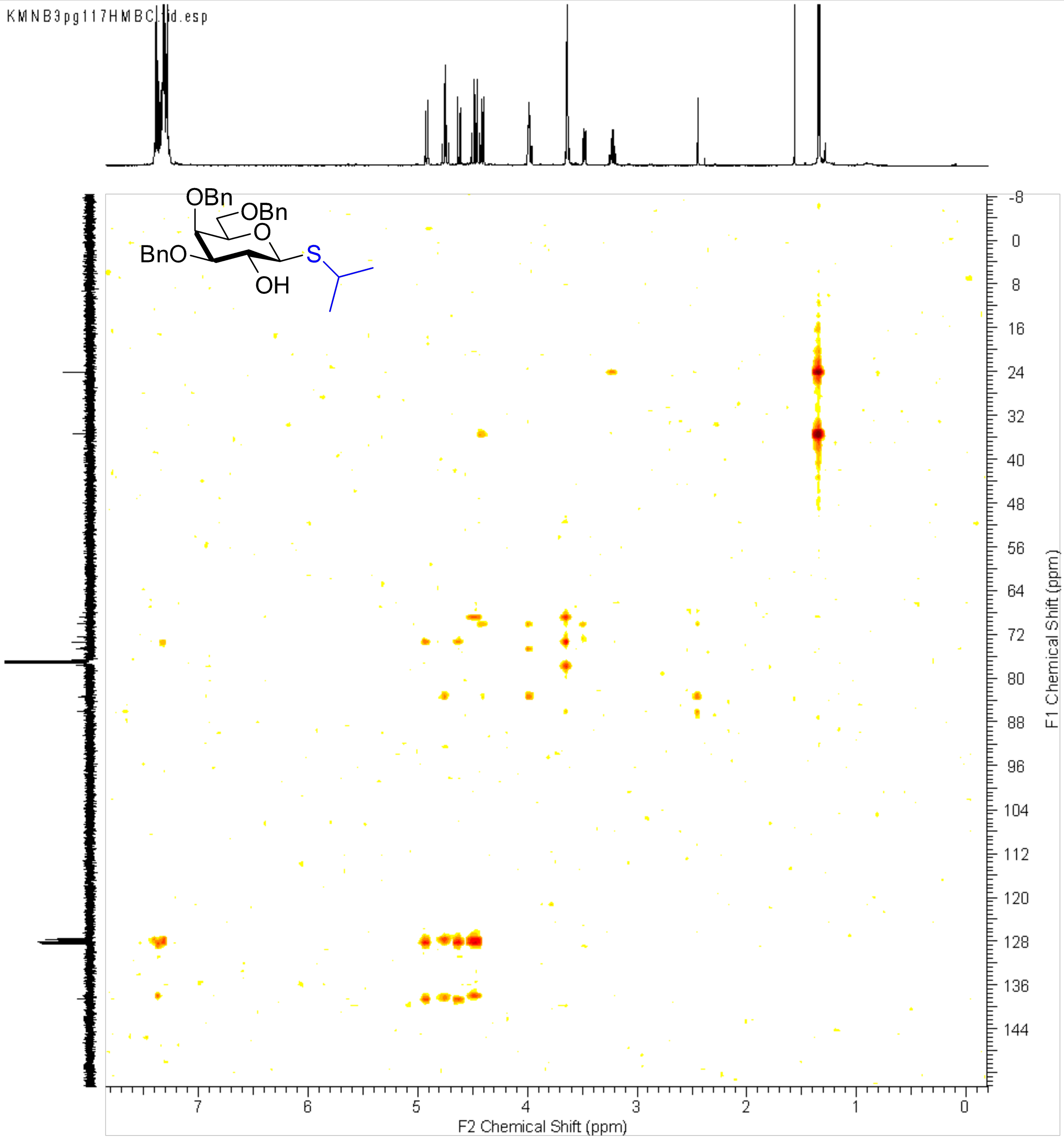

HMBC (500MHz ,CHLOROFORM-d) for compound **11**

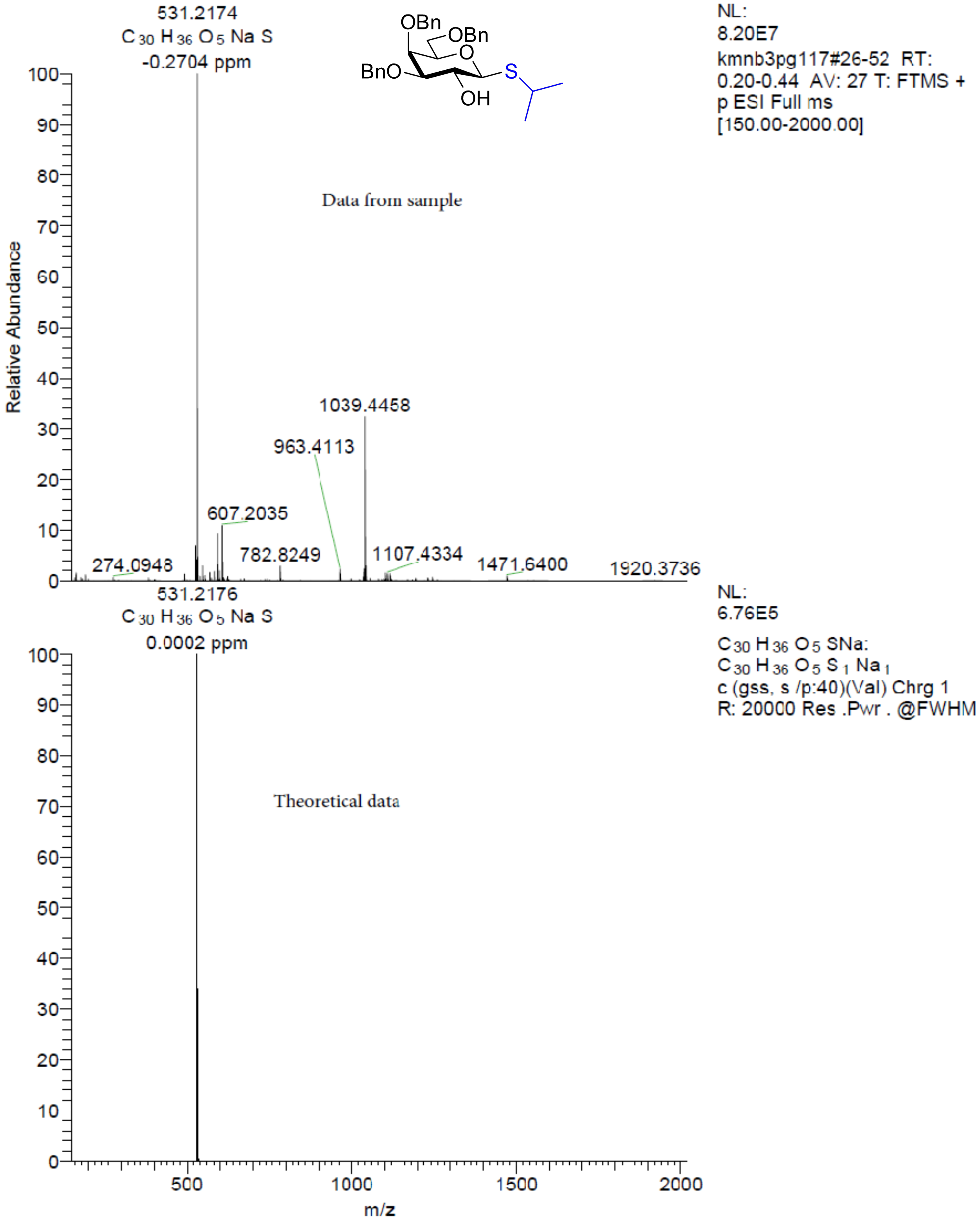

HRMS for compound **11**

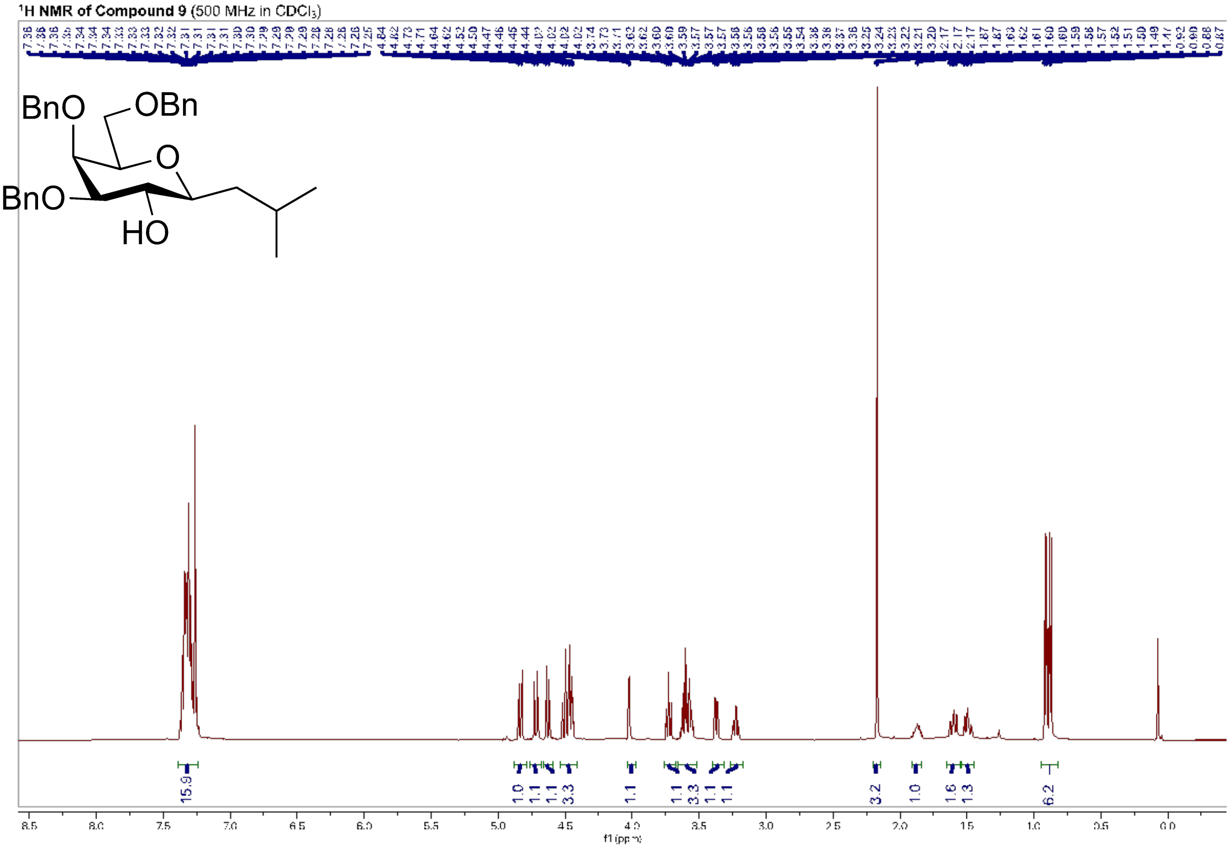

^{1}H NMR (500 MHz, CDCl$_3$) spectrum of compound **12**

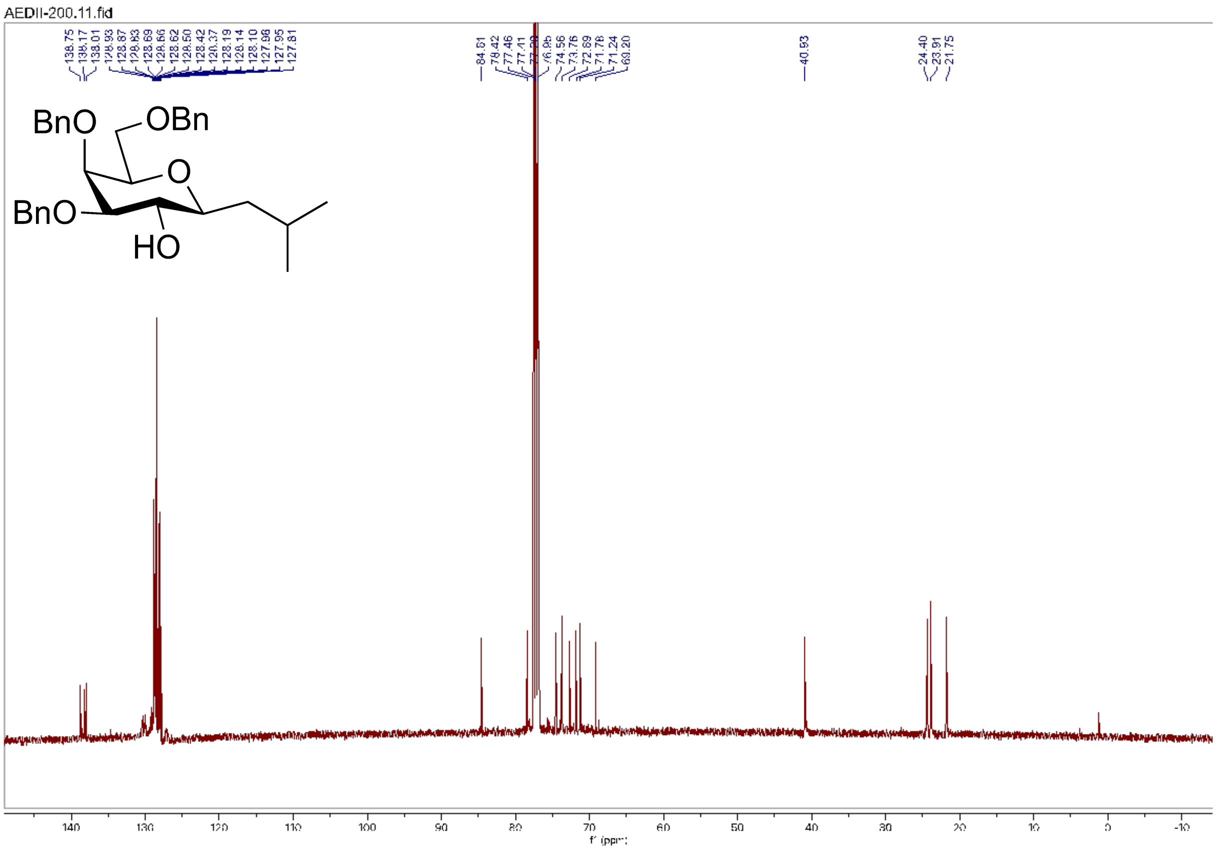

^{13}C NMR (126 MHz, $CDCl_3$) spectrum of compound **12**

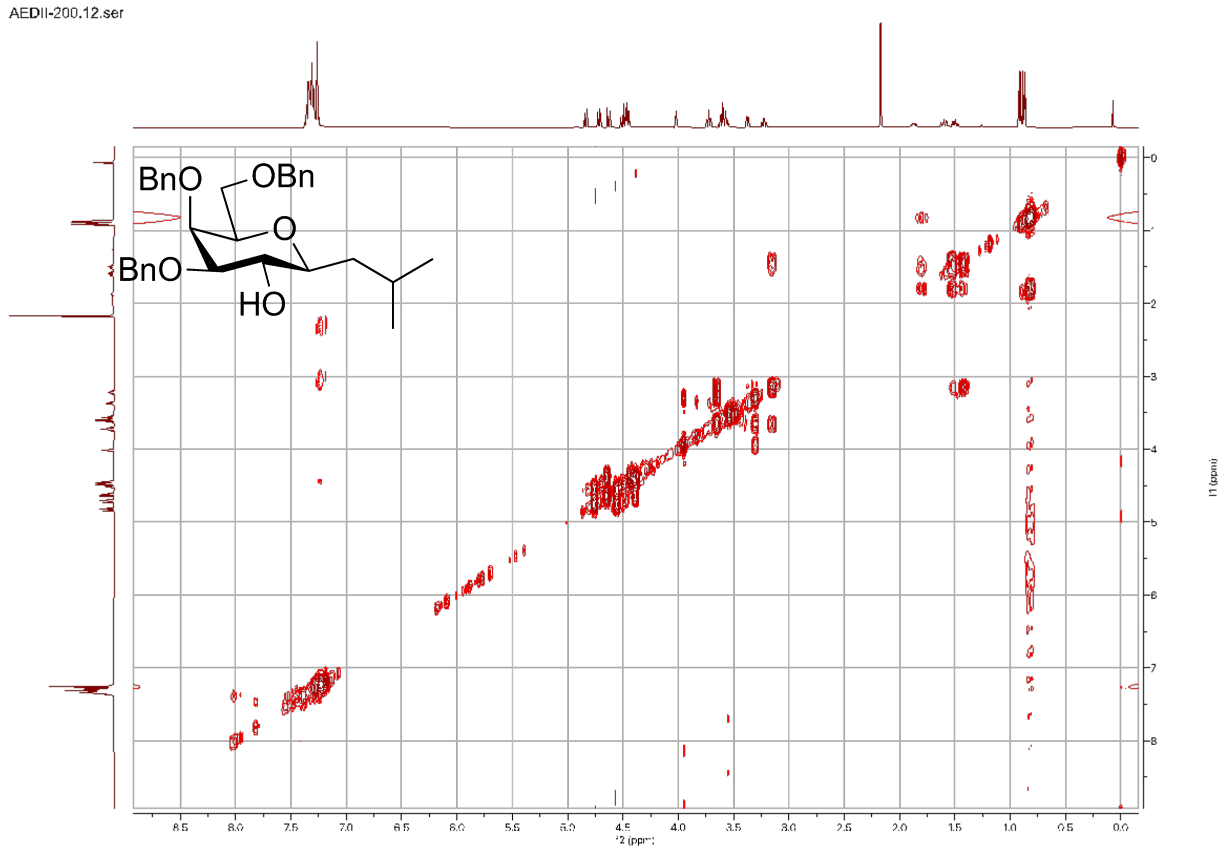

COSY (500 MHz, $CDCl_3$) spectrum of compound **12**

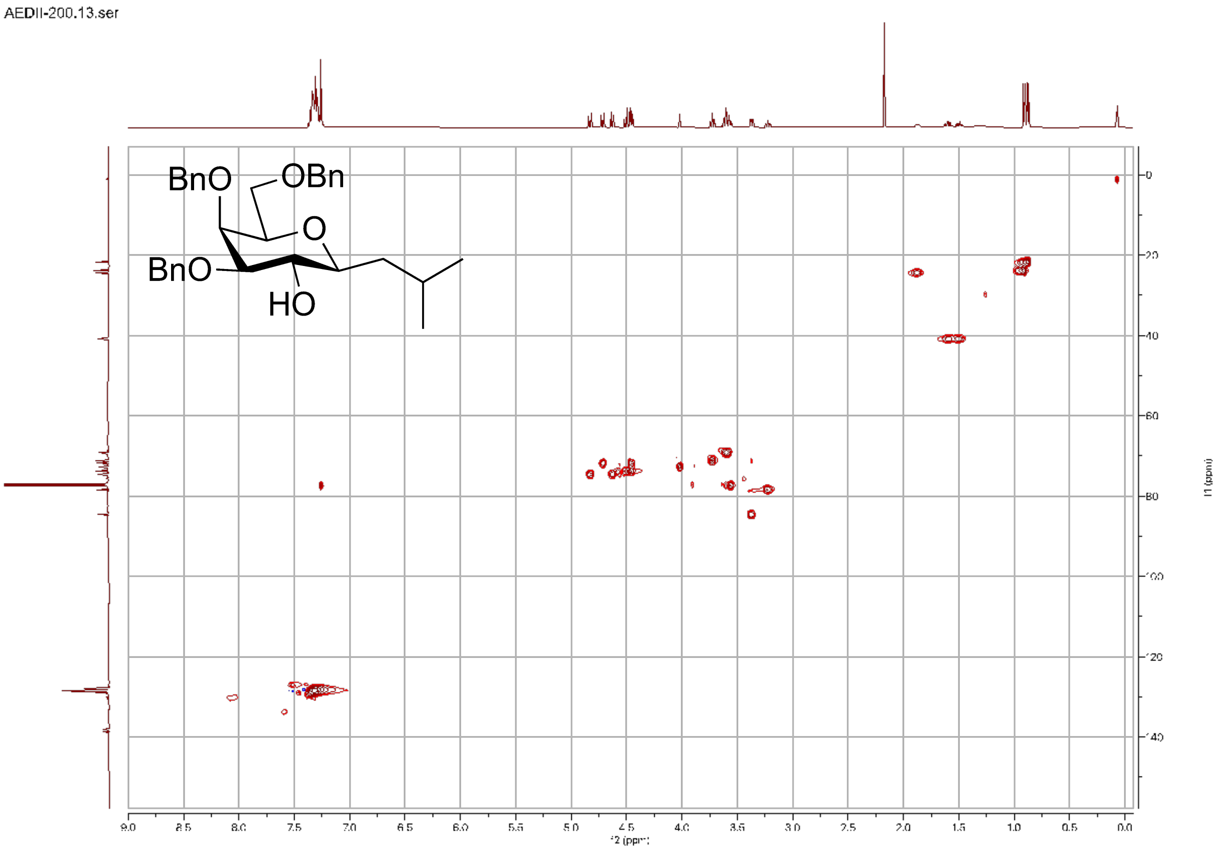

HSQC (500 MHz, 126 MHz, $CDCl_3$) spectrum of compound **12**

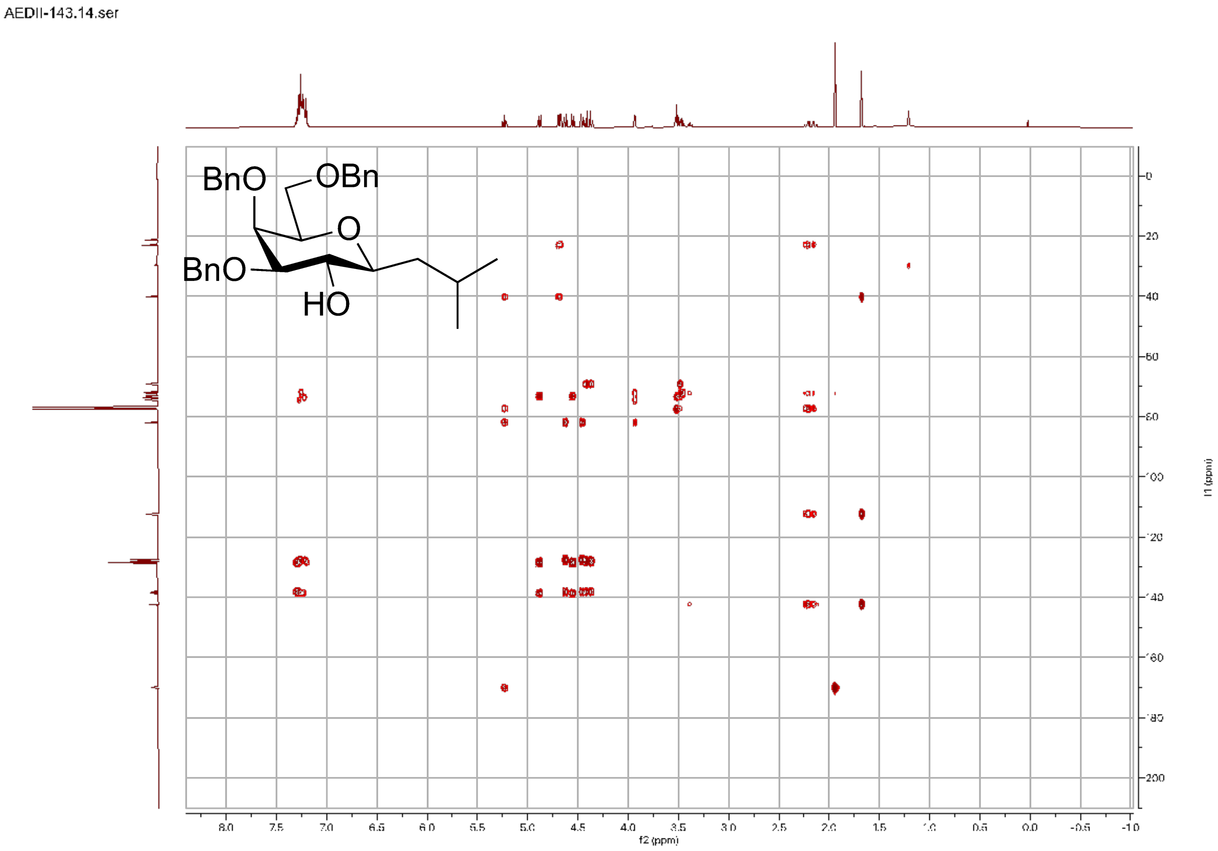

HMBC (500 MHz, 126 MHz, $CDCl_3$) spectrum of compound **12**

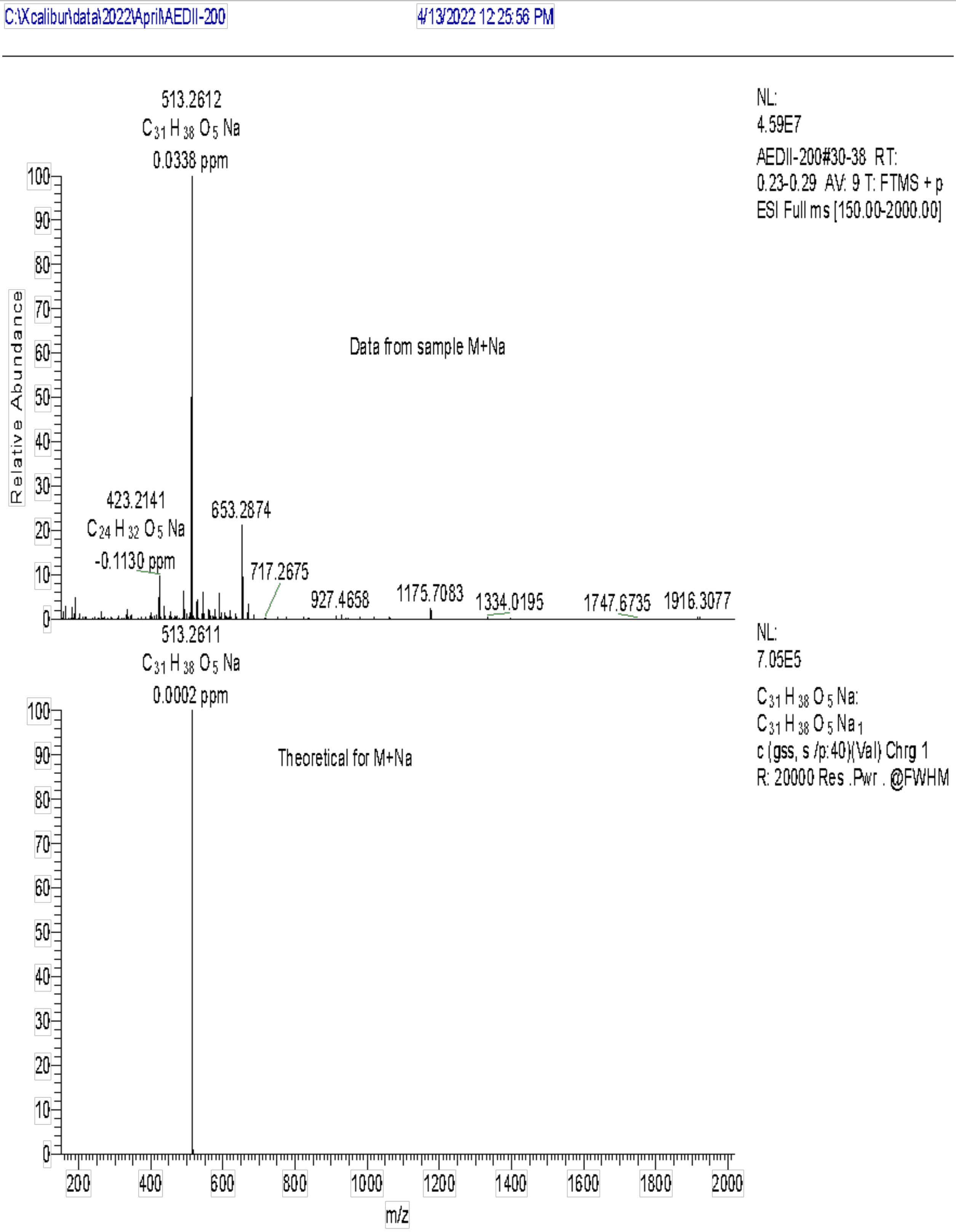

HRMS of compound **12**

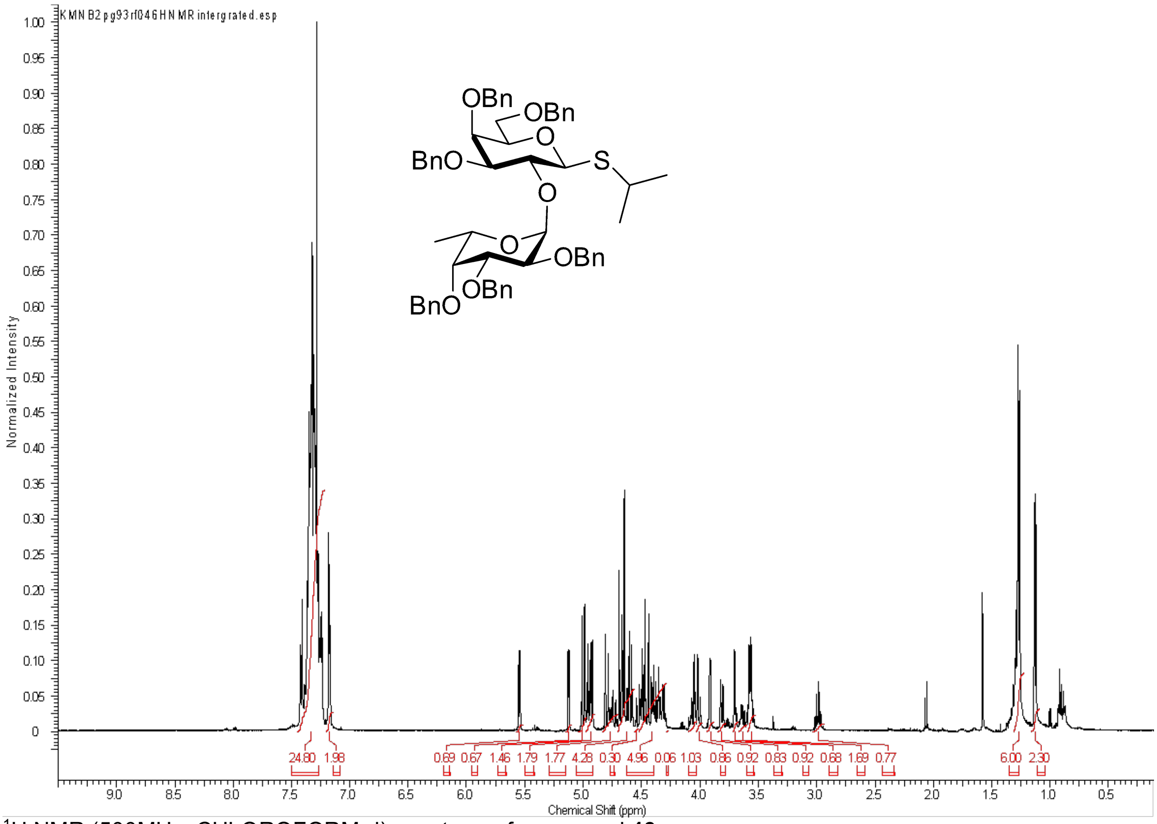

^{1}H NMR (500MHz ,CHLOROFORM-d) spectrum of compound **13**

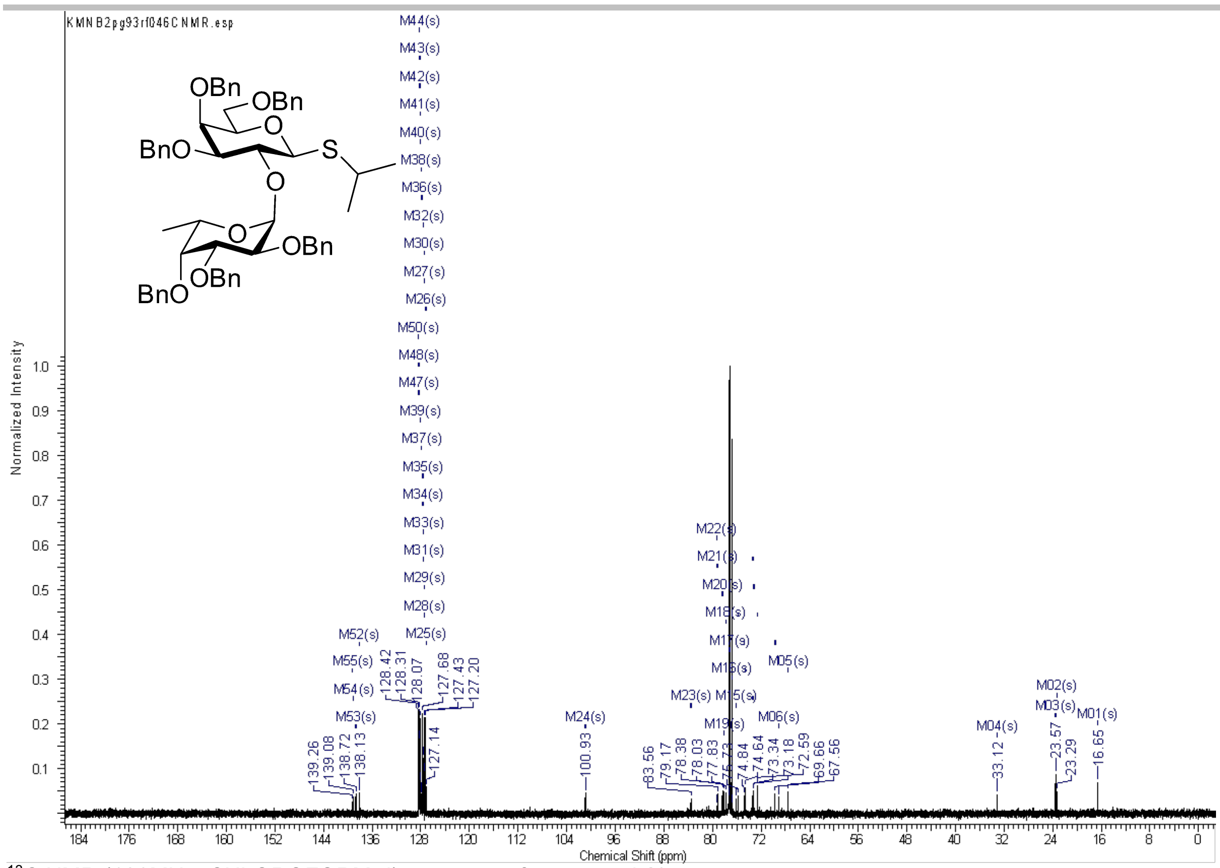

^{13}C NMR (126MHz ,CHLOROFORM-d) spectrum of compound **13**

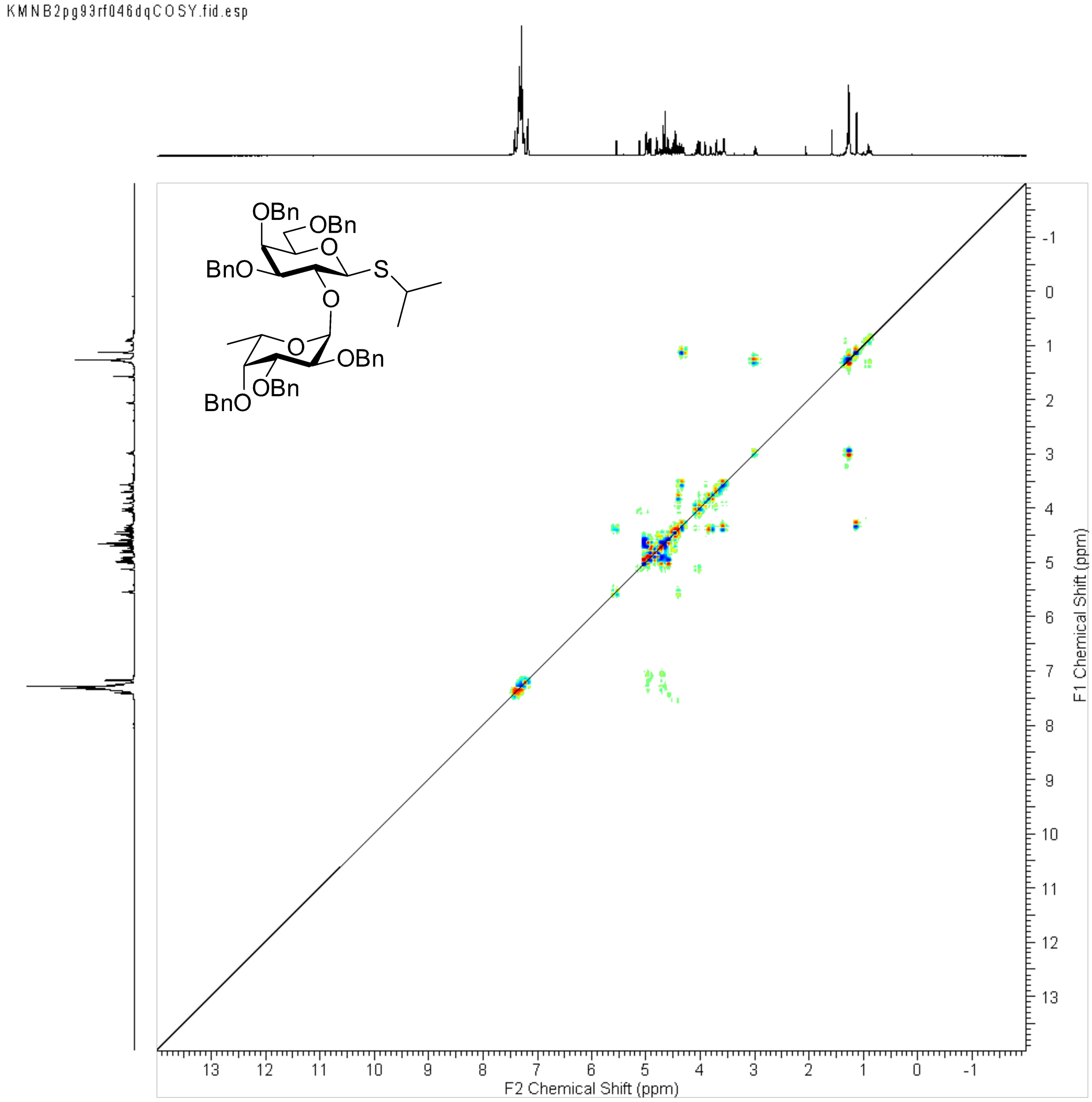

dqCOSY (500MHz ,CHLOROFORM-d) spectrum of compound **13**

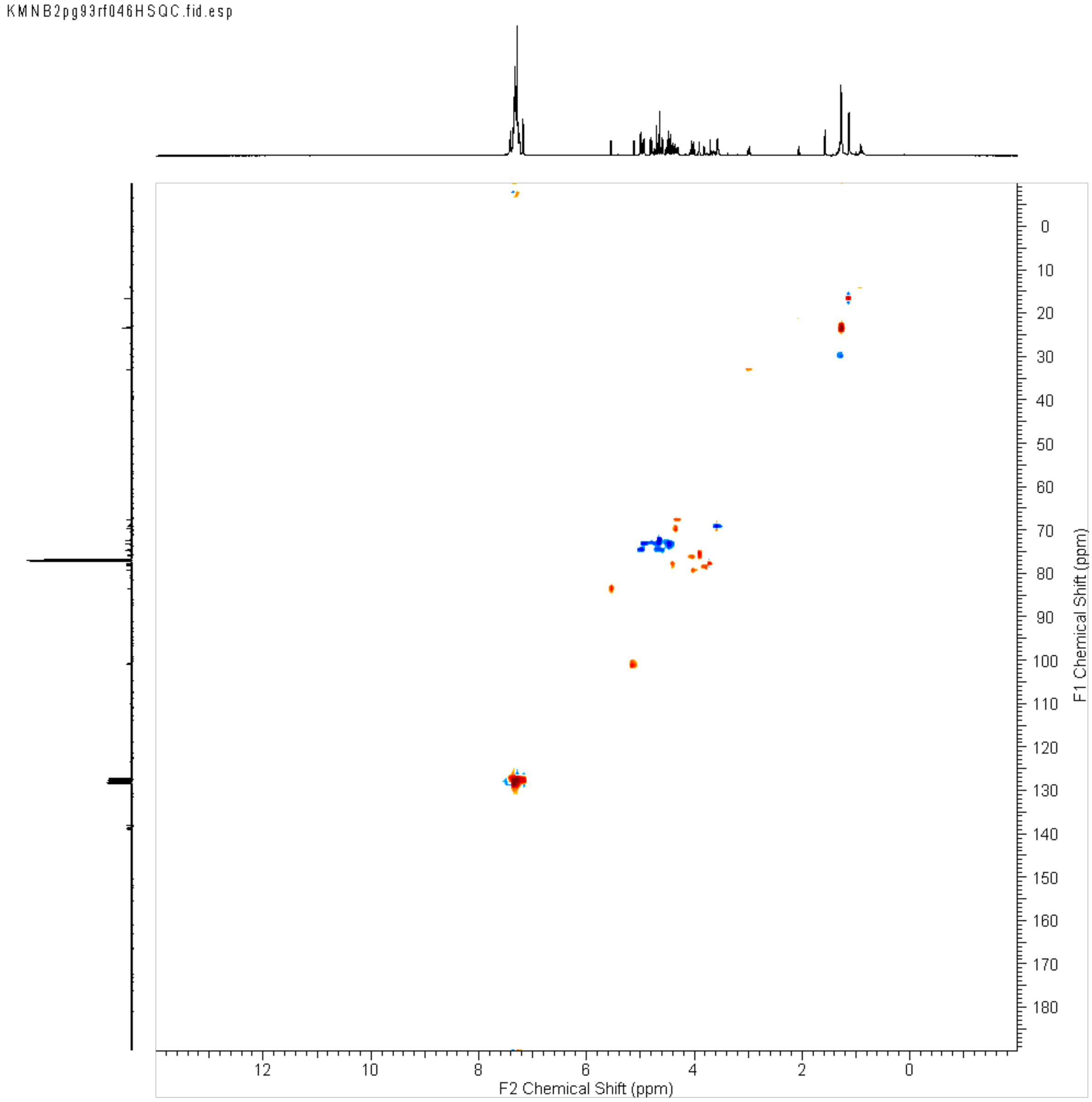

HSQC (500MHz ,CHLOROFORM-d) spectrum of compound **13**

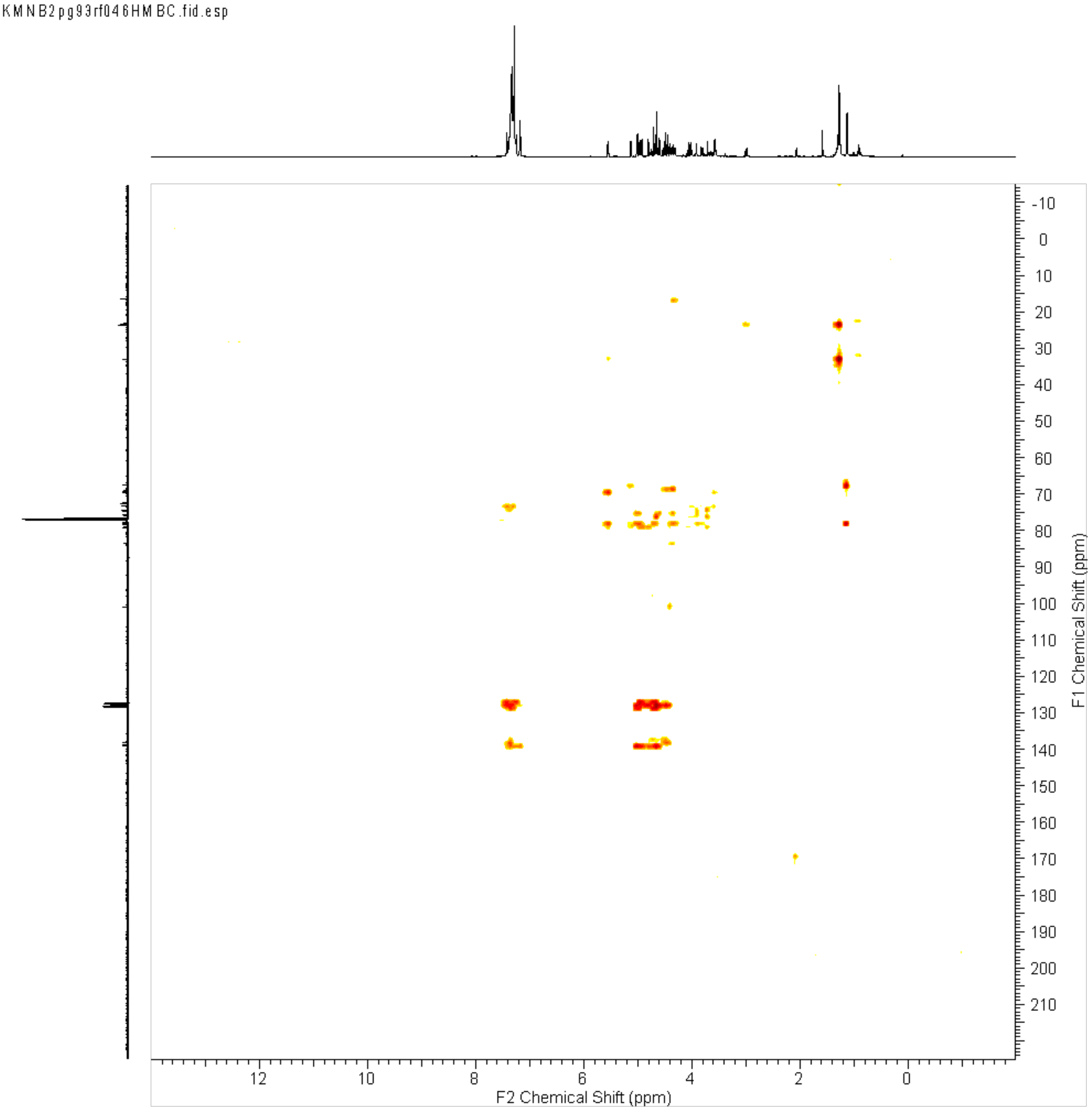

HMBC (500MHz ,CHLOROFORM-d) spectrum of compound **13**

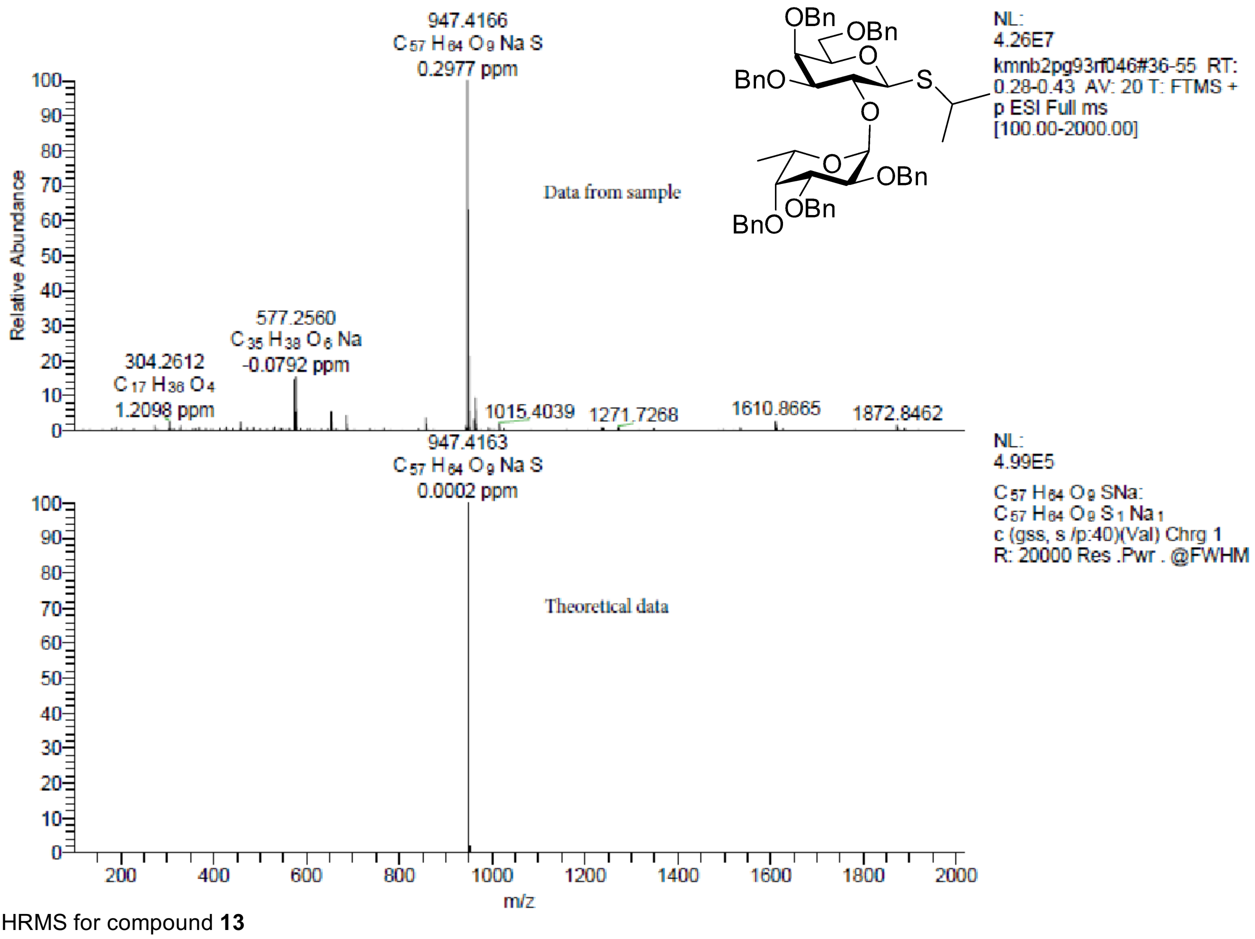

HRMS for compound **13**

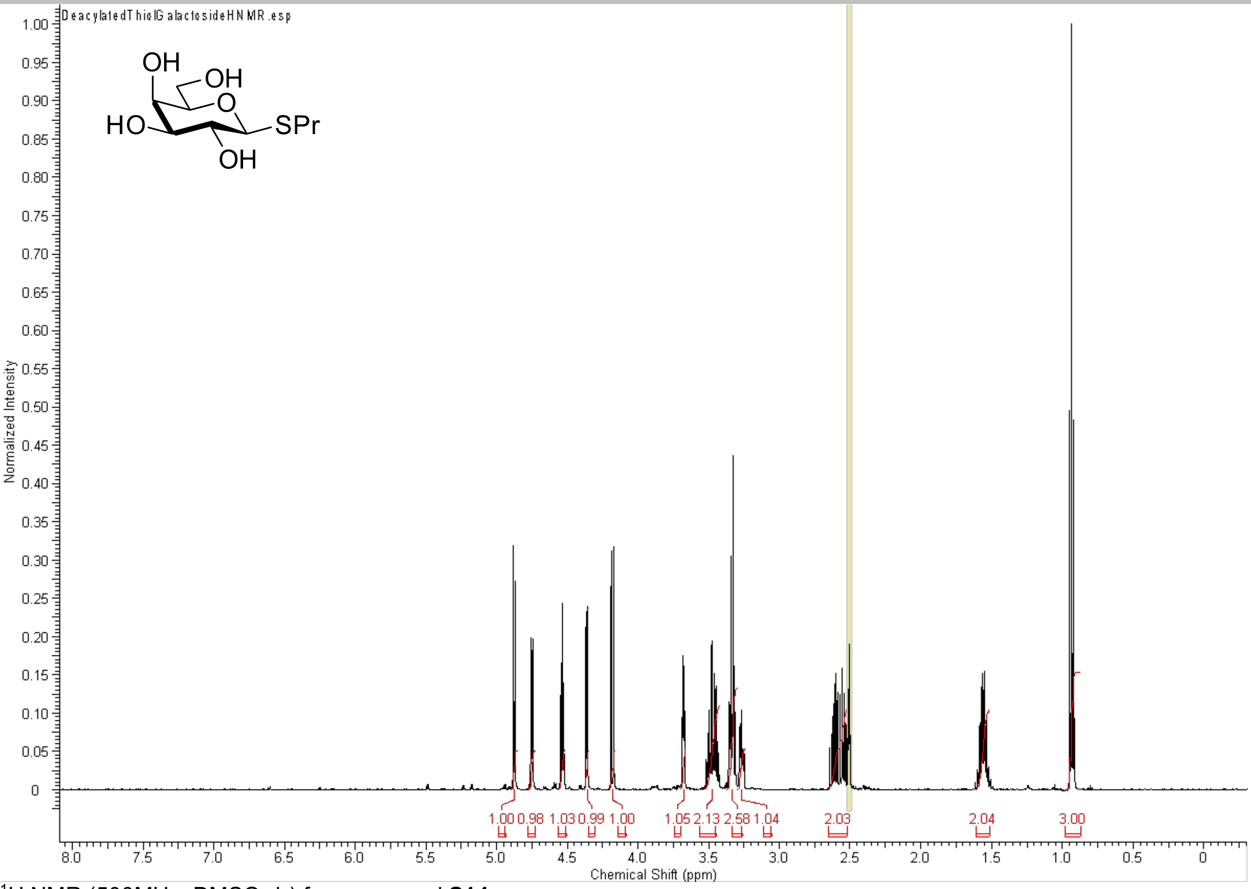

^{1}H NMR (500MHz ,DMSO-d6) for compound **S14**

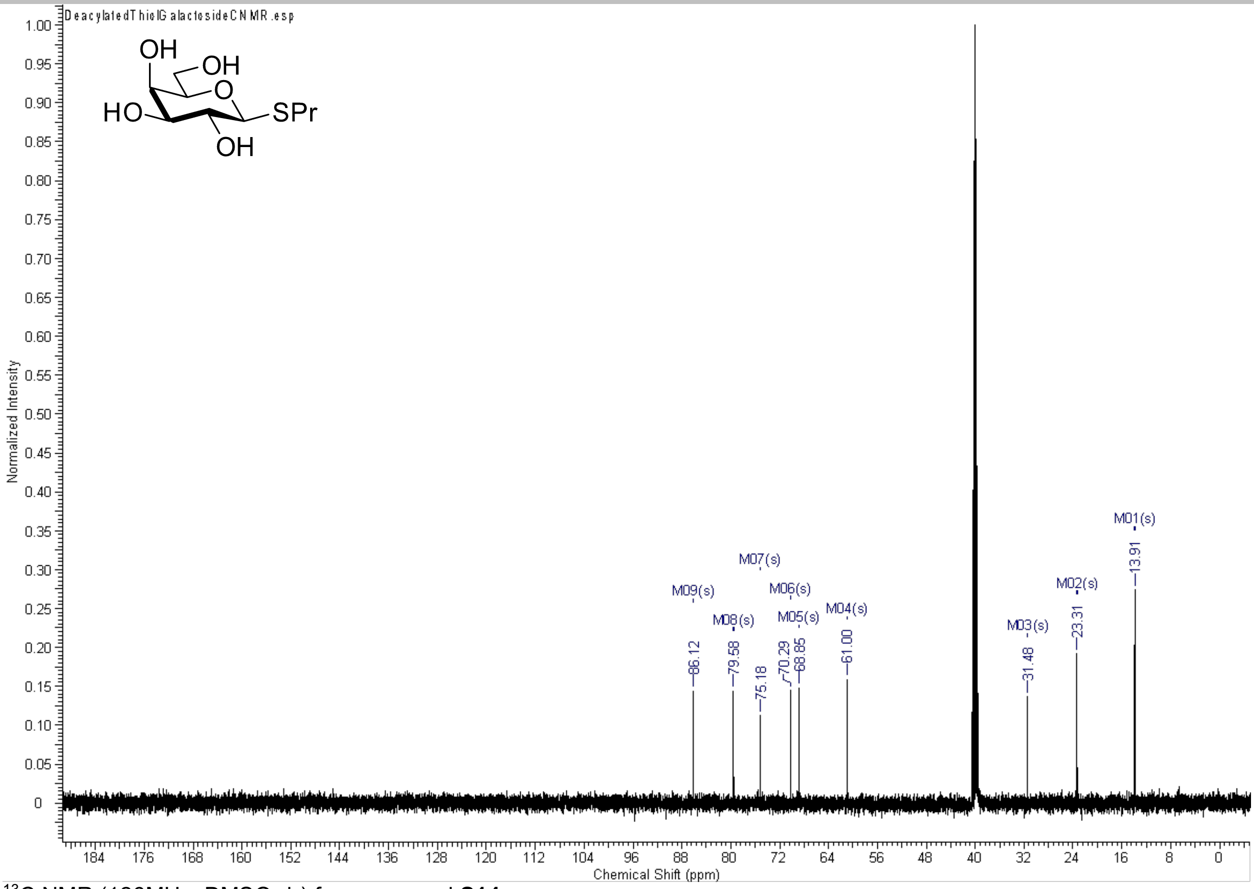

^{13}C NMR (126MHz ,DMSO-d_6) for compound **S14**

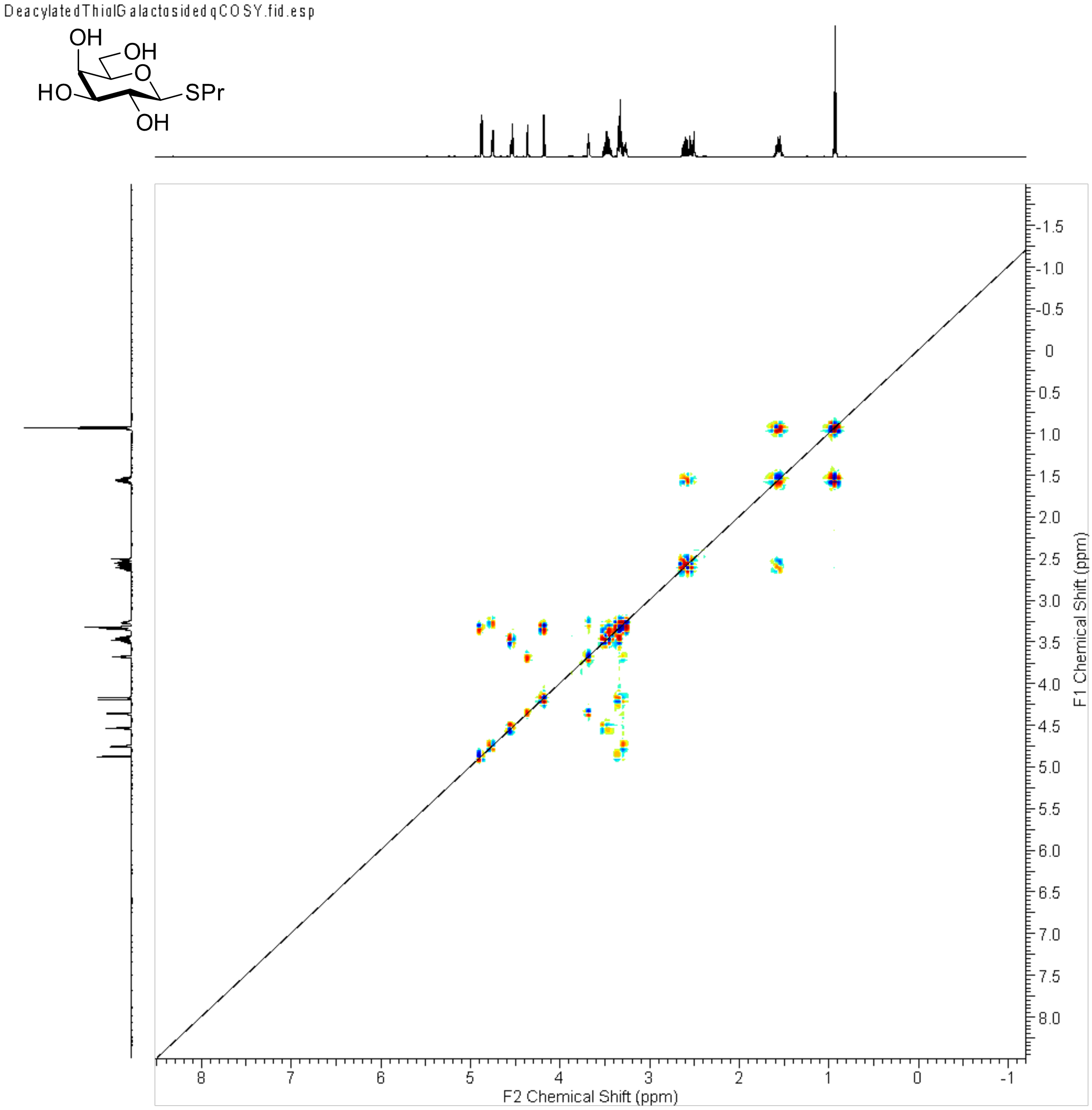

COSY (500MHz ,DMSO-d_6) for compound **S14**

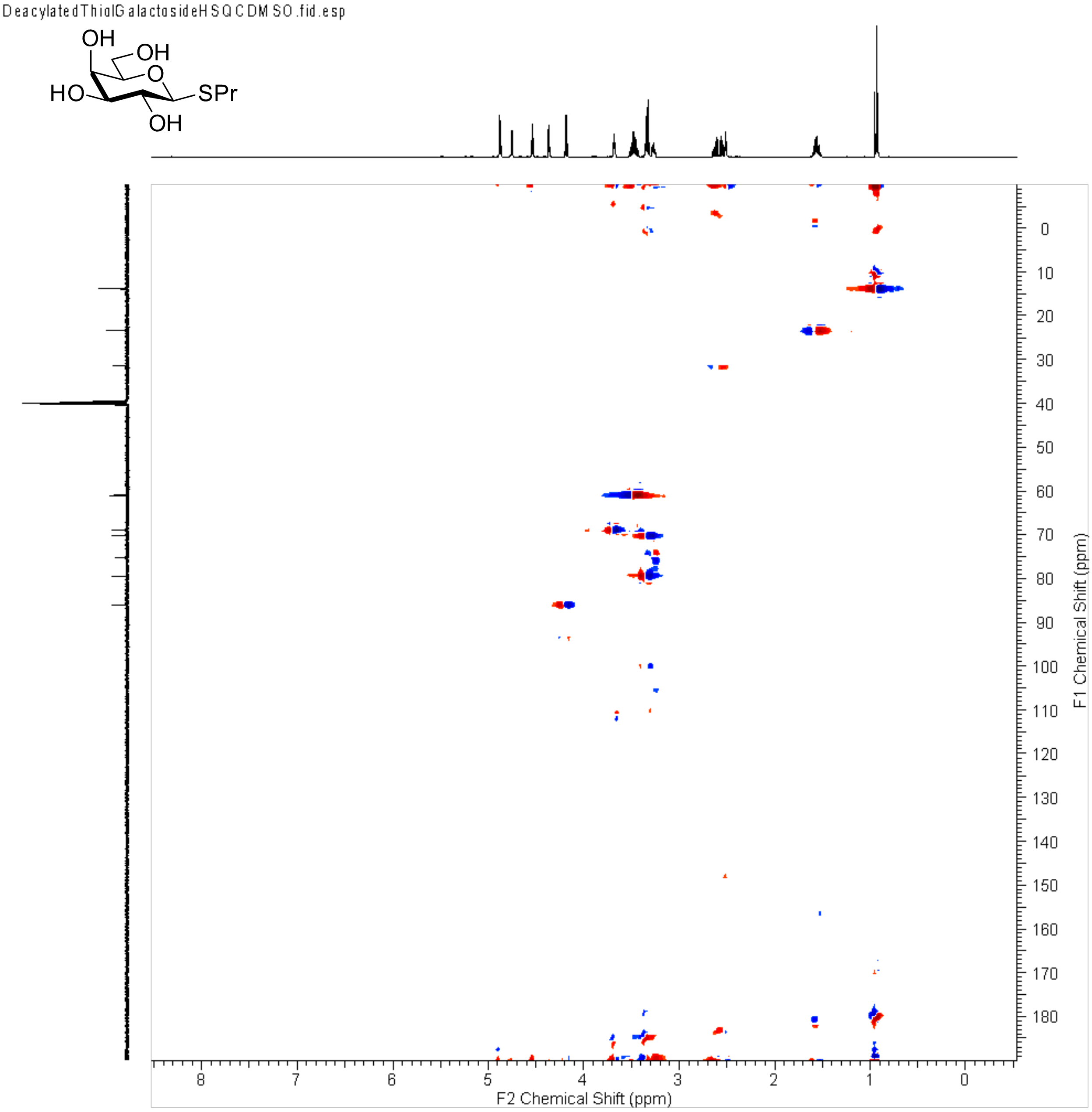

HSQC (500MHz ,DMSO-d_6) for compound **S14**

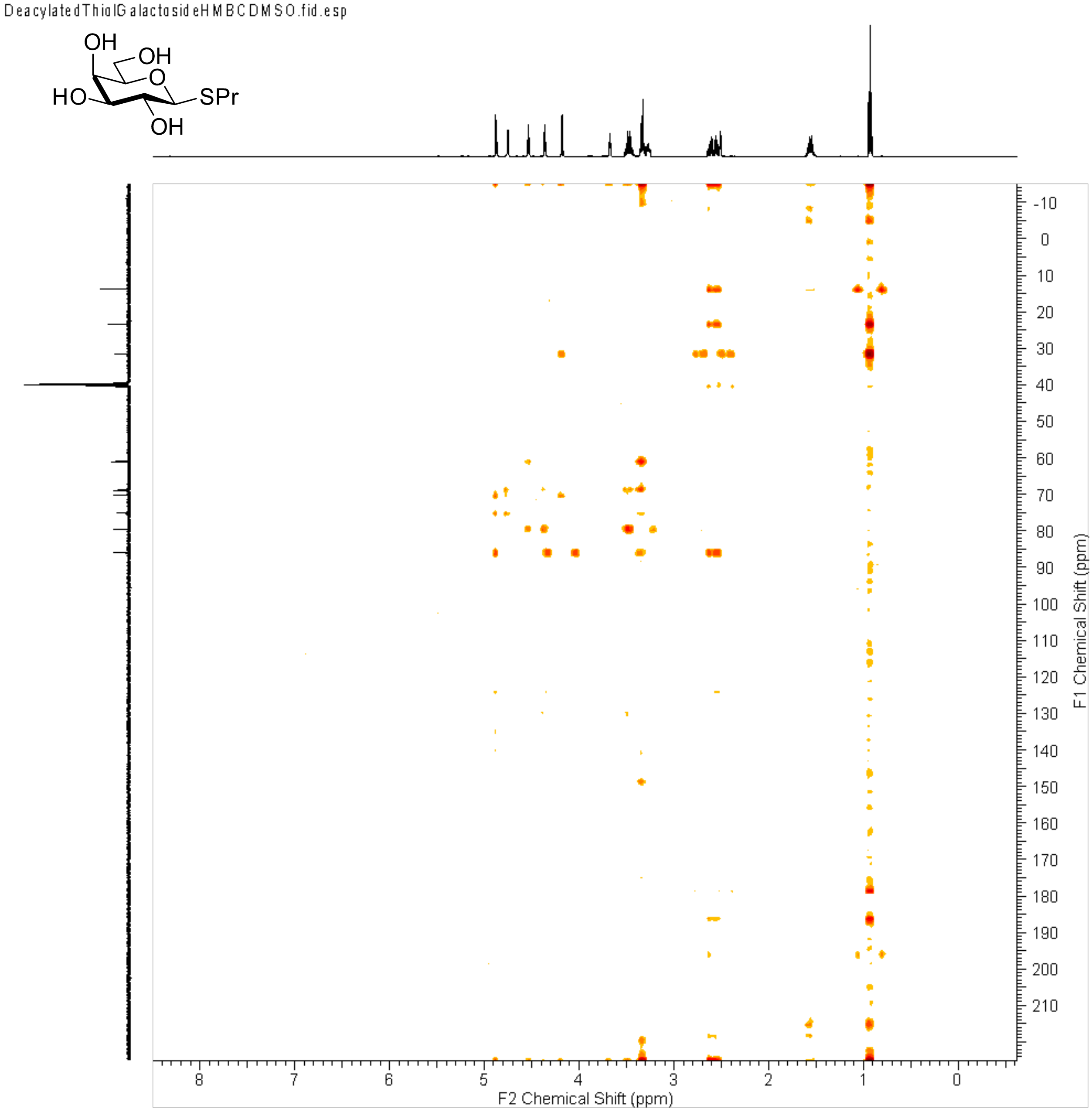

HMBC (500MHz ,DMSO-d_6) for compound **S14**

Elemental Composition Report **Page 1**

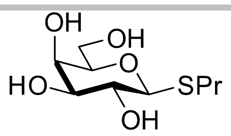

Multiple Mass Analysis: 2 mass(es) processed
Tolerance = 50.0 PPM / DBE: min = -1.5, max = 100.0
Element prediction: Off
Number of isotope peaks used for i-FIT = 3

Monoisotopic Mass, Even Electron Ions
47 formula(e) evaluated with 1 results within limits (up to 50 closest results for each mass)
Elements Used:
C: 0-9 H: 0-18 O: 0-5 Na: 0-1 S: 0-1
deacylated-thiol-galactoside
deacylated-thiol-galactosidee 42 (0.790) AM2 (Ar,10000.0,430.91,1.00,LS 4); ABS; Cm (41:44) 1: TOF MS ES+ 7.35e+003

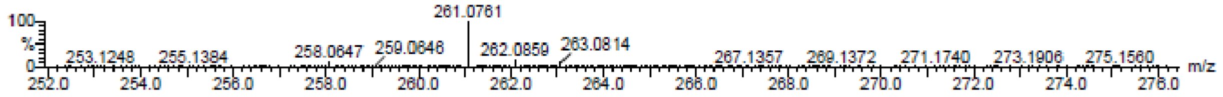

Minimum:	15.00				-1.5			
Maximum:	100.00		5.0	50.0	100.0			

Mass	RA	Calc. Mass	mDa	PPM	DBE	i-FIT	i-FIT (Norm)	Formula
261.0761	100.00	261.0773	-1.2	-4.6	0.5	246.7	0.0	C9 H18 O5 Na S
262.0859	15.12	---						

HRMS for compound **S14**

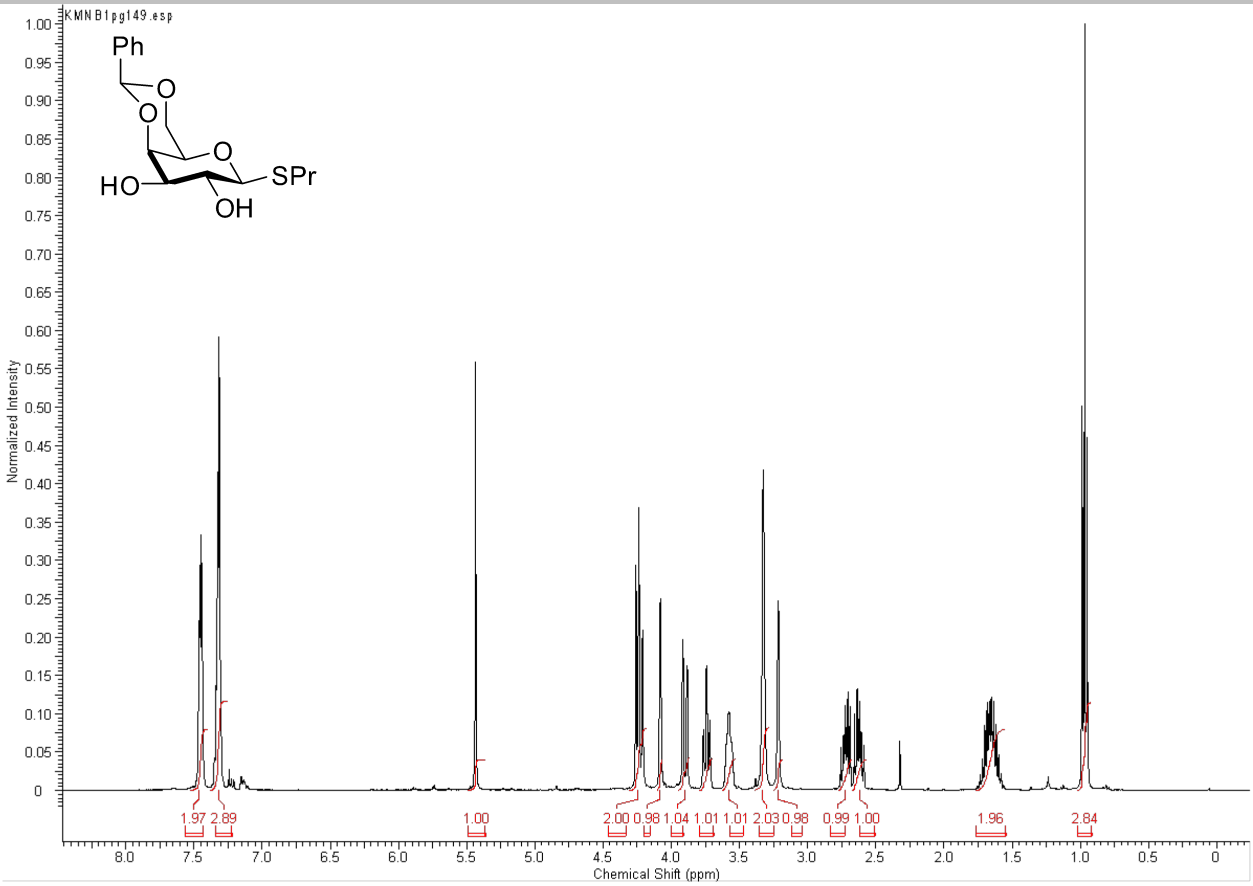

^{1}H NMR (400MHz ,CHLOROFORM-d) for compound **S3**

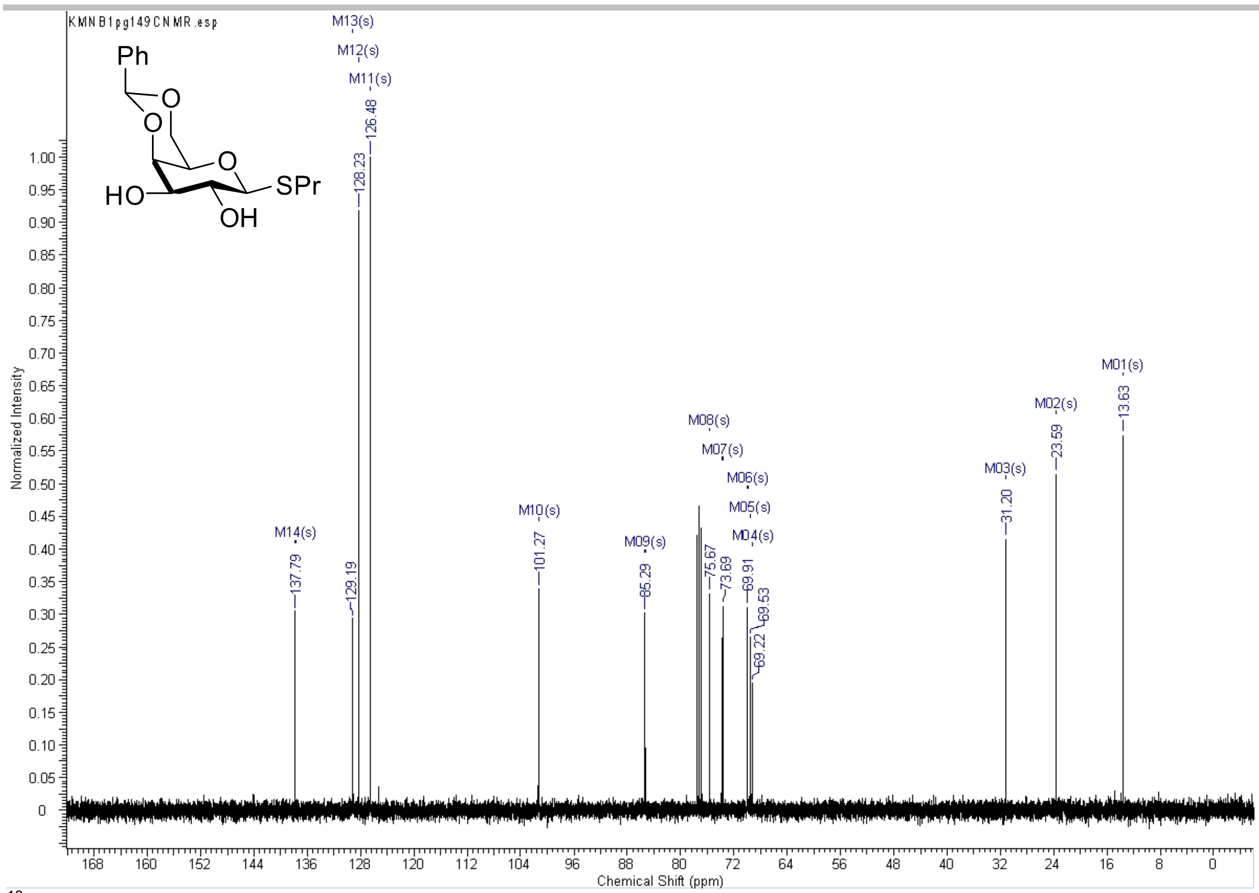

^{13}C NMR (101MHz ,CHLOROFORM-d) for compound **S3**

KMNB1pg149gCOSY.fid.esp

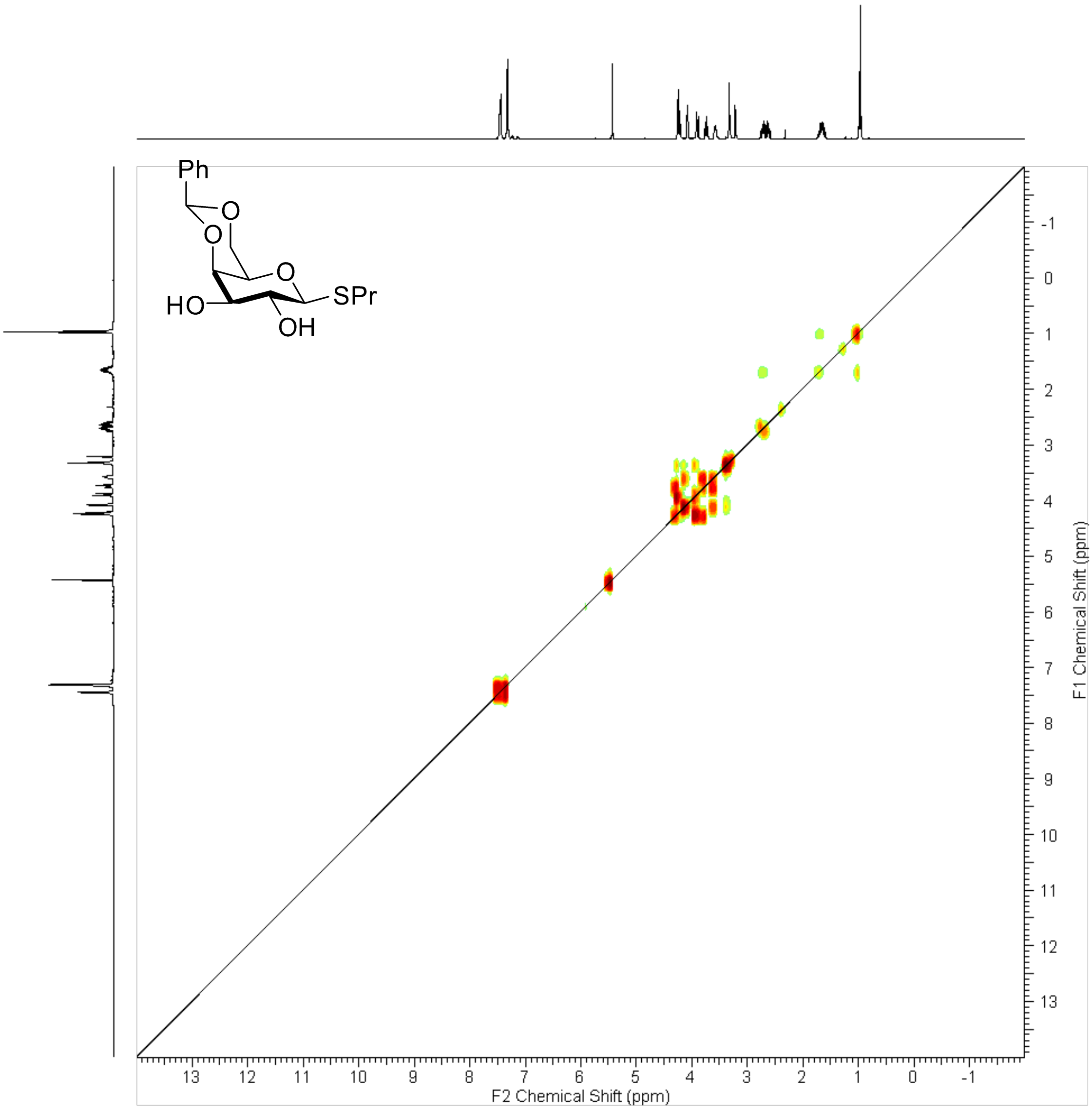

COSY (400MHz ,CHLOROFORM-d) for compound **S3**

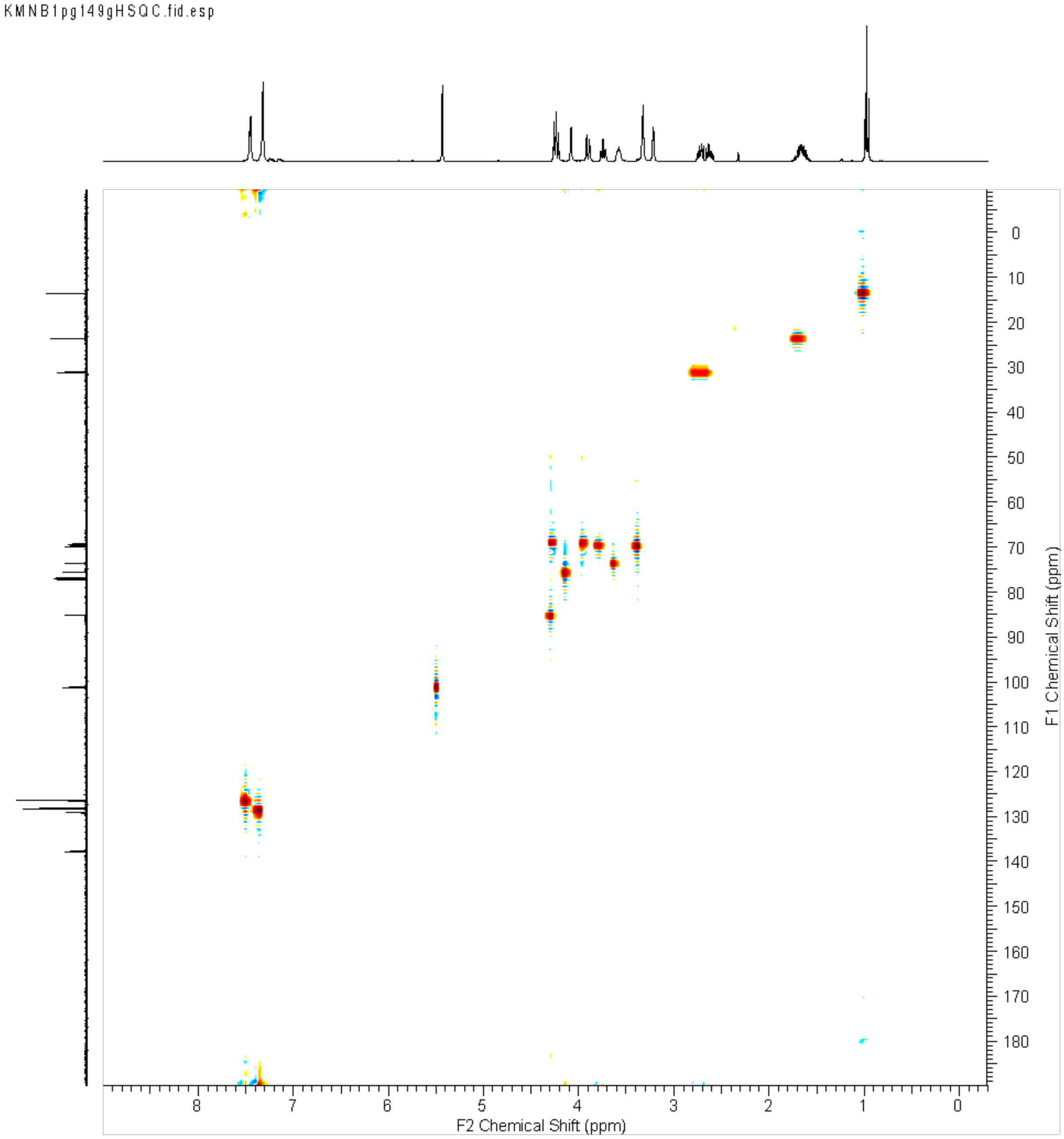

HSQC (400MHz ,CHLOROFORM-d) for compound **S3**

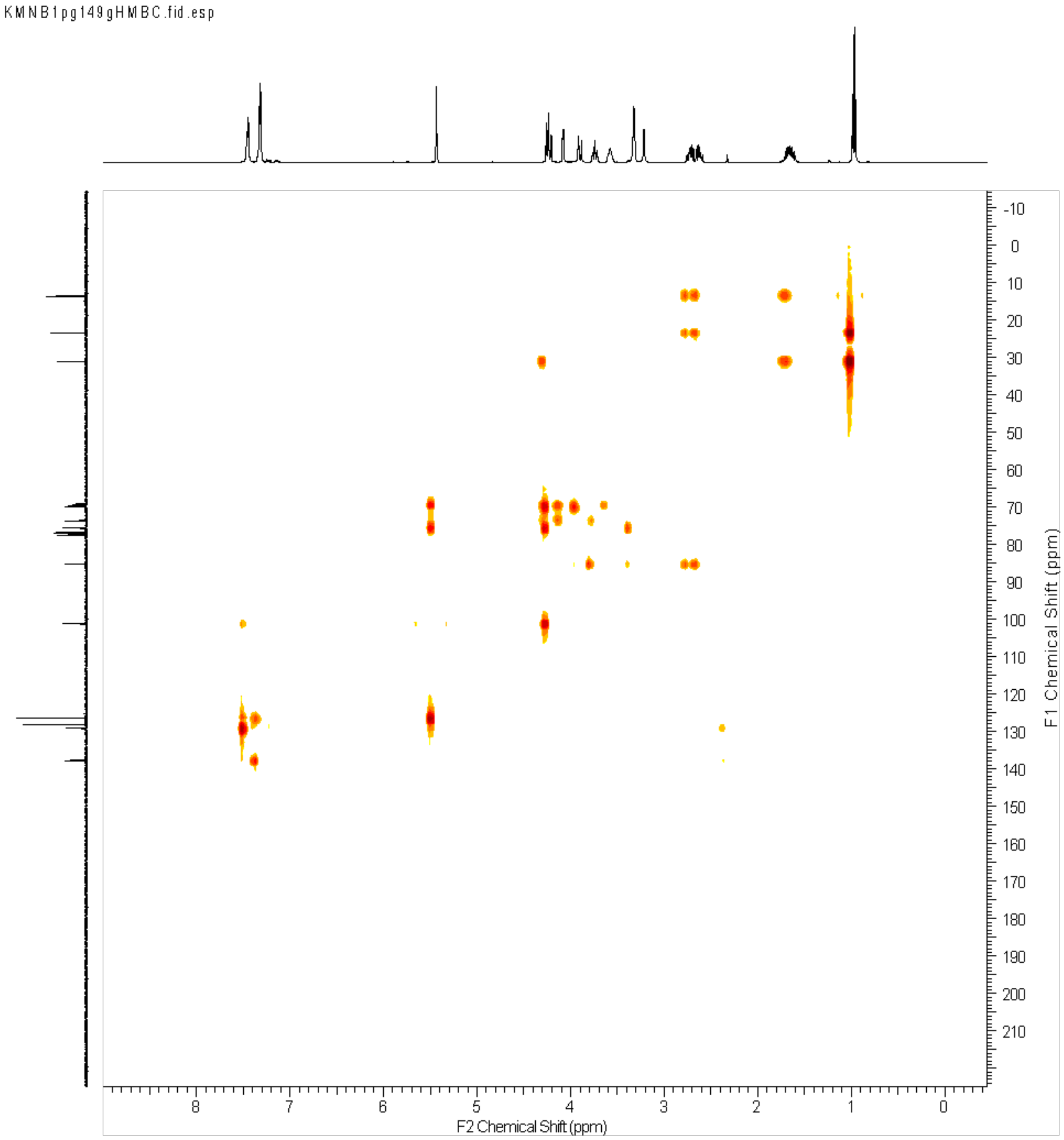

HMBC (400MHz ,CHLOROFORM-d) for compound **S3**

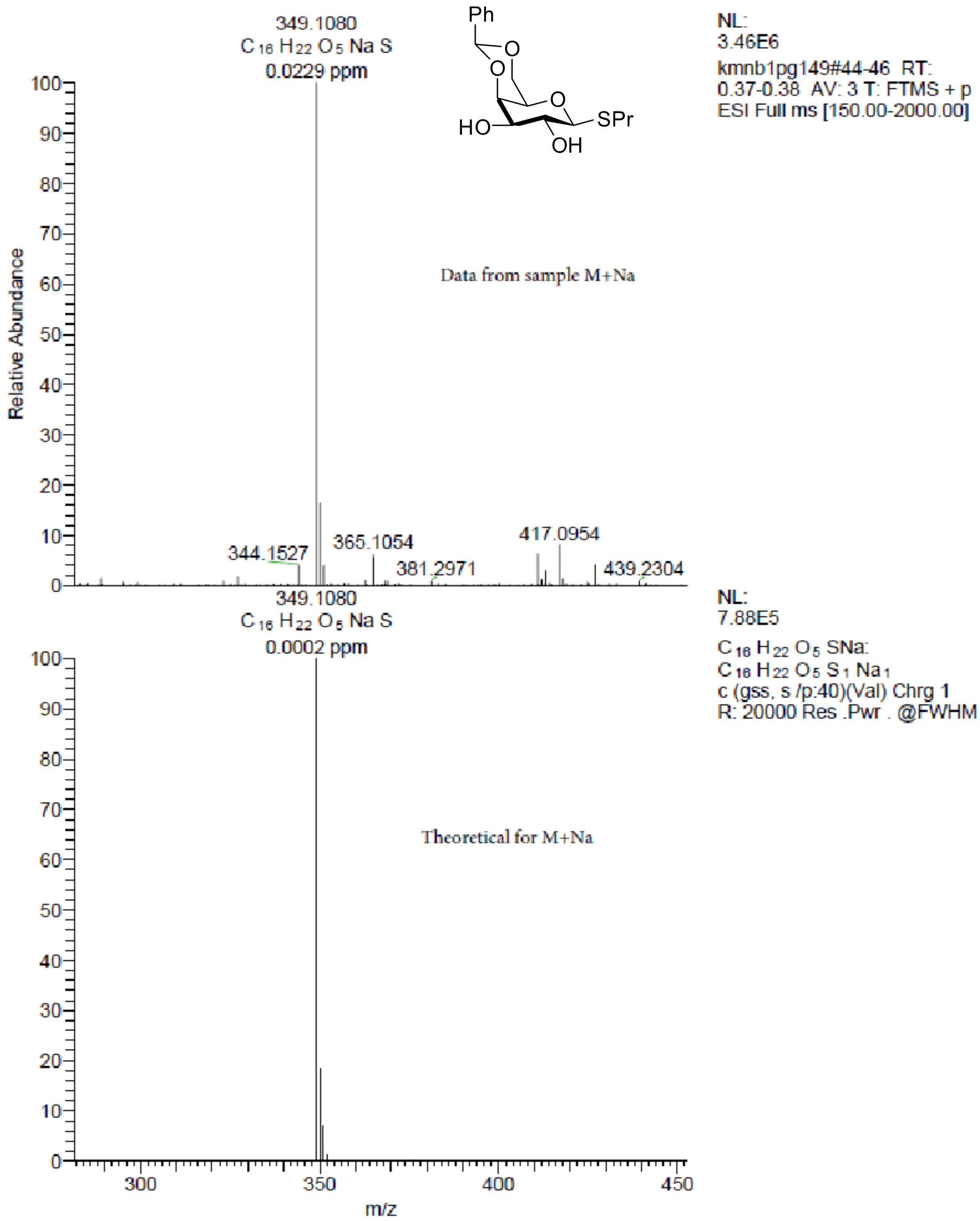

HRMS for compound **S3**

SUPPORTING INFORMATION

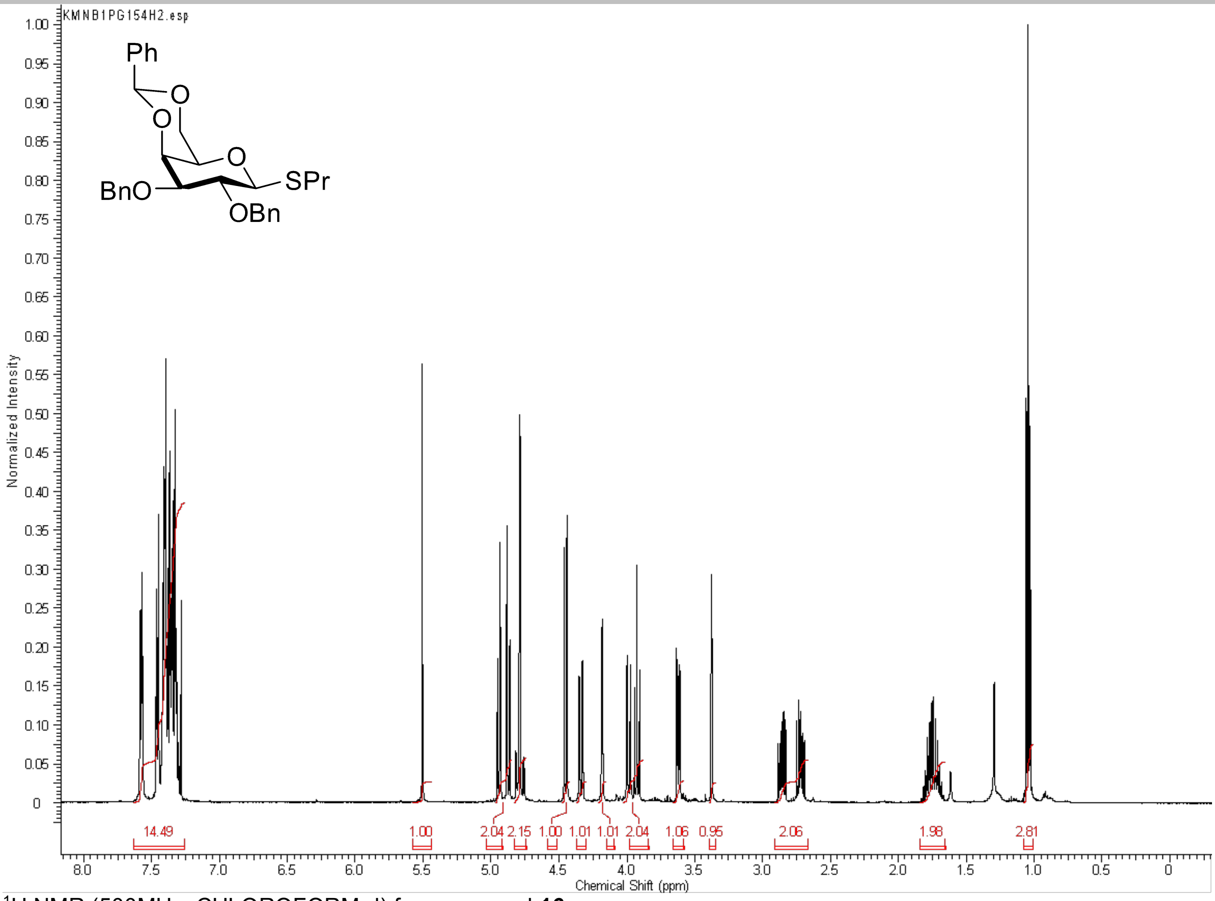

^{1}H NMR (500MHz ,CHLOROFORM-d) for compound **16**

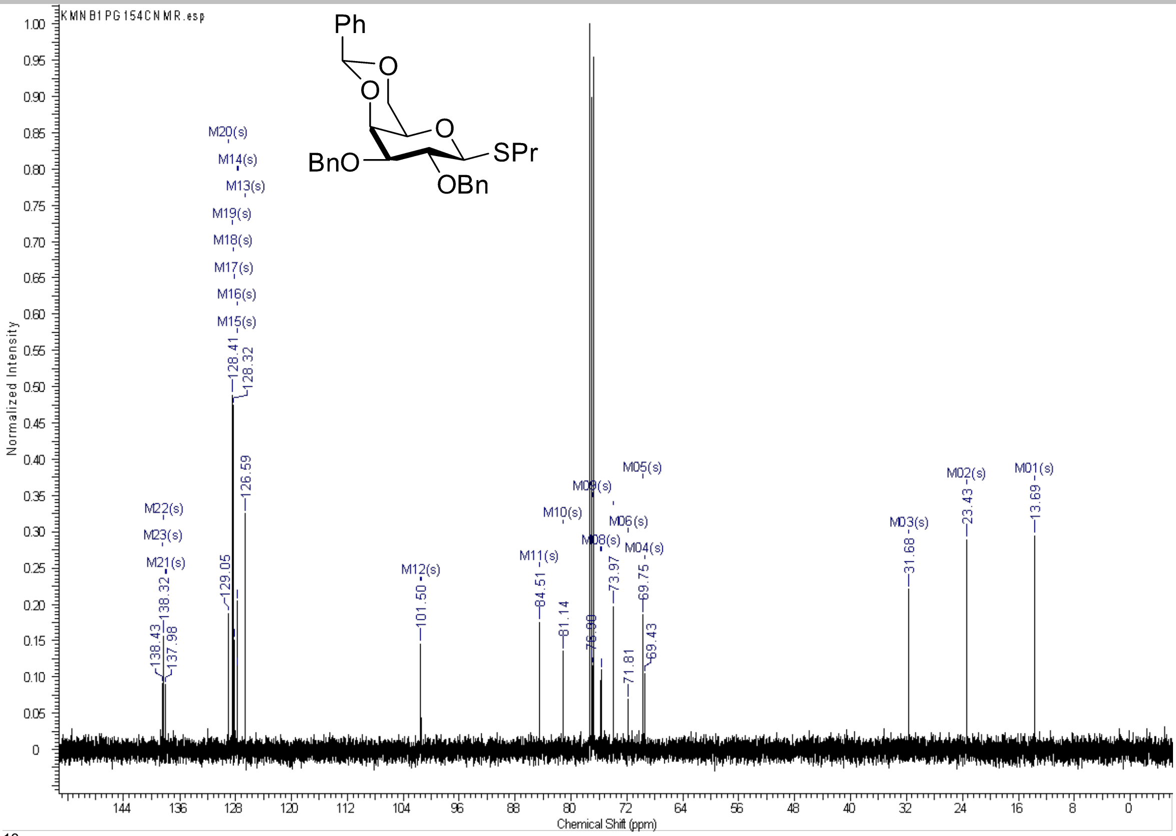

^{13}C NMR (126MHz ,CHLOROFORM-d) for compound **16**

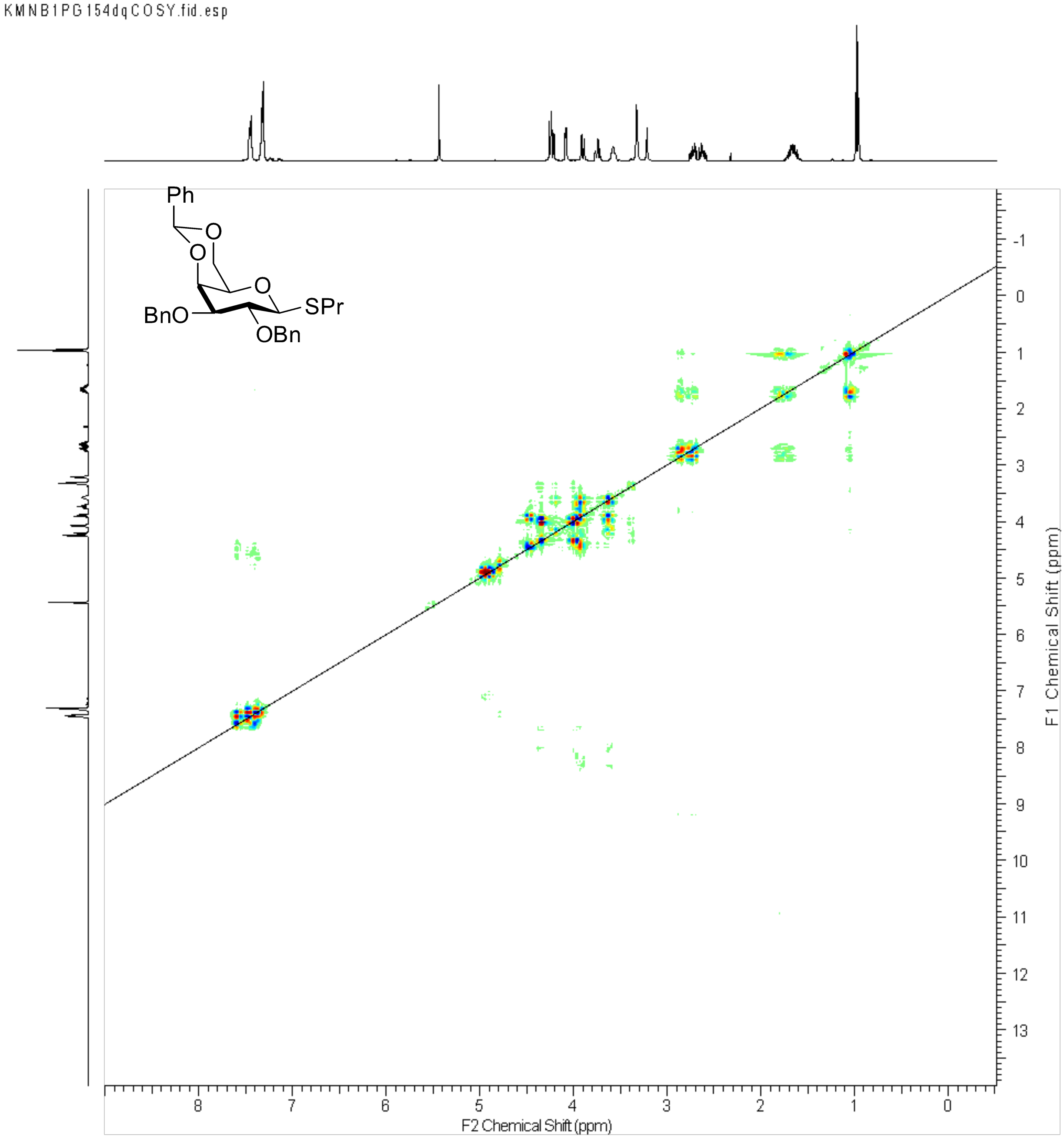

dqCOSY (500MHz ,CHLOROFORM-d) for compound **16**

SUPPORTING INFORMATION

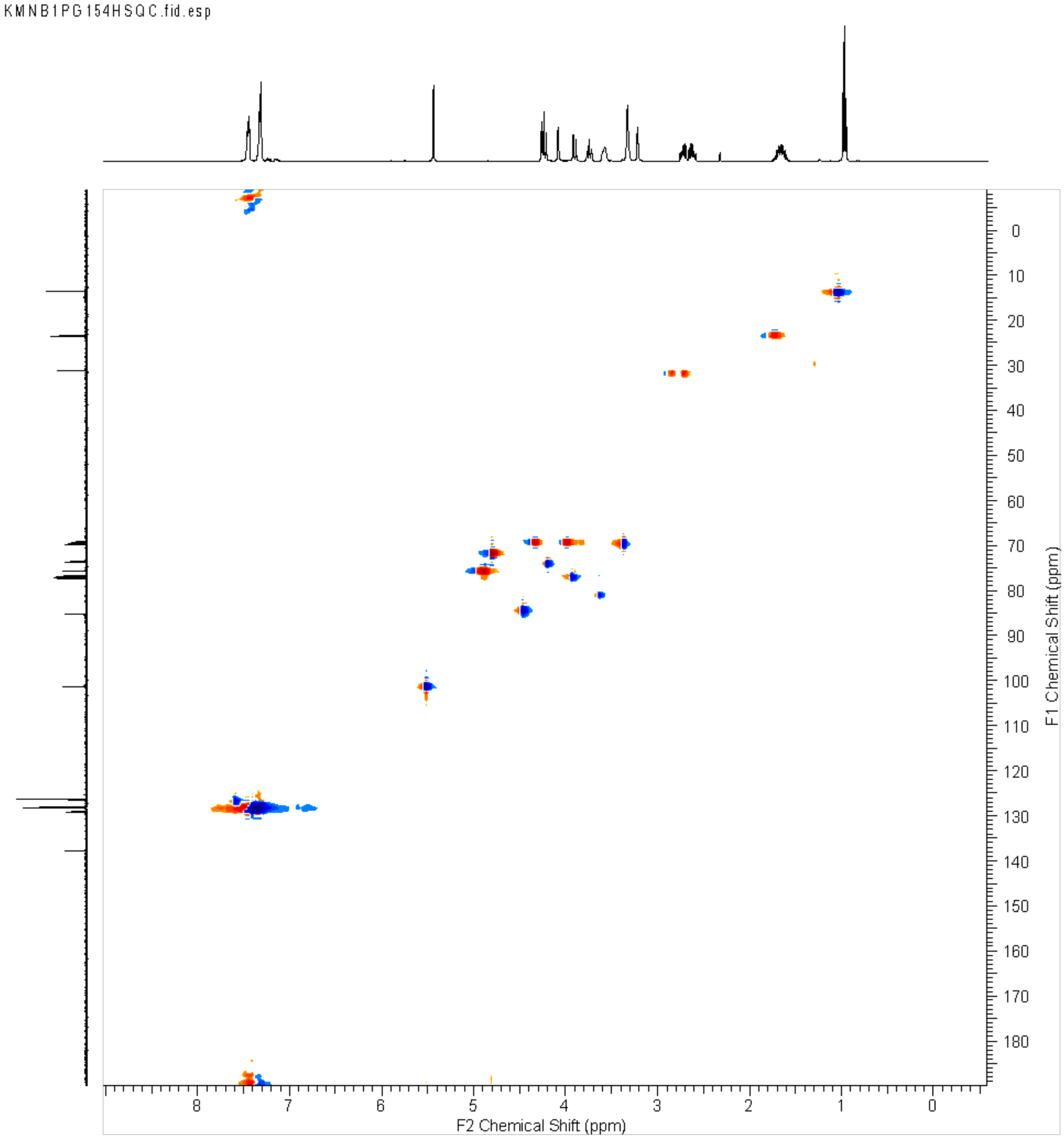

HSQC (500MHz ,CHLOROFORM-d) for compound **16**

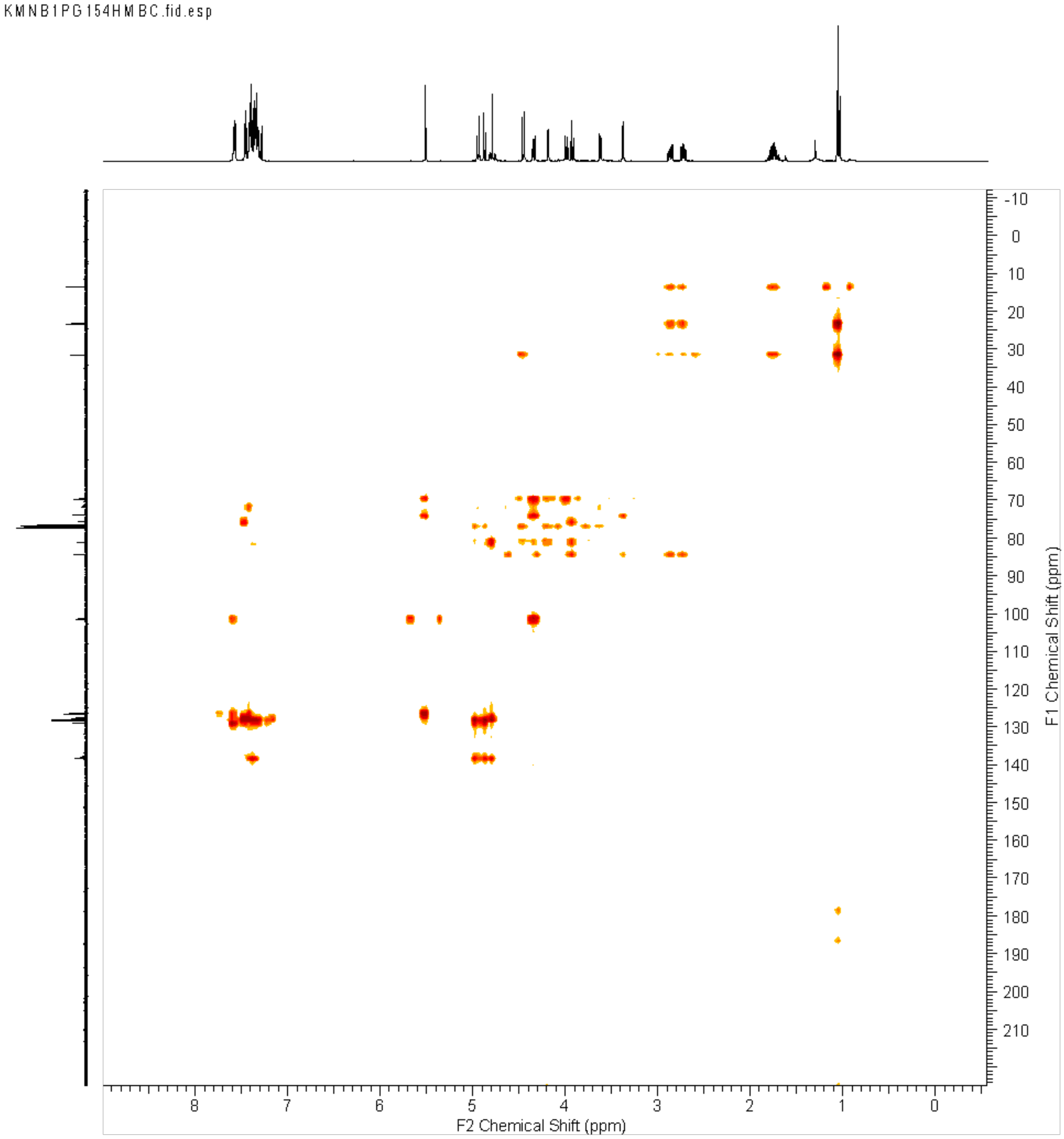

HMBC (500MHz ,CHLOROFORM-d) for compound **16**

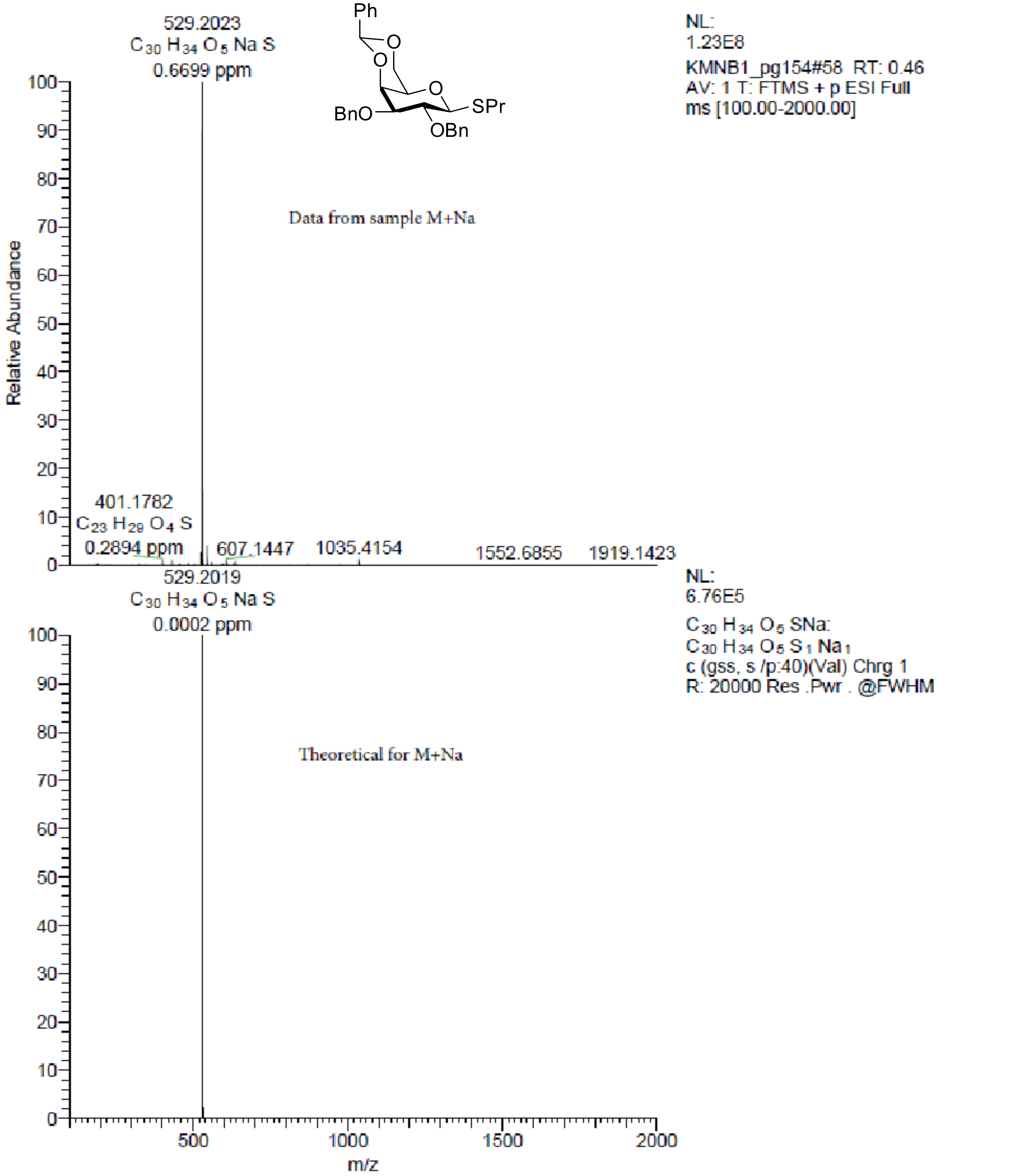

HRMS for compound **16**

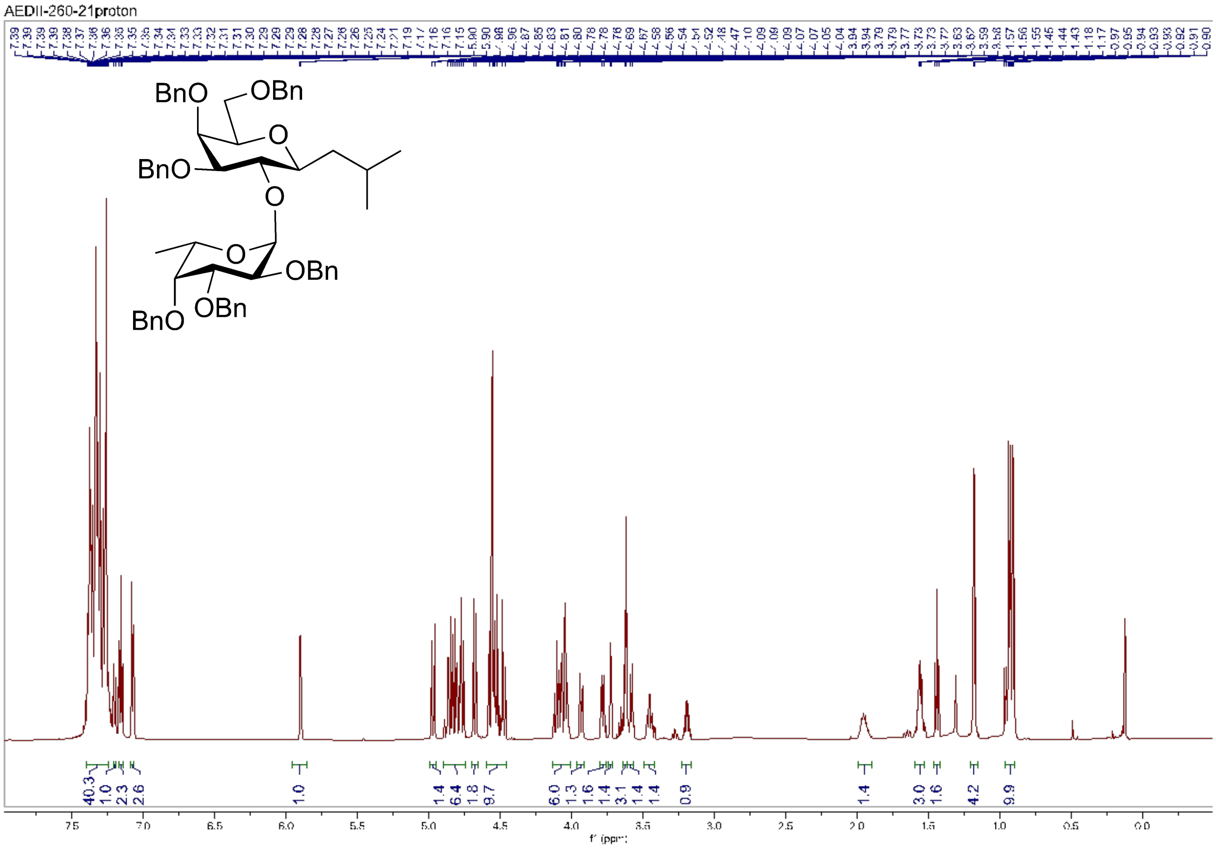

^{1}H NMR (600 MHz, $CDCl_3$) spectrum of compound **15a**

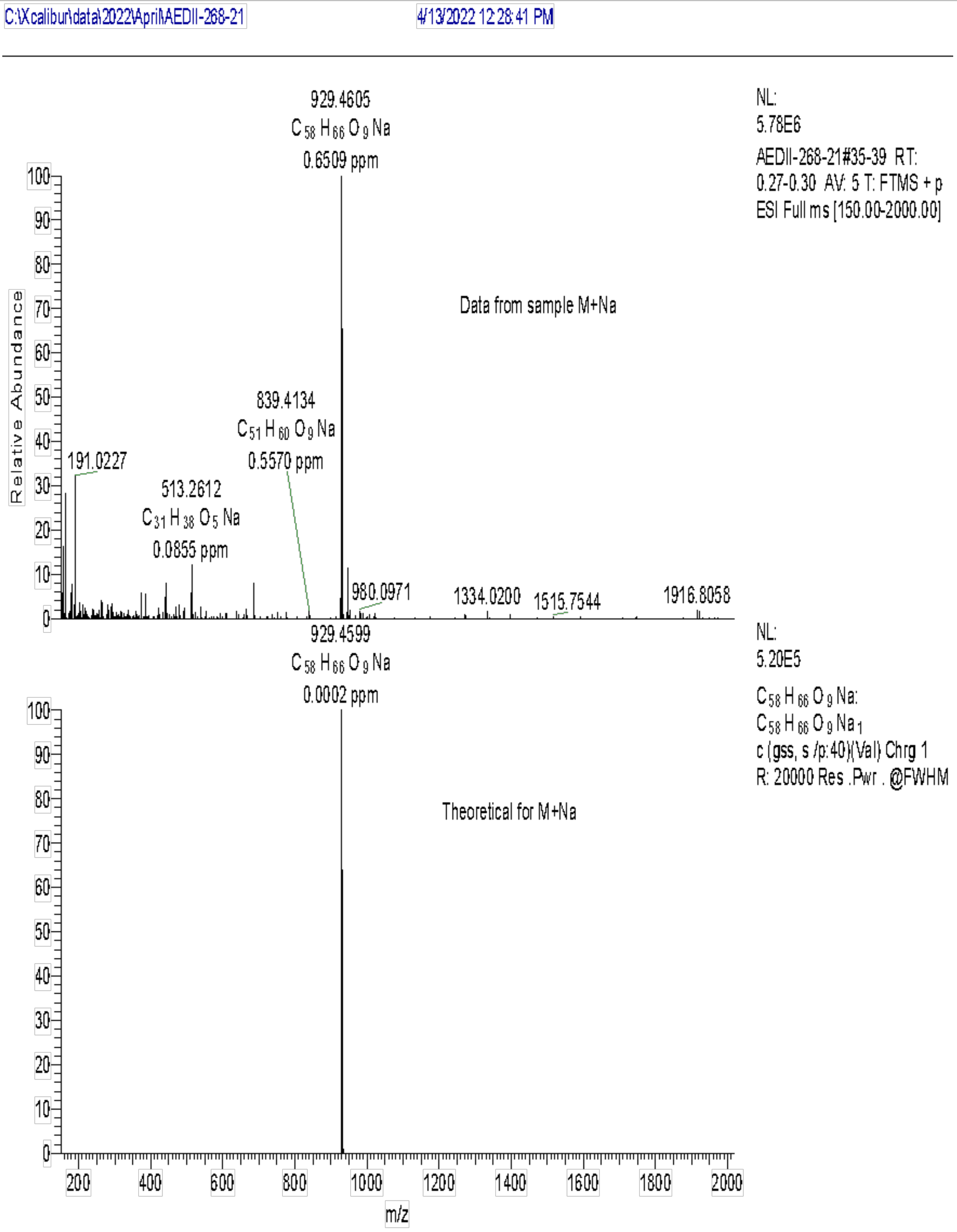

HRMS of compound **15a**

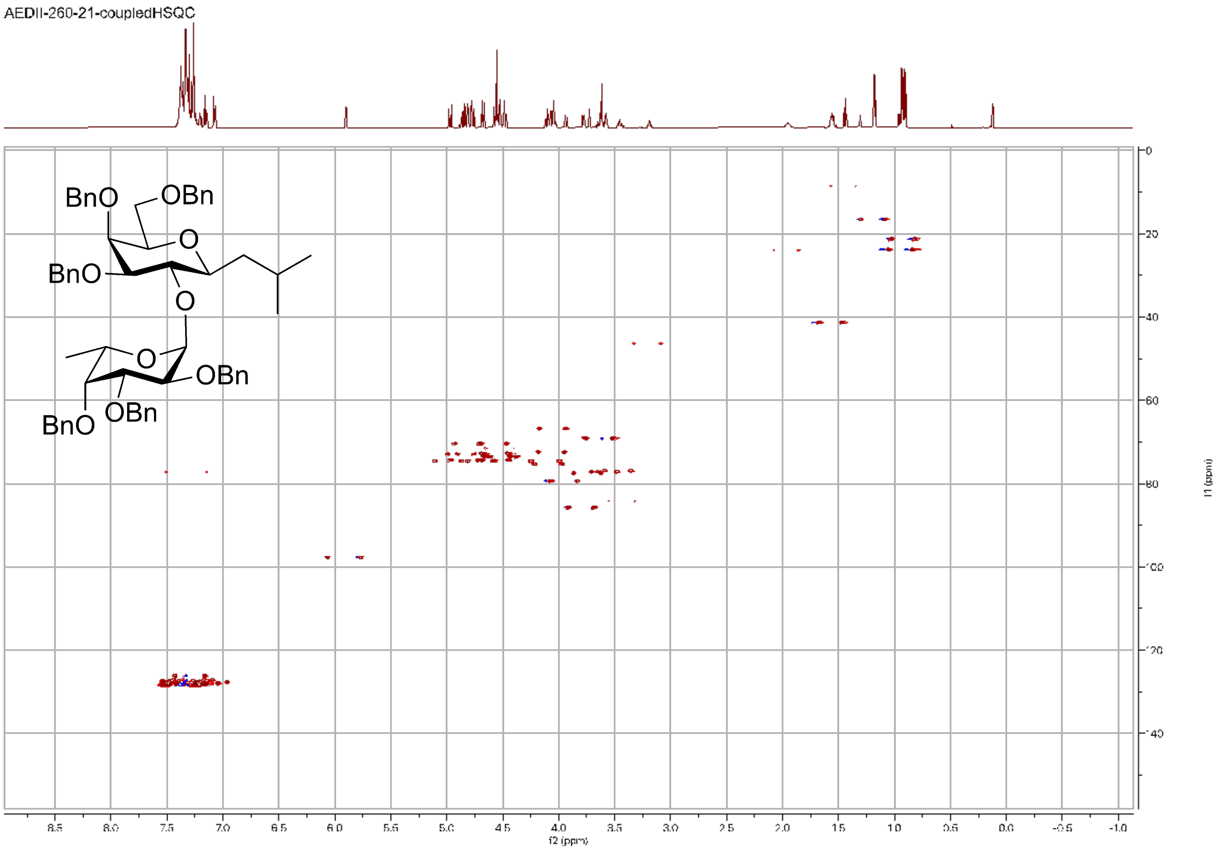

Coupled HSQC (600 MHz, $CDCl_3$) spectrum of compound **15a**

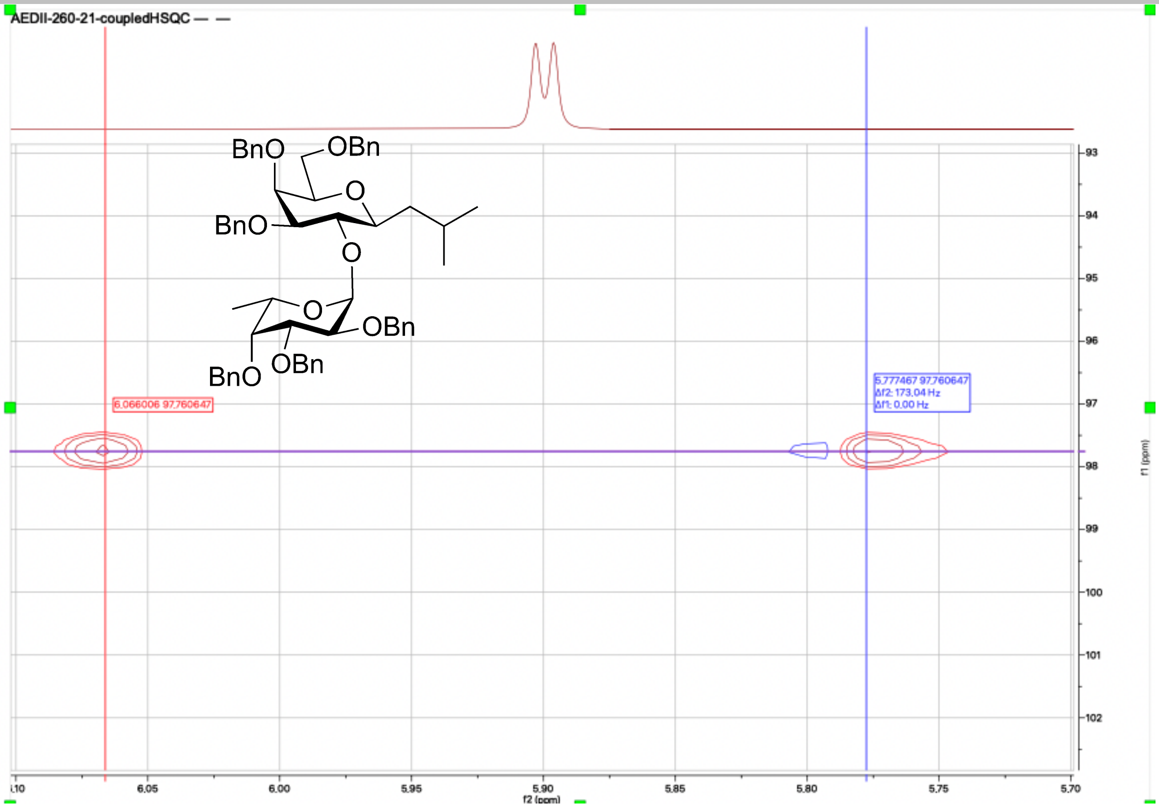

Zoom in on anomeric C-H of coupled HSQC. J value of 173 Hz indicates equatorial anomeric proton.

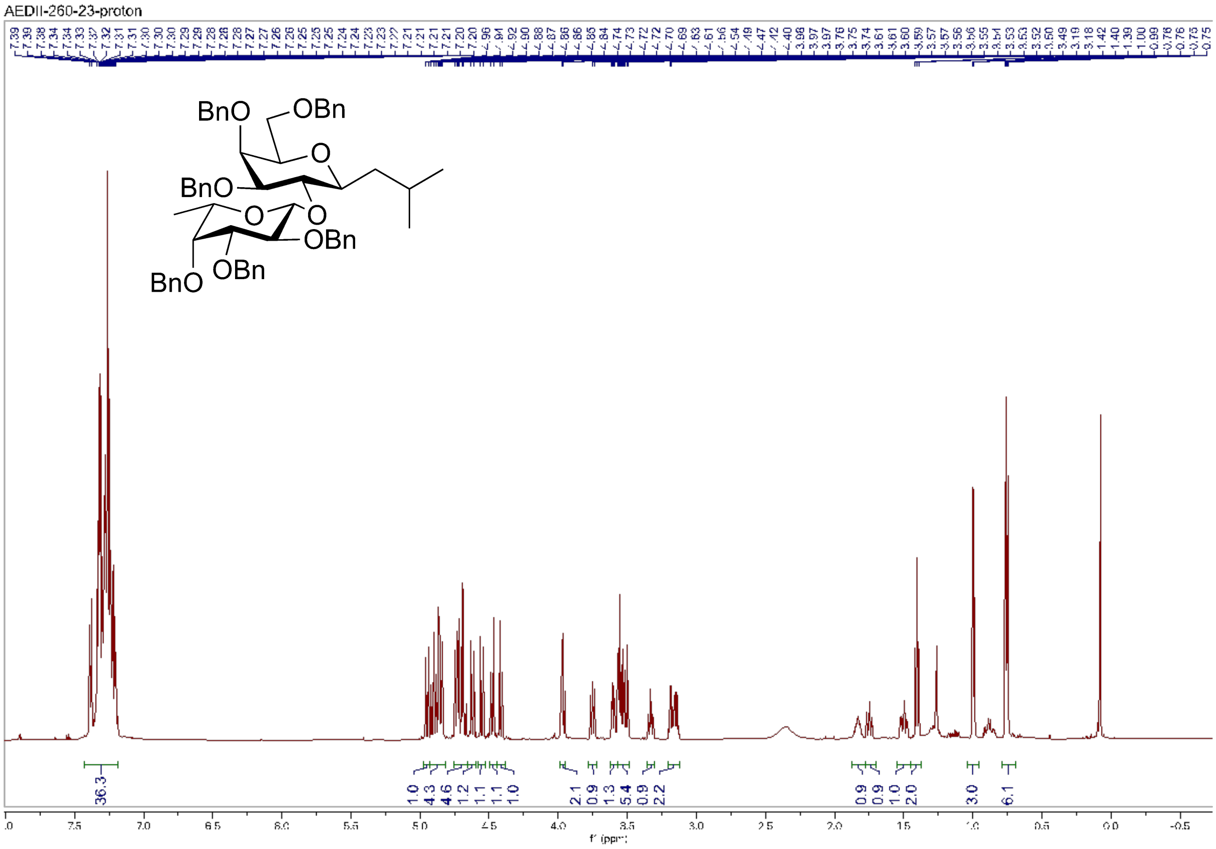

^{1}H NMR (600 MHz, $CDCl_3$) spectrum of **15b**

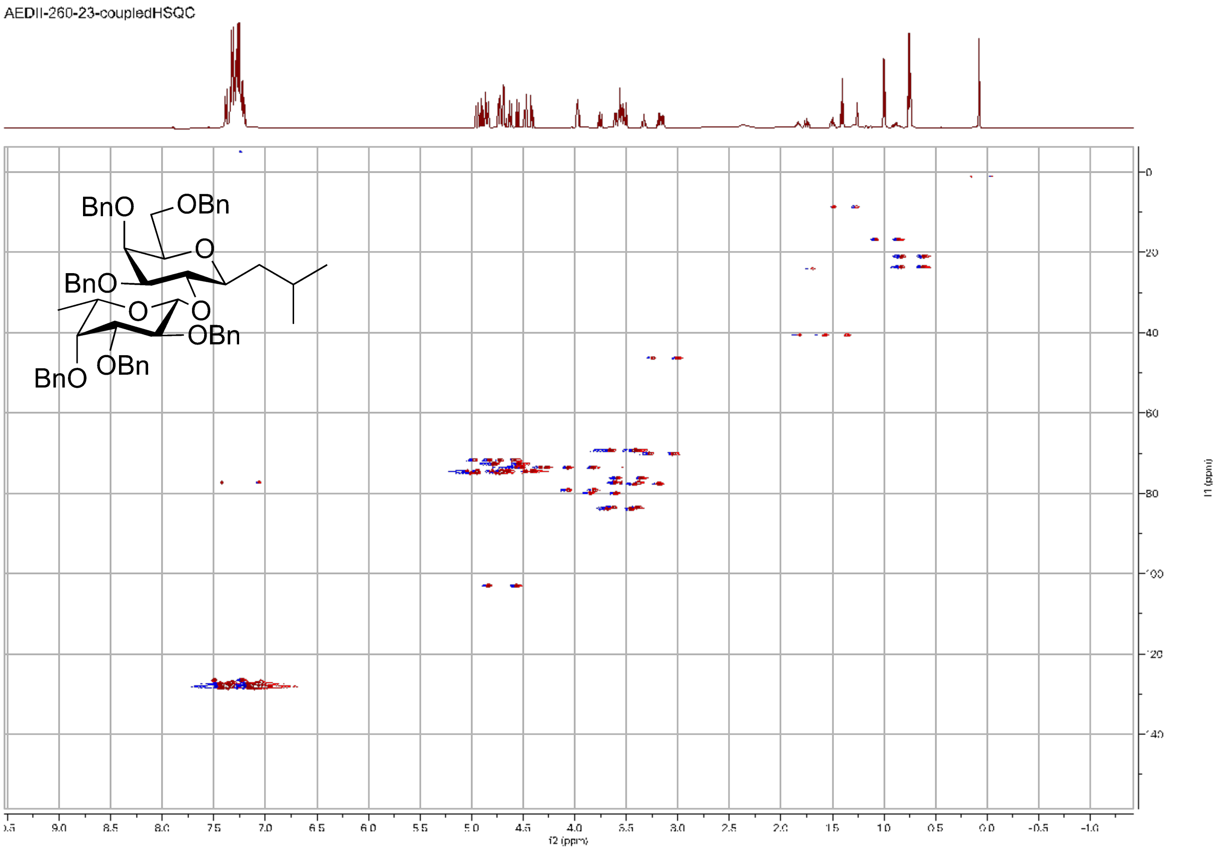

Coupled HSQC (600 MHz, $CDCl_3$) spectrum of **15b**

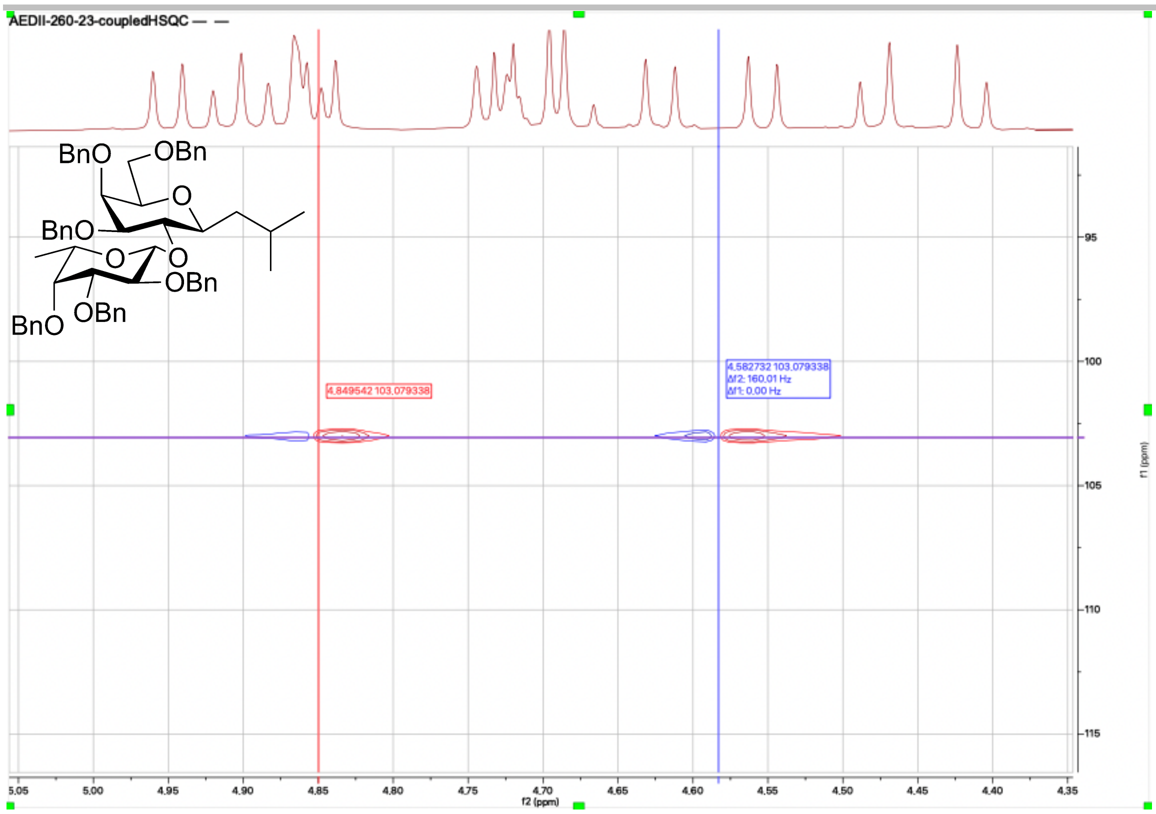

Zoom in of coupled HSQC on anomeric C-H. J value of 160 Hz indicates axial proton.

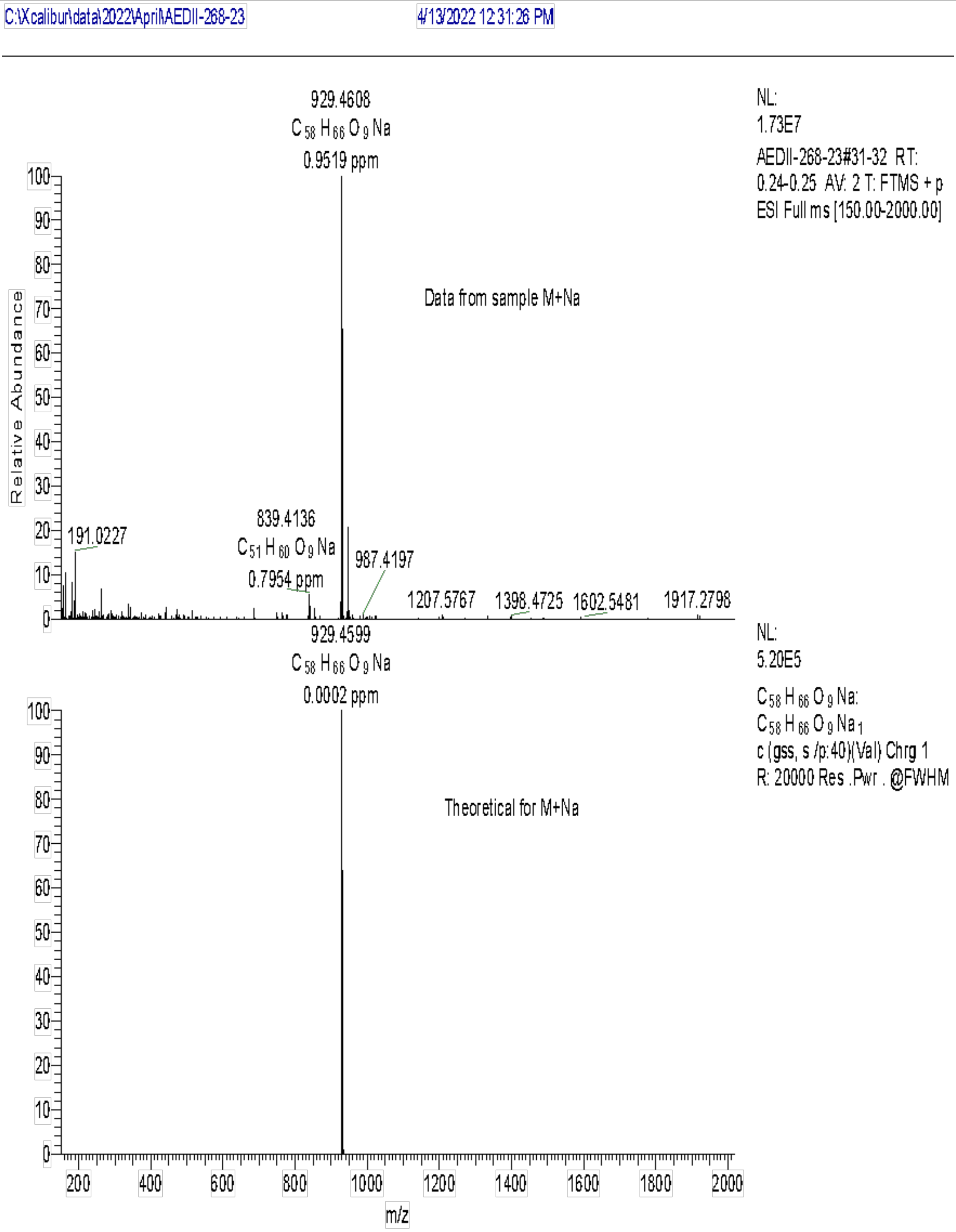

HRMS of compound **15b**

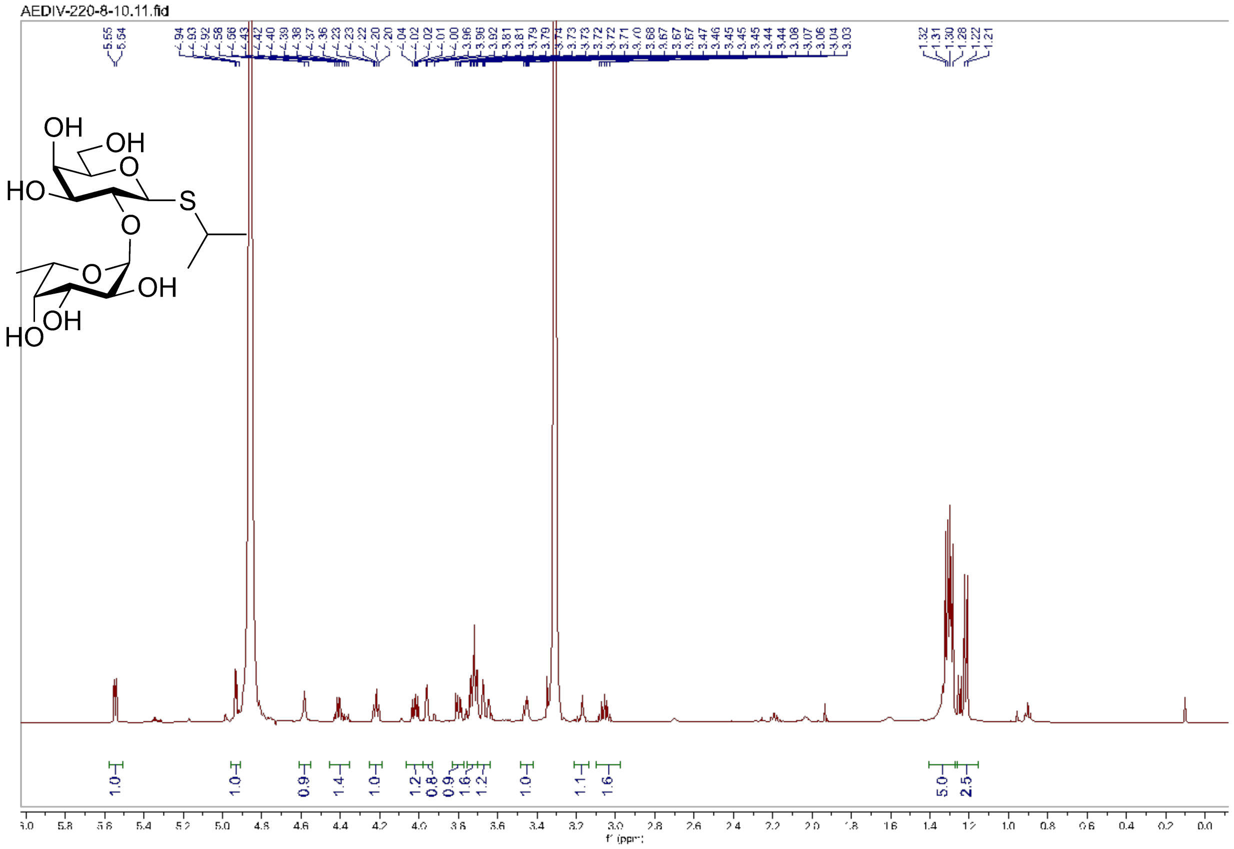

^{1}H NMR (500 MHz, CD_3OD) spectrum of compound **2a**

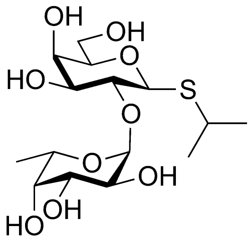

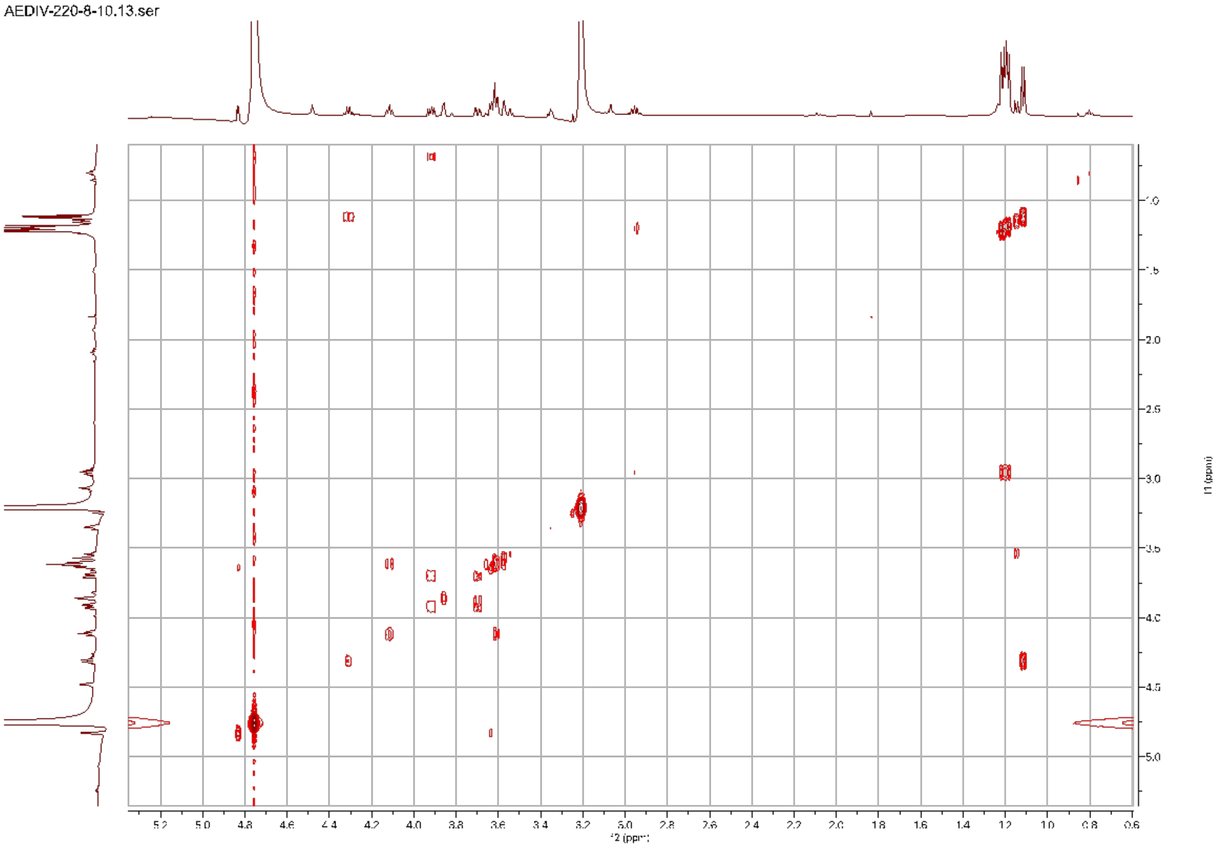

COSY (500 MHz, CD_3OD) spectrum of compound **2a**

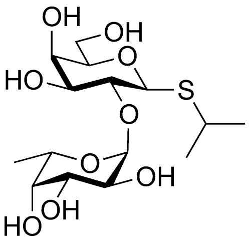

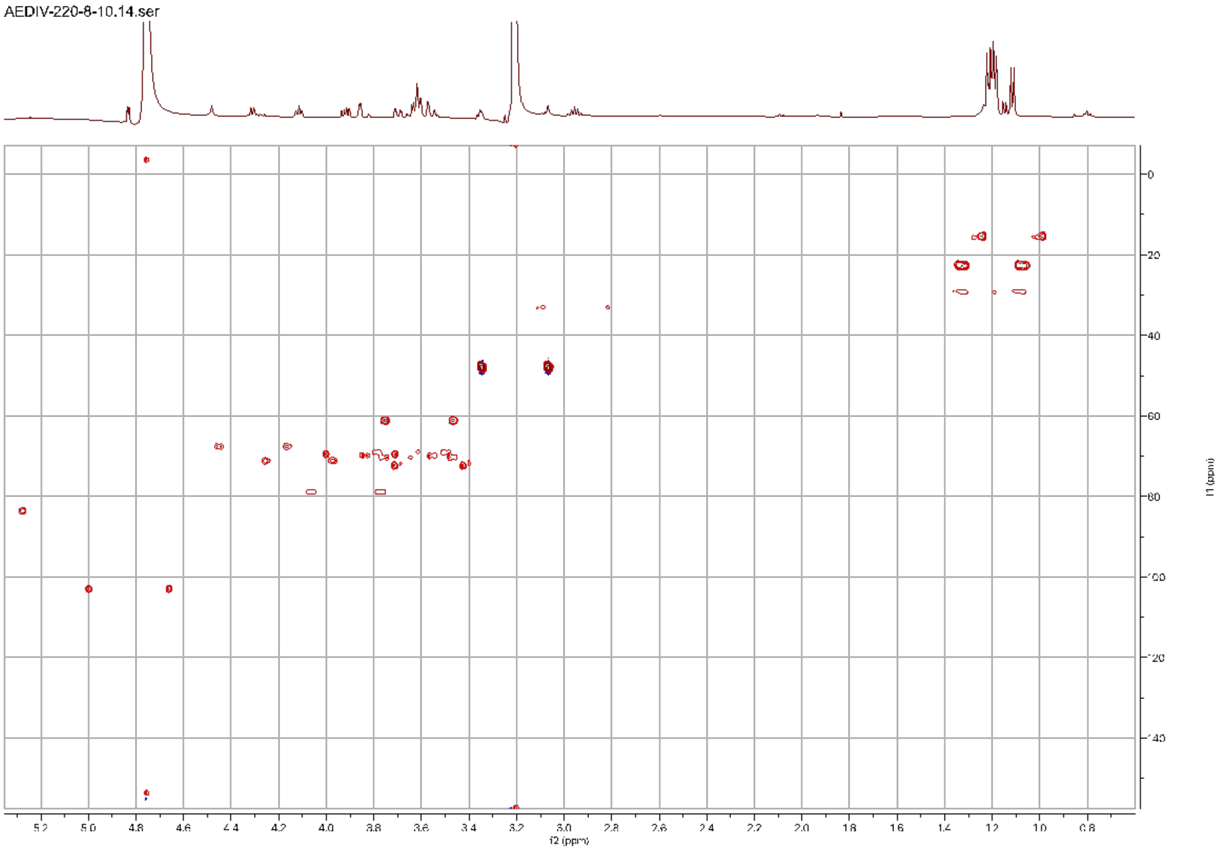

Coupled HSQC (500 MHz, CD_3OD) of compound **2a**

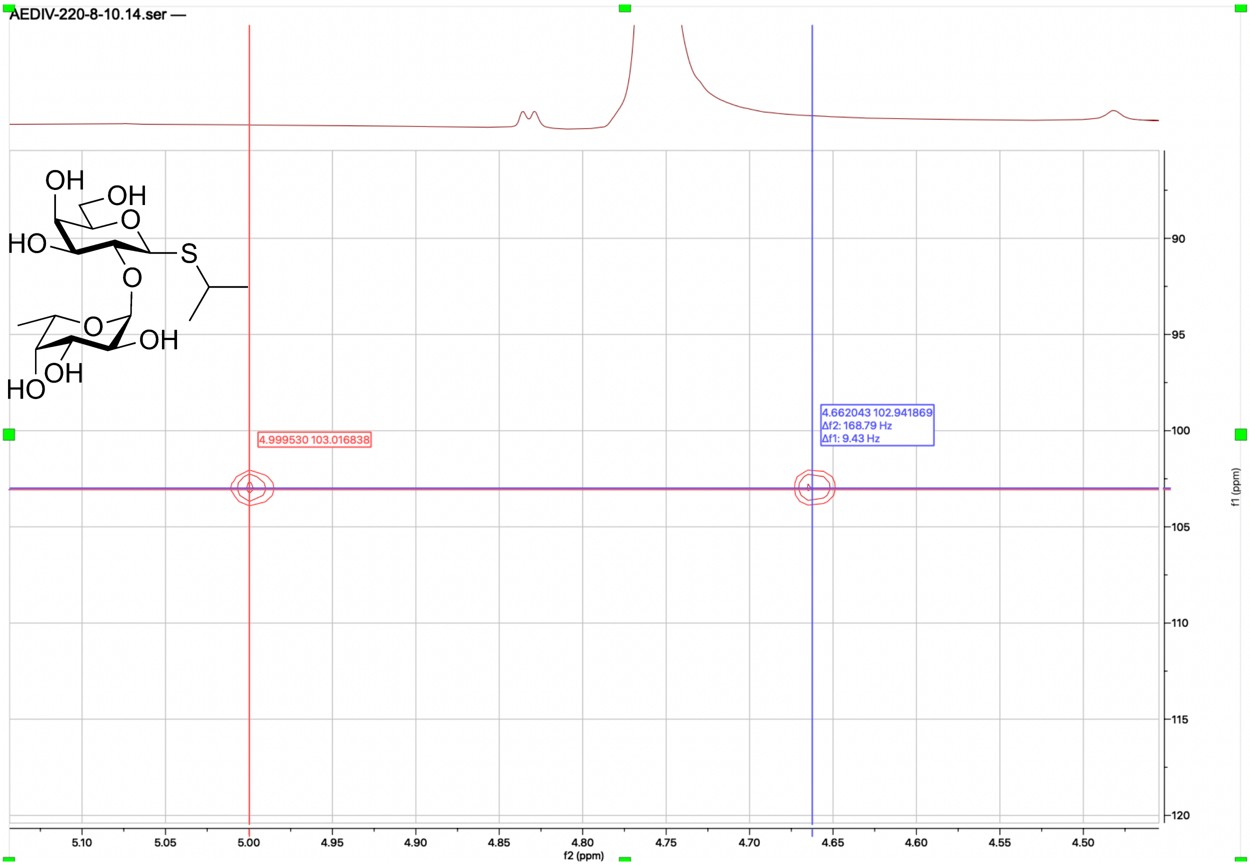

Zoom in of coupled HSQC on anomeric C-H. J value of 168 Hz indicates equatorial proton.

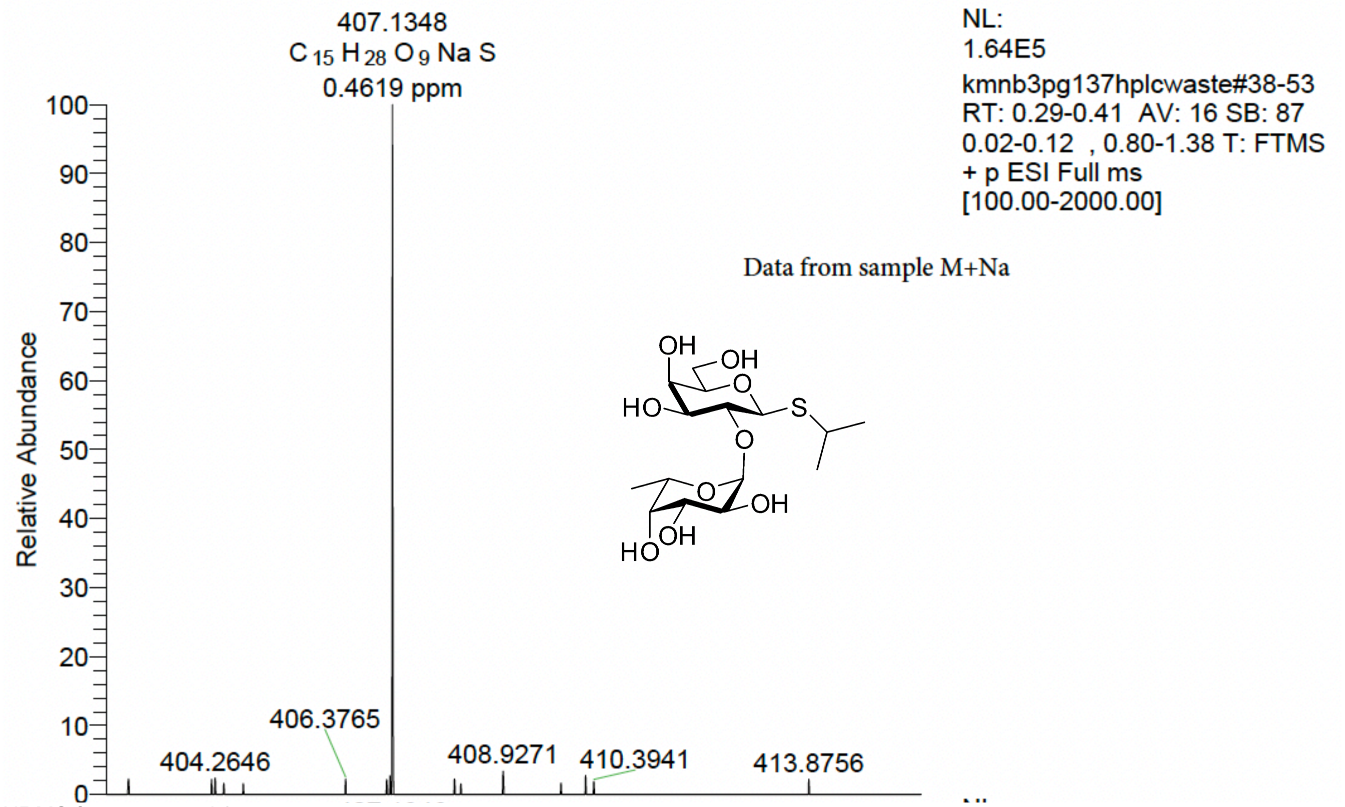

HRMS for compound **2a**

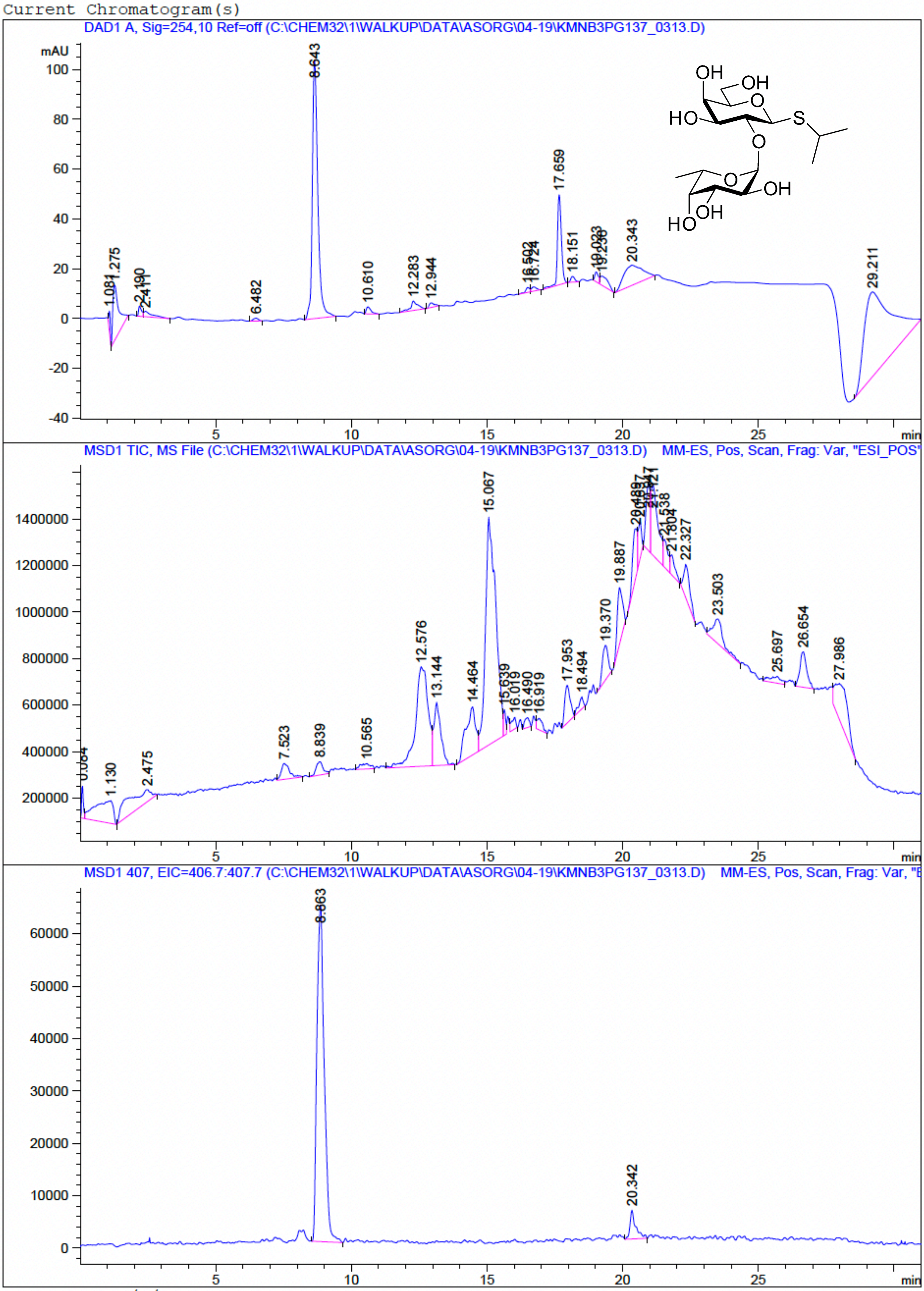

LCMS for compound **2a**

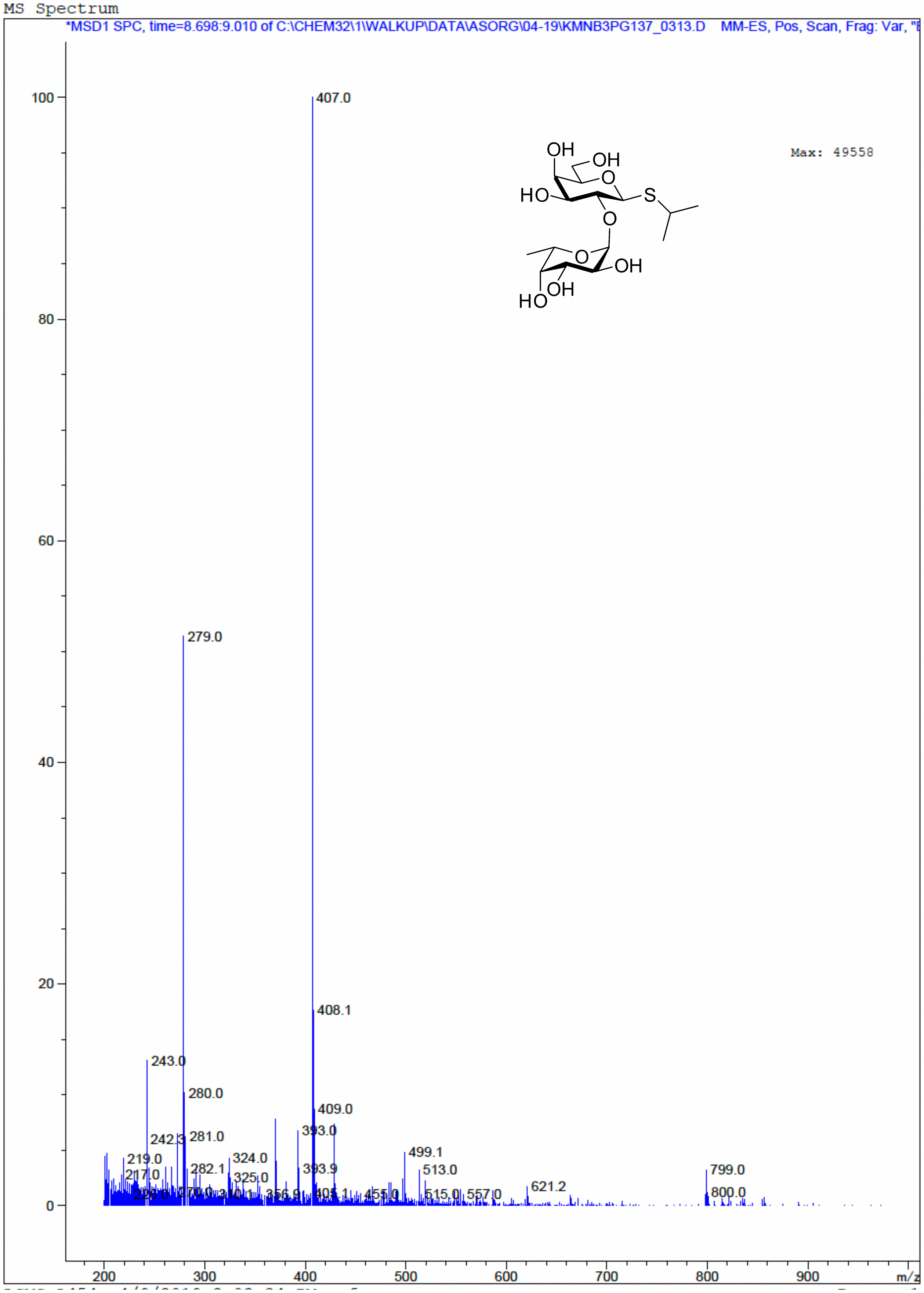

LCMS continued for compound **2a**

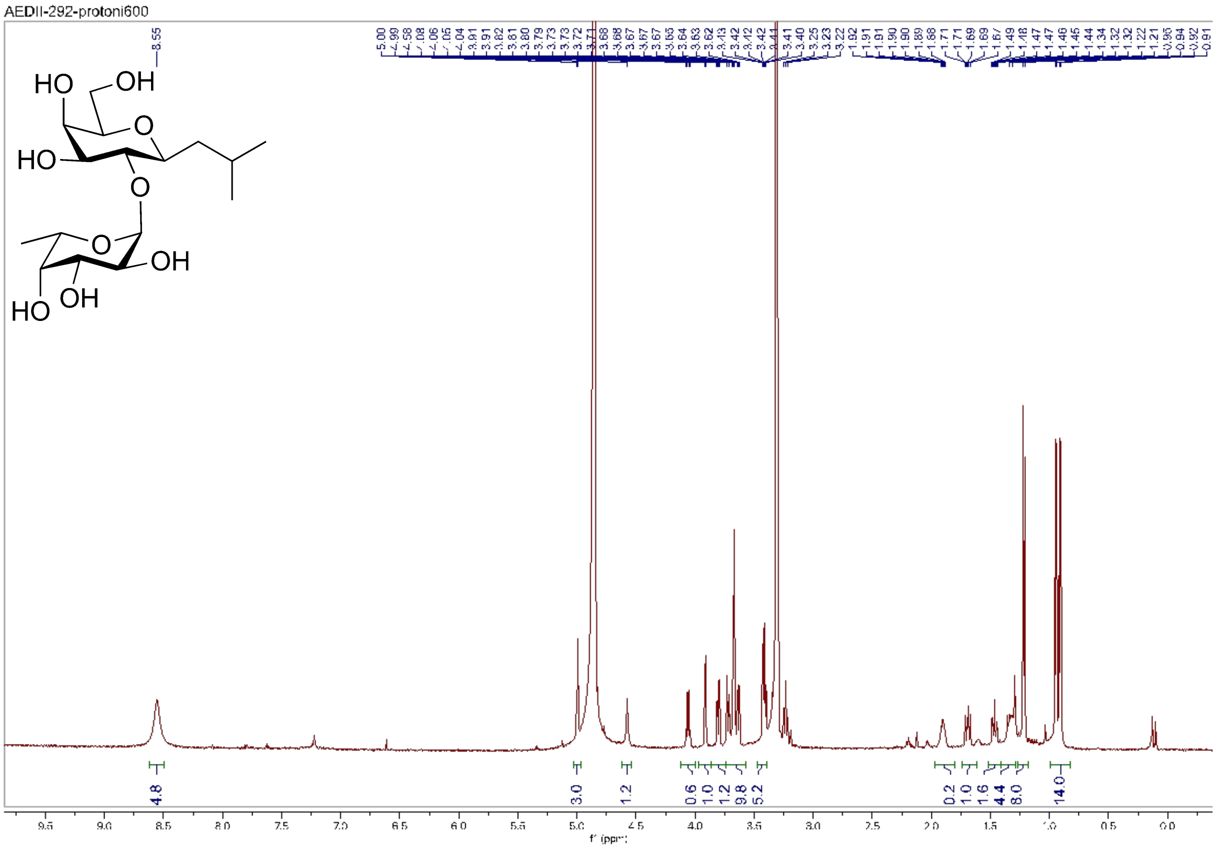

^{1}H NMR (600 MHz, CD_3OD) spectrum of compound **2b**

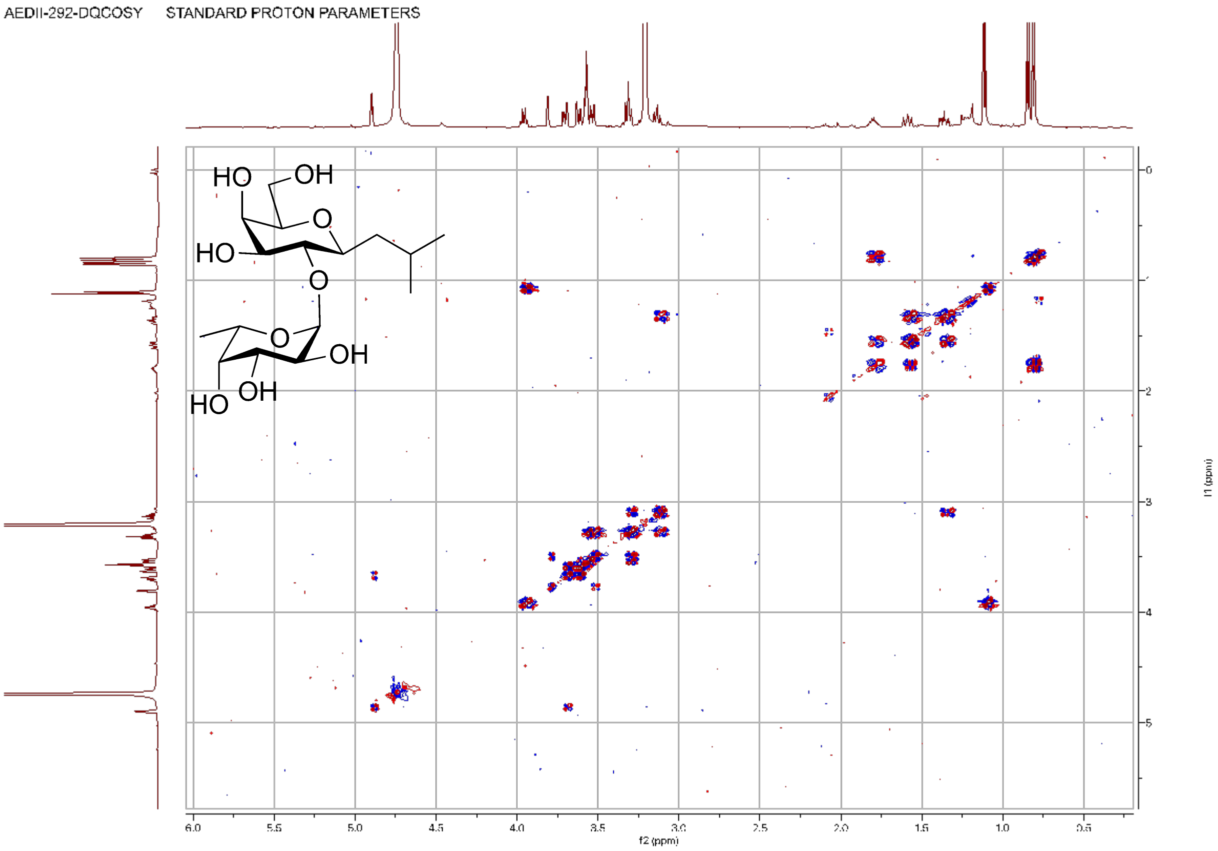

COSY (600 MHz, CD_3OD) spectrum of compound **2b**

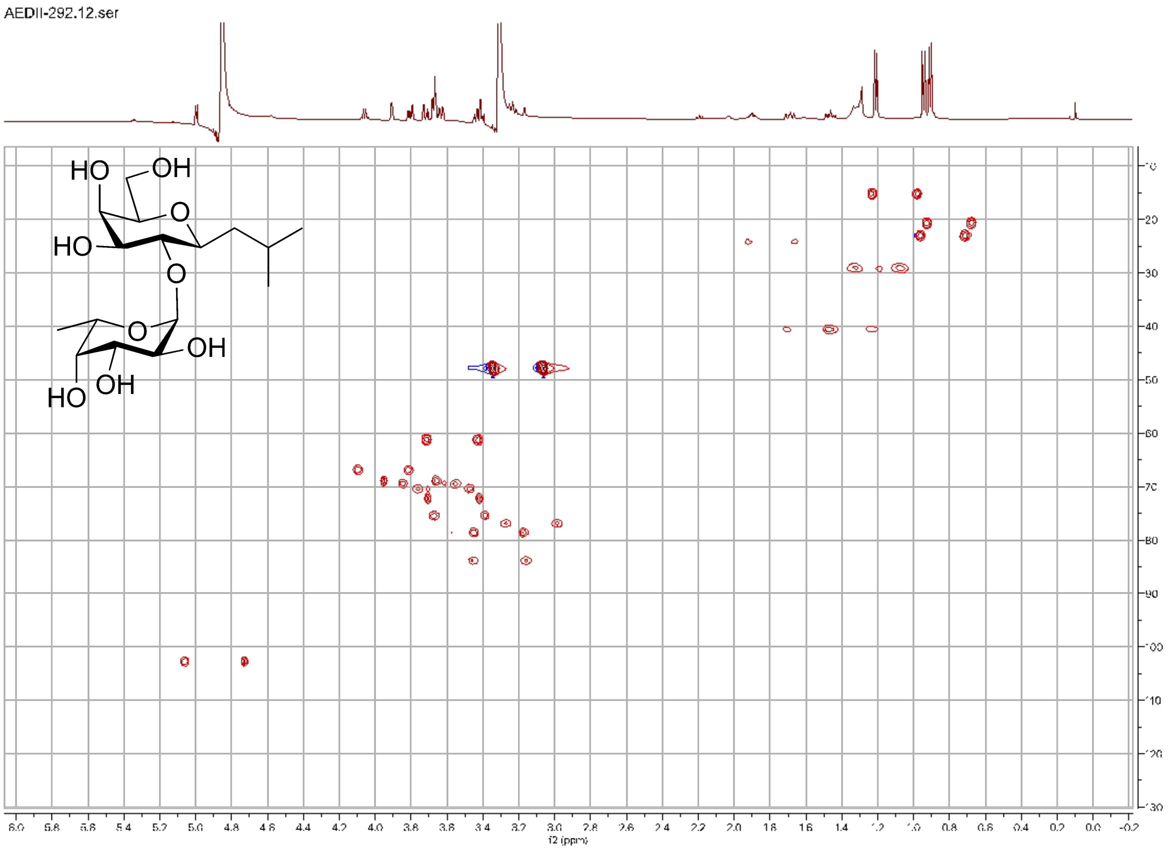

Coupled HSQC (500 MHz, CD_3OD) of compound **2b**

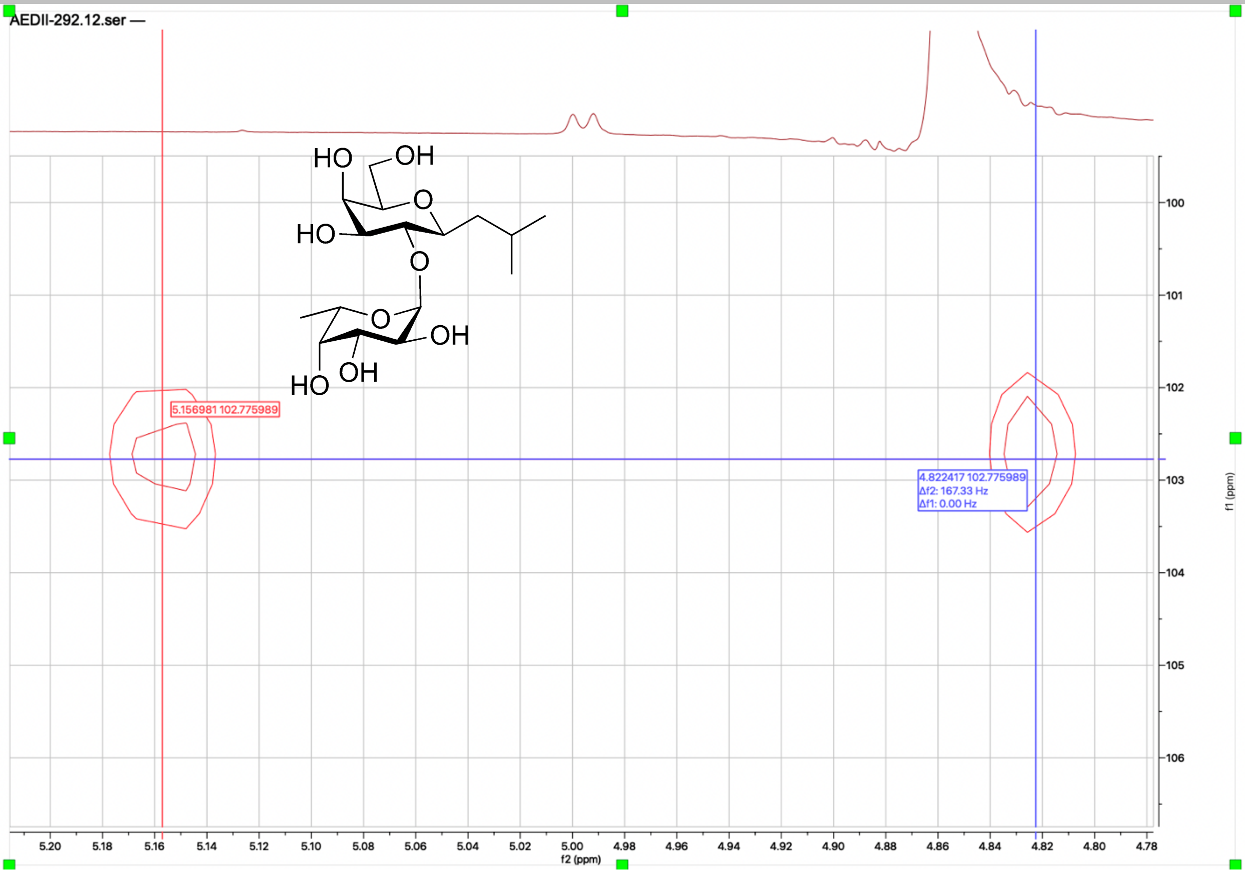

Zoom in of coupled HSQC on anomeric C-H. J value of 167 Hz indicates equatorial proton.

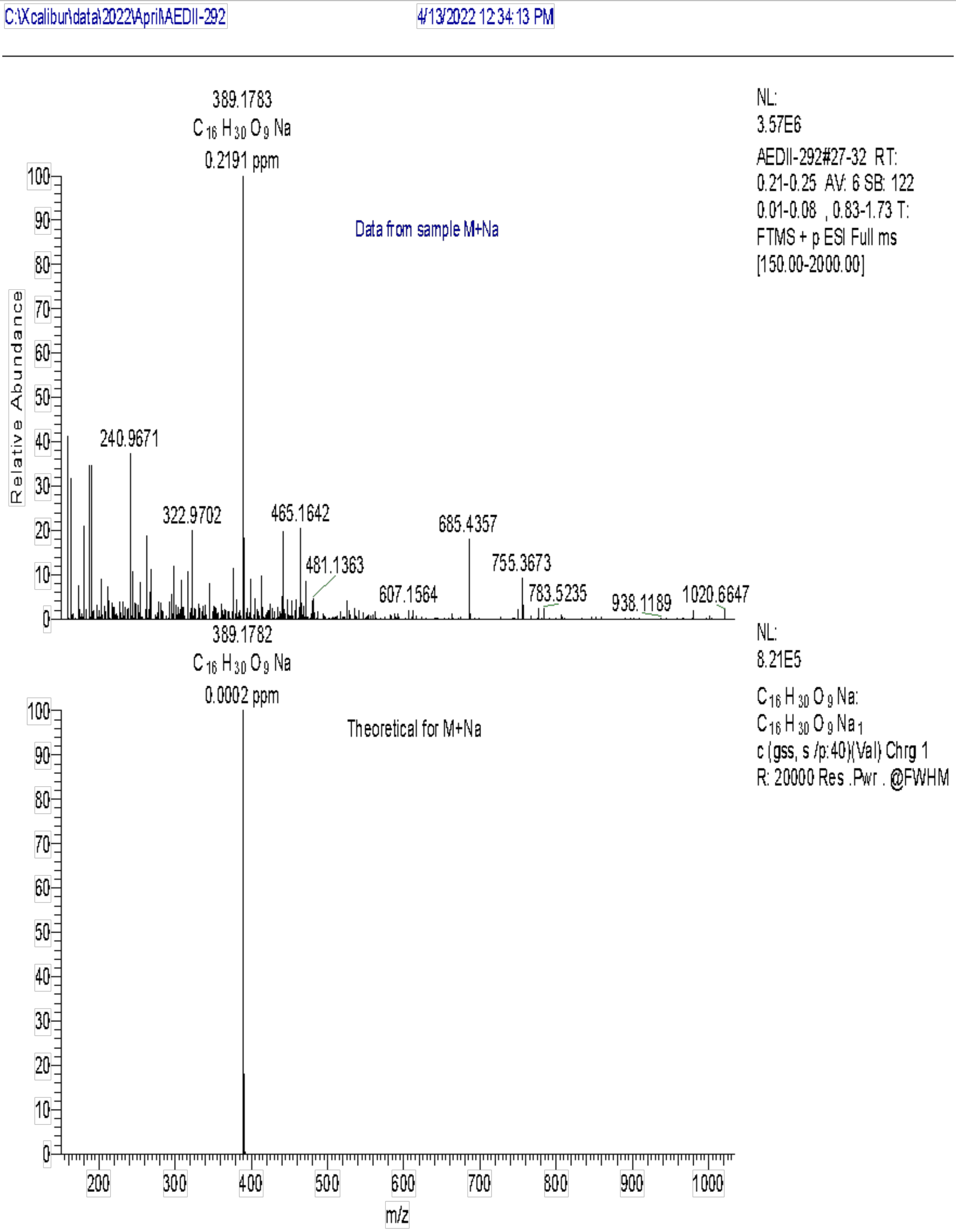

HRMS of compound **2b**

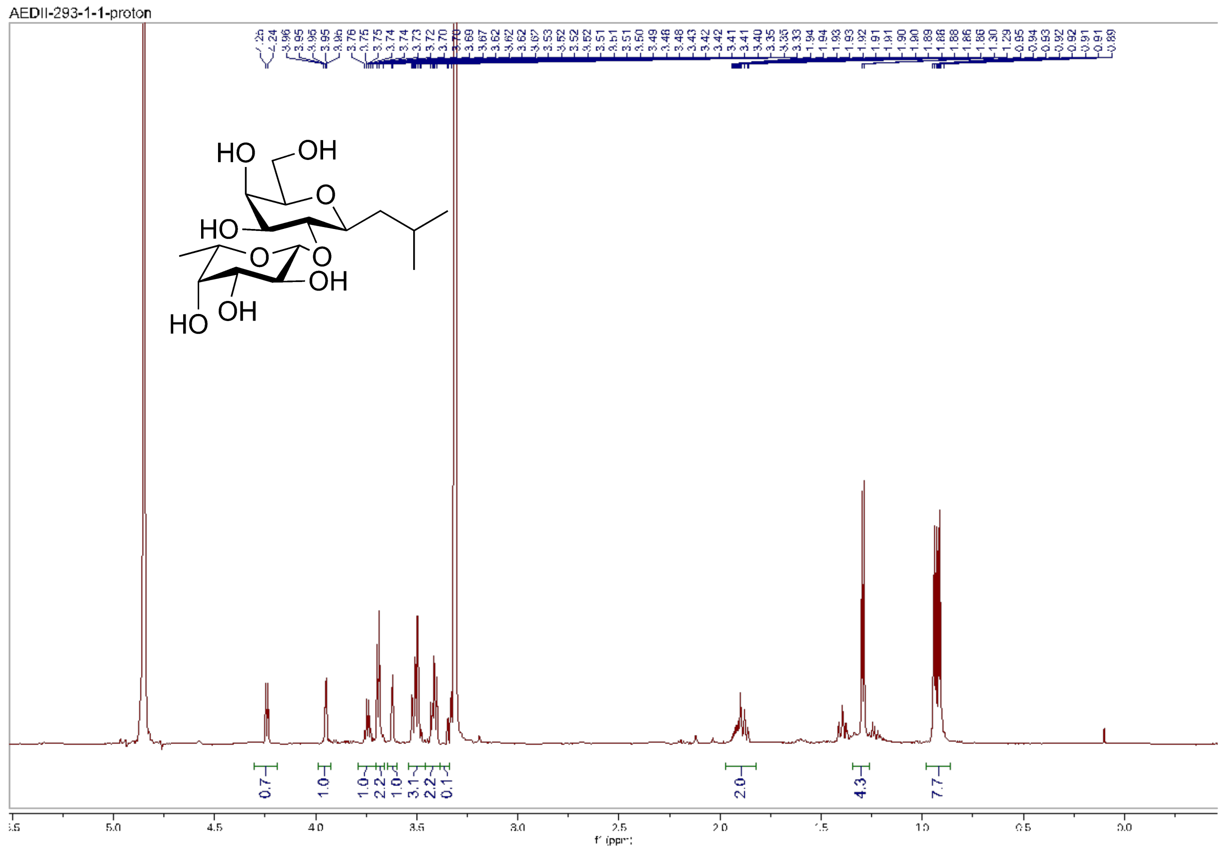

[1] H NMR (600 MHz, CD_3OD) spectrum of compound **2c**

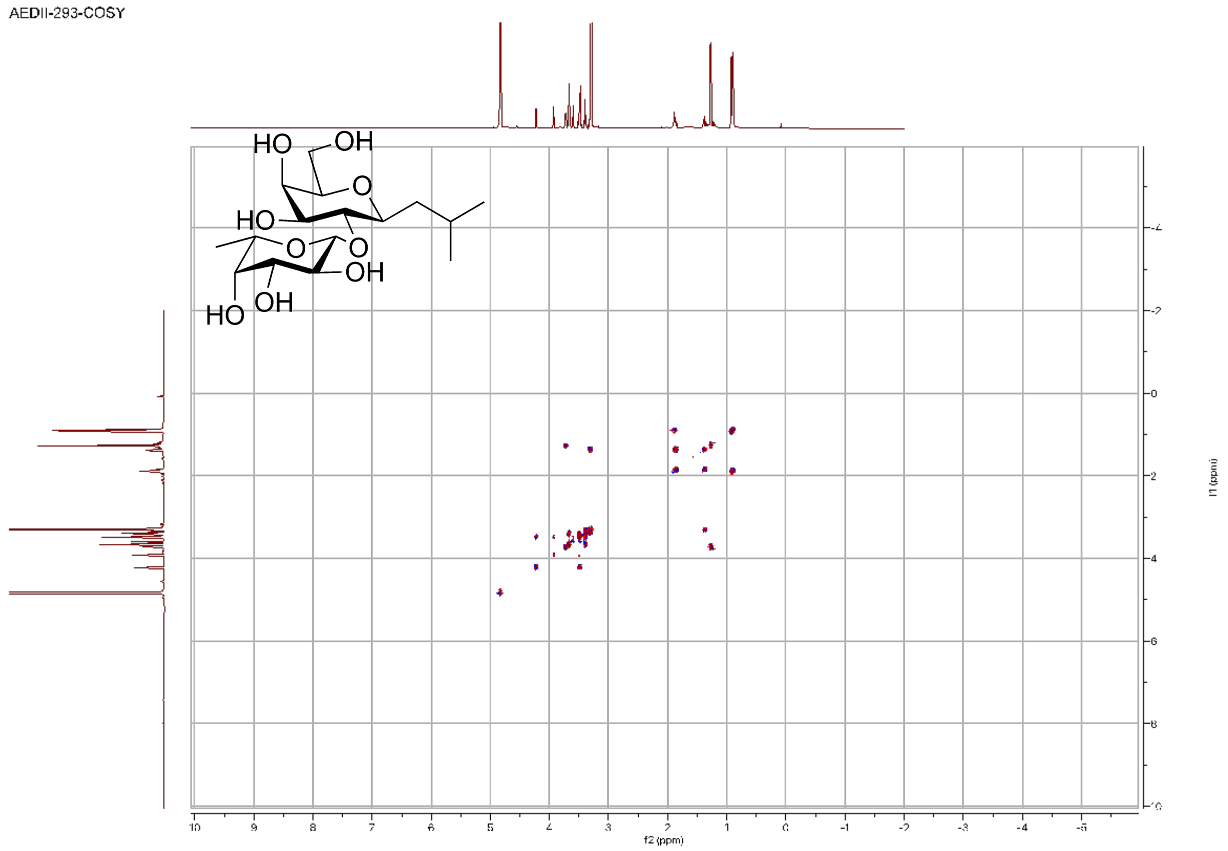

COSY (600 MHz, CD_3OD) spectrum of **2c**

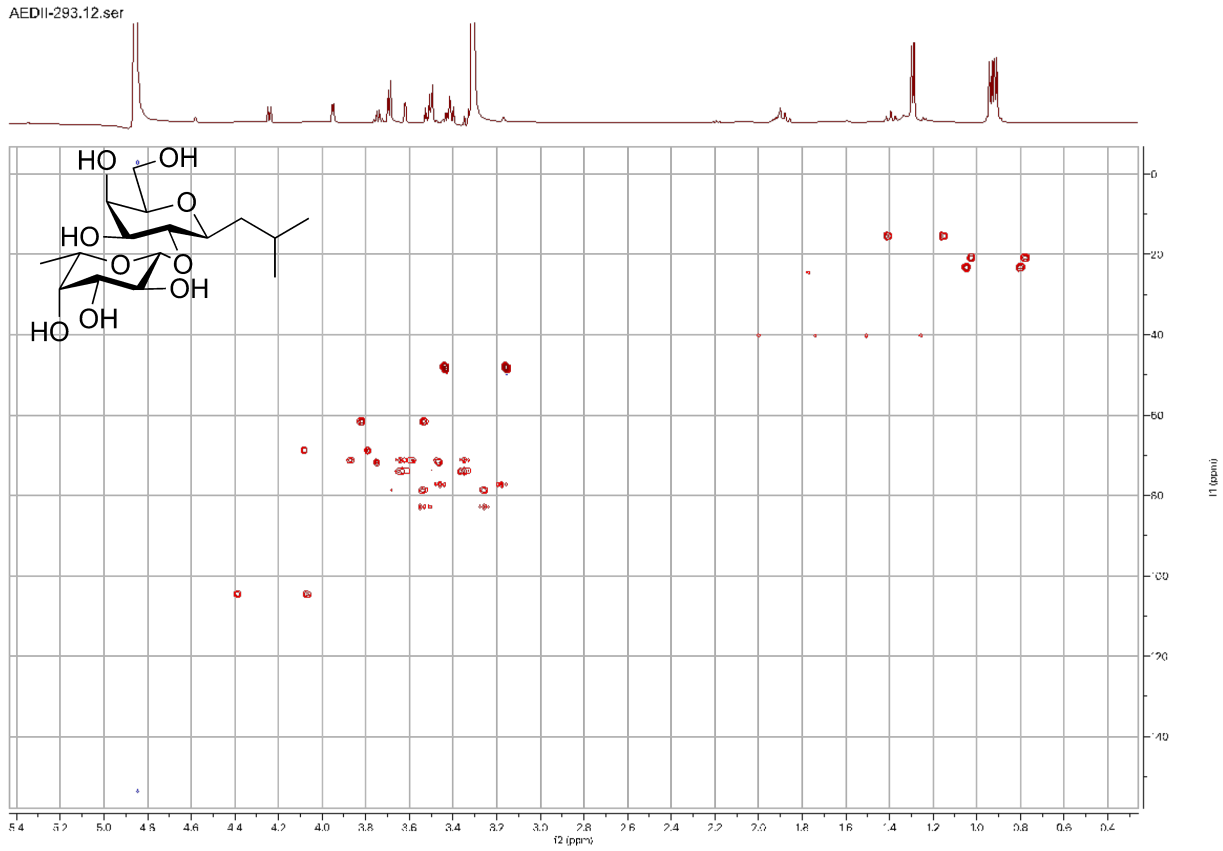

Coupled HSQC (500 MHz, CD_3OD) of compound **2c**

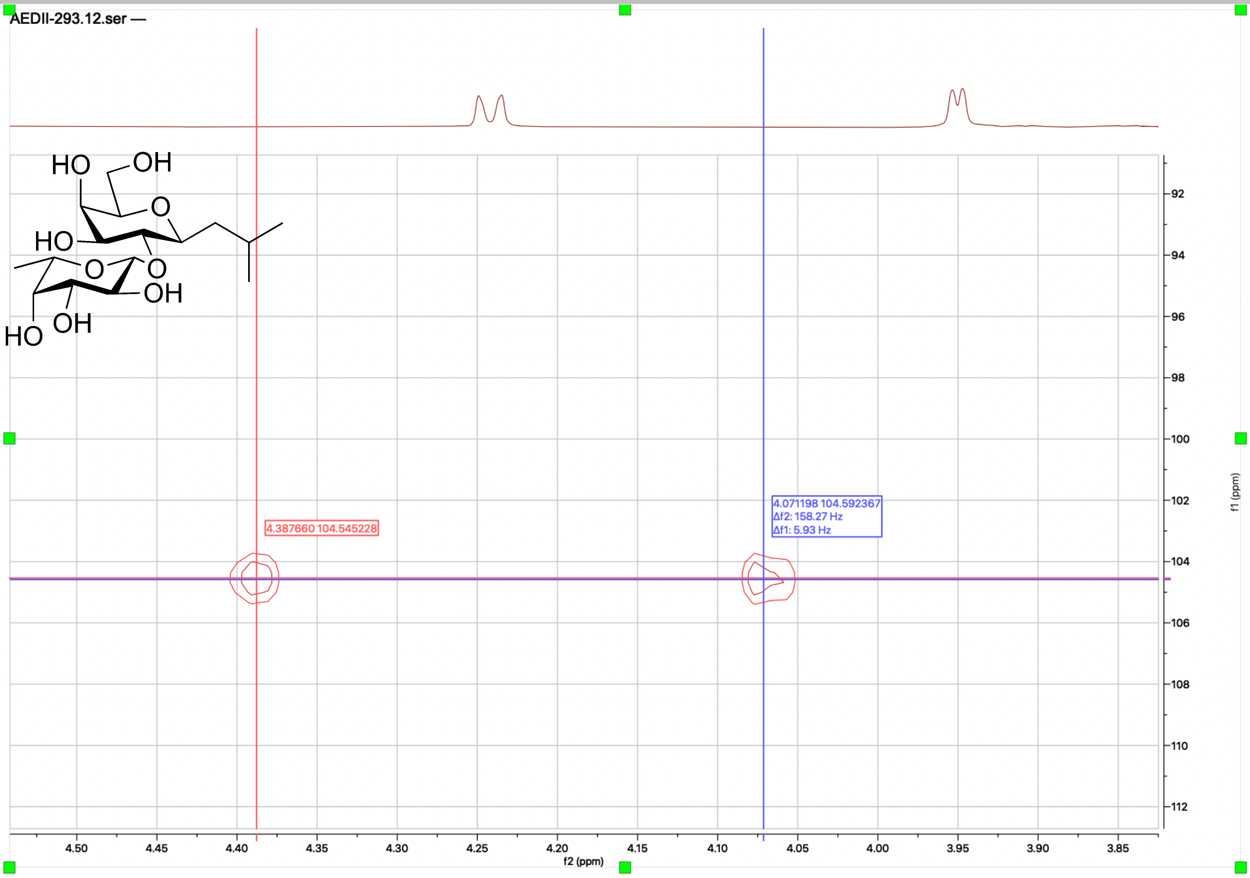

Zoom in of coupled HSQC on anomeric C-H. J value of 158 Hz indicates axial proton.

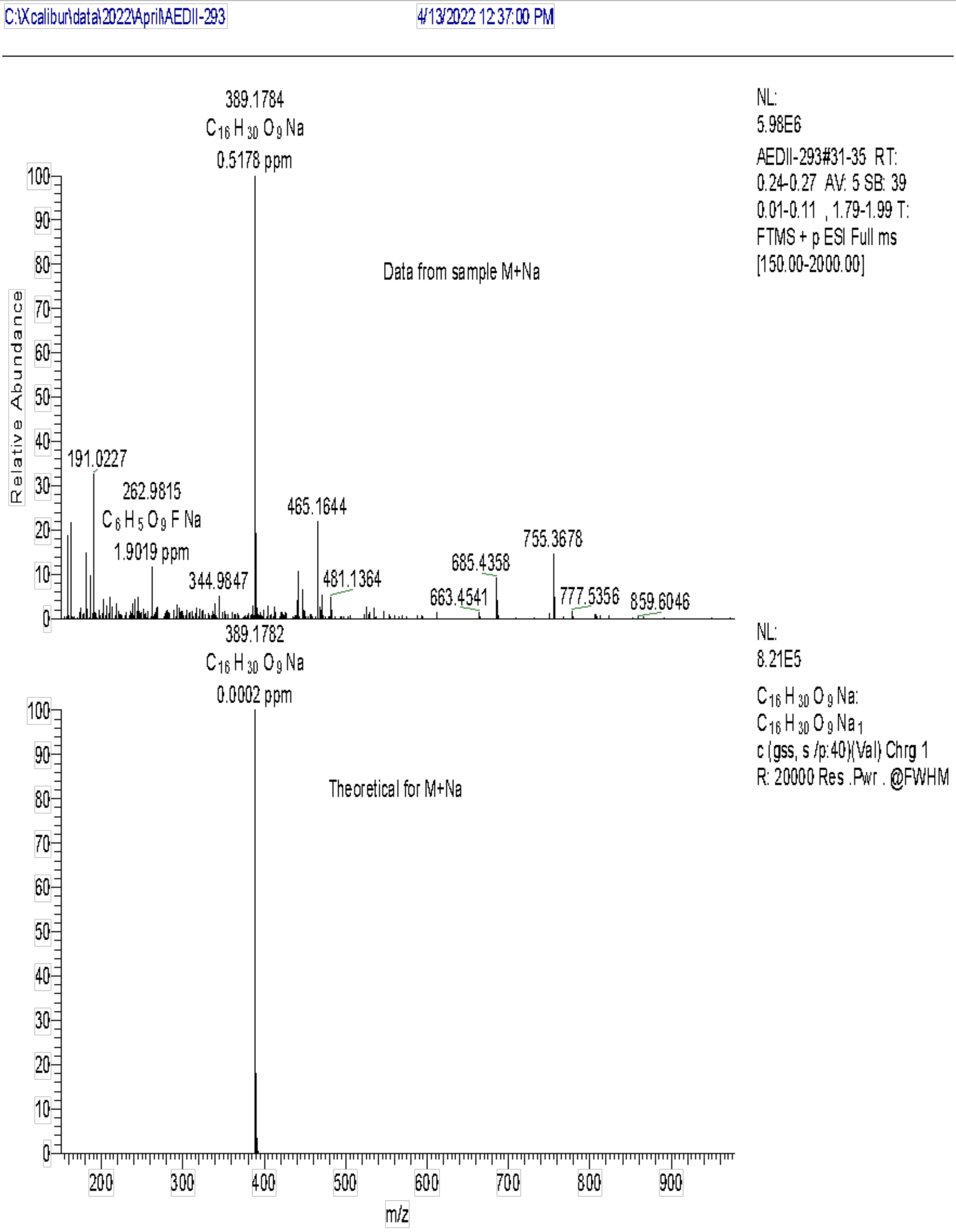

HRMS for compound **2c**

Inducible Protein Expression

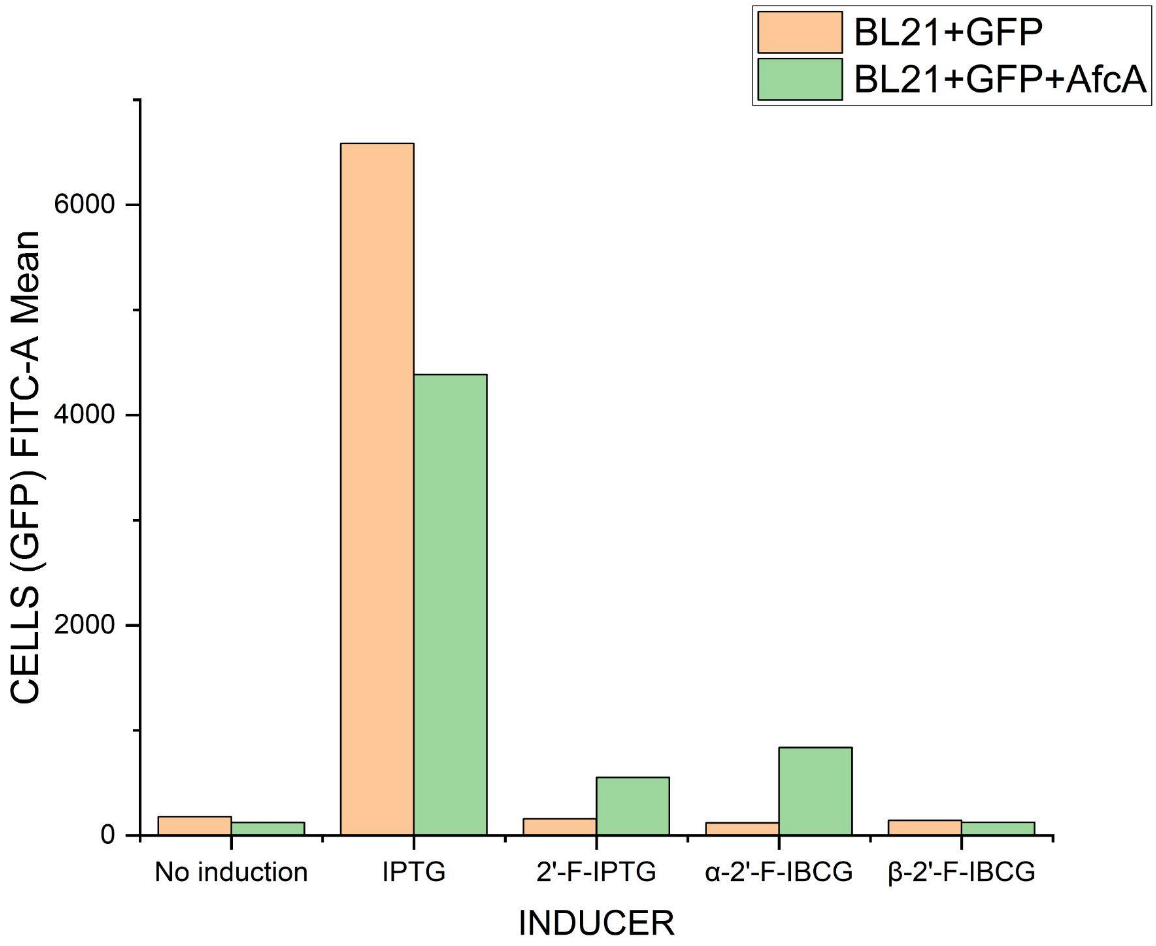

Figure S5. Expression of GFP in the presence or absence of α1-2 fucosidase (AfcA). Inducers were added at 1 mM and GFP expression was measured after 24 h.

Bacterial Strains, Plasmids, and Culture Conditions

E. coli BL21 (DE3) were purchased from New England Biolabs. The plasmid pET28:GFP was a gift from Matthew Bennett and the vector pG9-m2-AfcA was synthesized by Gen9 (Cambridge, MA) as in Enam and Mansell, Cell Chem Biol., 2018. doi: 10.1016/j.chembiol.2018.06.002.[92] Antibiotics including kanamycin (50 mg/L), carbenicillin (100 mg/L) and Isopropyl-β-d-thiogalactoside (IPTG) were supplemented to the growth culture when needed, and purchased from Sigma-Aldrich (Saint Louis, MO, USA). Synthetic inducers for protein expression 2'-Fucosyl IPTG, 2'-Fucosyl IBCG (α and β anomers) were synthesized in this study. Cell harboring the plasmid GFP and AfcA were grown in Luria–Bertani (LB) medium supplemented with respective antibiotics. 1 mL of the cells were grown in sterile 1.5 mL eppendorf tube at 37 °C with agitation at 250 rpm. The cultures were induced at an OD600 of 0.6 with 1mM of IPTG/2'FL-IPTG/2'FL-IBCG (alpha and beta). Samples were collected at regular intervals for figure 2c and after 24 hours for supplementary figure S5. Further the samples were analyzed with a FACS Canto flow cytometer for GFP fluorescence measurement, the total number of events analyzed was 20,000 across all samples.
